# Prenatal cadmium exposure and male infertility in mice: A multi-generational mechanistic study

**DOI:** 10.1016/j.eehl.2026.100217

**Published:** 2026-01-30

**Authors:** Hualong Zhu, Yongwei Xiong, Zhi Yuan, Yexin Luo, Kongwen Ouyang, Tiantian Wang, Hua Wang, Yufeng Zhang, Wei Chang, Jin Zhang, Hao Li, Lan Gao, Dexiang Xu, Hua Wang

**Affiliations:** a Department of Toxicology, School of Public Health, and Center for Big Data and Population Health of IHM, Anhui Medical University, Hefei 230032, China; b Key Laboratory of Environmental Toxicology of Anhui Higher Education Institutes, Hefei 230032, China; c Key Laboratory of Population Health Across Life Cycle (Anhui Medical University), Ministry of Education of the People’s Republic of China, Hefei 230032, China; d Department of Respiratory Medicine, Anhui Provincial Children’s Hospital, Hefei 230032, China

**Keywords:** Multi-generational male infertility, Prenatal Cd exposure, Testosterone deficiency, N^6^-methyladenosine modification, RAPSN

## Abstract

Male infertility affects approximately one in seven couples worldwide. Prenatal cadmium (Cd) exposure has been shown to affect offspring phenotypes and increase susceptibility to diseases later in life. However, the effects of prenatal Cd exposure on multi-generational offspring fertility and the mechanisms remain unknown. A novel murine multi-generational (F1–F3 offspring) male subfertility model induced by prenatal Cd exposure was developed. The levels of testosterone and steroidogenic enzymes were also lower in these offspring’s testes. The ubiquitin-dependent degradation of NR4A1, the upstream transcription factor regulating steroidogenic enzymes, was enhanced across generations upon prenatal Cd exposure. After treatment with MG132, an inhibitor of the ubiquitin-proteasome system, the levels of NR4A1 and steroidogenic enzymes were higher in offspring testes with prenatal Cd exposure. Based on the analysis of the UbiBrowser database and testicular global transcriptome, RAPSN was identified as a novel ubiquitin E3 ligase containing the RING-H2_Rapsyn domain that mediates multi-generational testicular NR4A1 ubiquitination. m^6^A epitranscriptome analysis revealed that prenatal Cd exposure upregulated RAPSN expression in multi-generational offspring testes, and was attributed to a higher level of m^6^A modification of *Rapsn* mRNA. Furthermore, there was a lower level of YTHDC2, a m^6^A reader, in the multi-generational offspring testes with prenatal Cd exposure. Prenatal and postnatal testicular YTHDC2 overexpression reduced the stability of m^6^A-methylated *Rapsn* mRNA to downregulate RAPSN expression in F1–F3 testes. Overall, YTHDC2 reduction-mediated increment in m^6^A-methylated *Rapsn* mRNA contributed to prenatal Cd-enhanced multi-generational susceptibility to male subfertility.

## Highlights

• Prenatal Cd exposure induces male subfertility in multigenerational offspring.
• Testicular NR4A1 ubiquitin drives prenatal Cd-induced male offspring subfertility.
• RAPSN is firstly identified as testicular E3 driving offspring NR4A1 ubiquitin.
• Prenatal Cd enhances m6A modification to elevate RAPSN level in offspring testes.

## Graphical abstract

**Figure:**
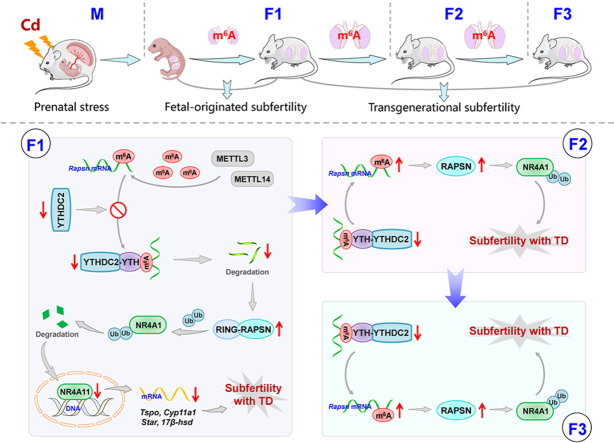

## 1 Introduction

The World Health Organization (WHO) reported that infertility was the third most common global health disorder, after cardiovascular disease and cancer, affecting approximately 17% of the global population [1]. Male-factor infertility accounts for approximately 50% of infertility cases among couples [2]. The incidence of infertility among reproductive-age males increased by 0.291% per year globally between 1990 and 2017 [3]. The developmental origins of health and disease (DOHaD) theory proposes that prenatal exposure to adverse factors promotes the occurrence and development of chronic diseases in adulthood and impacts disease risk across generations [4]. Two animal experiments indicated that prenatal exposure to environmental stressors, such as endocrine disruptors [5] and heavy metals [6], increased infertility in male offspring. Cd, a toxic heavy metal, is widely present in the environment. In Nigeria, India, and Pakistan, Cd concentrations in groundwater have reached 130, 60, and 10 μg/L [7], respectively, and were well above the WHO-recommended limit of 3 μg/L [8]. Most people, including pregnant women, are nonoccupationally exposed to environmental Cd mainly through drinking water, smoking, and diet. Cd intake for the general population in China had doubled from 13.8 to 30.6 μg/day over the last 25 years (1990–2015) [9]. The daily intakes of Cd were 26.4 μg in Japan [10], 10.6–23.0 μg in Korea [11], and over 15 μg in Sweden [12]. These Cd intakes exceeded the Provisional Tolerable Monthly Intake (PTMI) values recommended by the Agency for Toxic Substances and Disease Registry of the U.S. (3 μg/kg per month, which corresponds to 6 μg/day for a person who weighs 60 kg) [9]. Animal experiments showed that prenatal Cd exposure impaired reproductive development and caused subfertility in male offspring [13,14]. Recently, two animal studies further reported that prenatal Cd exposure induced transgenerational inheritance of male subfertility in multi-generational offspring [15,16]. However, the underlying mechanism by which prenatal Cd induces multi-generational male subfertility remains unclear.

Epigenetic modification was a potential mechanism by which prenatal environmental stressors increased multi-generational susceptibility to chronic diseases [17,18]. Previous studies primarily focused on the role of classic epigenetic modifications, including DNA methylation [19] and histone modifications [15], in multi-generational effects caused by developmental Cd exposure. Recently, a genetic analysis suggested that N^6^-methyladenosine (m^6^A) modification might contribute to the inheritance of human diseases [20]. m^6^A was an emerging epigenetic modification and one of the most abundant RNA-methylation modifications in eukaryotic cells [21,22]. Several studies demonstrated that m^6^A modification was involved in the occurrence and development of numerous diseases [[23], [24], [25]]. An animal experiment indicated that m^6^A modification was gradually increased during the process of testicular development, accompanied by an increase in methylase METTL3/14 and a decrease in demethylase ALKBH5 and FTO [26]. A human population study indicated that m^6^A modification was significantly increased in the testes of infertile patients [27]. Animal experiments further reported that m^6^A binding protein YTHDC2 played a crucial role in promoting male testicular development and regulating fertility [28,29]. Recently, an animal study reported that dysregulated m^6^A modification contributed to intergenerational vascular impairment following parental diphenyl phosphate exposure in zebrafish [30]. However, the role of m^6^A modification in elevated multi-generational susceptibility to male subfertility induced by developmental Cd exposure has not been determined.

Here, based on the internal Cd exposure doses in pregnant women, we established a mouse model to explore the effect of prenatal Cd exposure on multi-generational offspring male fertility and the underlying mechanism. Global offspring testicular transcriptome and m^6^A epitranscriptome changes were profiled to evaluate the role of m^6^A modification of key molecules in the above process. *In vivo* testicular gene-specific overexpression, in vitro gene-specific overexpression or knockdown and protein domain mutation, and in silico protein-protein docking were conducted to confirm the mechanism. Our study provides key data for the multi-generational male offspring reproductive toxicity of prenatal Cd exposure and a new insight into the epigenetic mechanism by which prenatal environmental toxicants induce multi-generational offspring diseases.

## 2 Materials and methods

### 2.1 Study strategy

This study aimed to explore the effects and transgenerational mechanisms of prenatal Cd exposure on multi-generational offspring fertility. Pregnant mice were exposed to Cd via drinking reverse osmosis (RO) water containing CdCl_2_ [low-dose Cd (LCd): 50 mg/L; high-dose Cd (HCd): 150 mg/L] from gestation day (GD) 8 to GD17. F1 (filial generation 1) males mated with wild-type (WT) females to generate F2 offspring. Similarly, F3 offspring were generated. Offspring testicular descent rate, anogenital distance (AGD), and anogenital index (AGI; AGD divided by the cube root of weight) were recorded on postnatal day (PND) 22. Testicular injections were performed on PND35 with either normal saline (NS) or proteasome inhibitor MG132, and with adeno-associated type 9 (AAV9)-empty or AAV9-Ythdc2 in F1 males. The offspring sera and testes were collected on PND35 and PND70. The F1 offspring primary Leydig cells were isolated on PND35 and PND70. Testosterone (T) content and synthesis were assessed on PND35 and PND70. Fertility was evaluated on PND70, and sperm count and motility were also analyzed at this stage to assess reproductive capacity. In addition, m^6^A modification and ubiquitination levels were examined in offspring testes on PND35 and PND70. Prenatal N-acetylcysteine (NAC) intervention (500 mg/kg per day) was used to assess protective effects against Cd-induced multi-generational subfertility. Fig. S1 provides an overview of the multi-generational experiment, including the breeding strategy and the timeline of assessments. Furthermore, TM3 cells with specific gene overexpression or knockdown were generated to further validate the underlying mechanism.

### 2.2 Dose selection of Cd

The internal exposure dose provides a more accurate reflection of the biologically effective dose [31,32]. Because mice (18 days) [33] have a much shorter gestation than humans (280 days) [34], the dose of Cd was selected based on the internal exposure dose in the human body. We performed the reverse dosimetry approach to estimate Cd intake in mice, aiming to replicate serum concentrations relevant to humans. Serum Cd levels (3.37 ± 2.58 μg/L) were reported in pregnant women who delivered babies with low birth weight [35]. Considering the mouse oral absorption rate of Cd (about 2%) [36], the fraction of total body Cd present in serum (estimated value is 0.005%) [37], and the serum volume of pregnant mice on GD18 (about 2.2 mL) [38], the total Cd intake should be 7.41 mg to achieve a serum Cd concentration of 3.37 μg/L in mice. Finally, the Cd concentration in drinking water was calculated to be 148 mg/L based on the exposure period (GD8–GD17) and the average daily water intake of the mice (about 5 mL). In the current study, Cd concentration in maternal sera on GD18 was 4.57 ± 0.48 μg/L in the HCd (150 mg/L) group (Fig. S1G–H). This maternal serum Cd level was similar to those (3.37 ± 2.58 μg/L) in pregnant women who delivered babies with low birth weight [35], comparable to the blood Cd concentrations of current smokers in Pakistan (3.54 ± 0.38 μg/L) [39] and China (median 2.58 μg/L, range 0.95–5.36 μg/L) [40], and below the Occupational Safety & Health Administration (U.S.) medical surveillance threshold of 5 μg/L [41]. As above, the maternal sera Cd concentration in our model was applicable to the internal exposure dose in the human body.

### 2.3 Animal experiments

All CD-1 mice were supplied by Beijing Vital River (Beijing, China) and kept under standard conditions (12 h light/12 h dark cycle, 20–25 °C, and 50%–60% air humidity) with adequate food and water for two weeks. Two males and four females were placed in a cage to mate for one night. The females with a vaginal plug on the next morning were fertilized, and the day was recorded as GD0. Five pregnant mice were housed together from GD0 to GD17, and the number was reduced to one per cage on GD18 to ensure smooth delivery. All mice were anesthetized with 2,2,2-tribromoethanol, and then euthanized. All animal experiments followed the Association of Laboratory Animal Sciences guidelines at Anhui Medical University for humane treatment (Ethical approval number: LLSC20221314). The animal study was divided into five experiments.

**Experiment 1.** Pregnant mice were randomly divided into three groups: control (Ctrl), LCd, and HCd groups; n = 30 per group. In the LCd and HCd groups, the mice were exposed to Cd via drinking RO water containing CdCl_2_ (LCd: 50 mg/L; HCd: 150 mg/L) from GD8 to GD17. The pregnant mice drinking RO water were regarded as control. To explore the effect of prenatal Cd on fetal growth, 15 pregnant mice in each group were randomly euthanized. The fetal size was recorded, and the fetal sera and testes were collected. The remaining pregnant mice (n = 15 per group) delivered naturally. After birth, the number of offspring was adjusted to 5 males and 5 females in each litter. All offspring were weaned on PND21. For each F1 litter, one mouse was randomly selected for euthanasia on either PND35 or PND70, while another male was mated with two WT females to generate F2 offspring. Following the above process, F3 offspring were generated. Offspring testicular descent rate, AGD, and AGI were recorded on PND22. The sera and testes were collected on PND35 and PND70.

**Experiment 2.** Pregnant mice were randomly divided into two groups: Ctrl and HCd groups; n = 12 per group. Following the protocol described in Experiment 1, F1 males were generated. The F1 male offspring were injected with proteasome inhibitor MG132 (1 μg/testis per 5 days) from PND35 to PND70. Ten F1 males from Ctrl litters were injected with equal normal saline (NS, 10 μL/testis) and regarded as the Ctrl group. All F1 males underwent testicular injection under anesthesia. MG132 was dissolved in dimethyl sulfoxide (100 mg/mL) and diluted to a working solution (0.1 mg/mL) using NS. The F1 male sera and testes were collected on PND70.

**Experiment 3.** To determine whether RAPSN is a neoteric ubiquitin E3 ligase targeting NR4A1 in testes, testicular RAPSN-overexpressing male mice were produced via testicular injection of lentivirus encoding *Rapsn.* 5-week-old WT male mice were randomly divided into three groups: Ctrl, type 5 lentivirus (LV5)-empty, and LV5-*Rapsn* groups; n = 10 per group. As described in Section 2.4, testicular RAPSN-overexpressing male mice were produced. The sera and testes were collected after five weeks of testicular injection.

**Experiment 4.** Animal Experiments 2 and 4 were conducted simultaneously, using F1 males from the same batch of maternal mice. To evaluate the role of YTHDC2 reduction in prenatal Cd-induced m^6^A modification of *Rapsn* mRNA in F1 males, testicular *Ythdc2*-overexpressing mice were generated via testicular injection with AAV9-*Ythdc2* on PND 35, as described in Section 2.4. The F1 male sera and testes were collected on PND70.

**Experiment 5.** Pregnant mice were randomly divided into four groups: Ctrl (n = 30), N-acetylcysteine (NAC), HCd, and NAC + HCd (NH) groups; n = 30 per group. In HCd and NH groups, the pregnant mice were exposed to HCd (150 mg/L) via drinking RO water with or without NAC supplementation (500 mg/kg per day, i.g.) from GD7 to GD17. The pregnant mice drinking RO water were regarded as Ctrl. Experiment 5 was performed following the protocol described in Experiment 1. 15 pregnant mice from each group were randomly euthanized on GD18. The fetal size was recorded, and the fetal sera and testes were collected. The remaining pregnant mice gave birth naturally to F1 offspring. Each F1 male was mated with two untreated WT females to generate F2 offspring. Similarly, each F2 male was mated with two untreated WT females to generate F3 offspring. The offspring testicular descent rate, AGD, and AGI were recorded on PND22. The offspring sera and testes were collected on PND35 and PND70.

### 2.4 Generation of testicular *Rapsn*- or YTHDC2-overexpressing mice

The testis-specified gene-overexpressing mice were produced as previously described [42,43]. The mice were anesthetized using 2,2,2-tribromoethanol (250 mg/kg, i.p.). A lentivirus (LV) or adeno-associated virus (AAV) suspension was injected into the mouse testes using a 30G needle syringe to produce testis-specified gene-overexpressing mice. Each testis was injected with a 10 μL suspension of LV5 encoding *Rapsn-*GFP (1 × 10^9^ TU/mL) to generate testicular RAPSN-overexpressing mice. For the generation of testicular YTHDC2-overexpressing mice, each testis was injected with 10 μL suspension of AAV9 encoding *Ythdc2* (2 × 10^11^ VG/mL). All mice were kept under standard conditions with adequate food and water for more than five weeks after testicular injection. The animals were then euthanized, and the testes were collected for further analysis.

### 2.5 Mouse primary Leydig cells isolation

The mouse testicular primary Leydig cells were isolated according to previous research [44,45]. In brief, the left testicular tissue from 5 male mice was pooled to isolate the mouse primary Leydig cells. Testicular tissue was minced and digested in a DMEM/F12 medium containing trypsin (1 mg/mL) and DNase I (0.3 mg/mL) at 37 °C for 30 min. One-tenth of the mixed digests were used as unpurified extracts. The remaining mixture was purified using a 10%–60% Percoll gradient with 10% step increments. The cells in the 50%–60% Percoll gradient were collected. After the separation, proteins were directly extracted from the cells. The levels of 3β-HSD, MVH, and SOX9 in the purified and unpurified extracts were measured to determine the purity.

### 2.6 m^6^A-mRNA epitranscriptomic microarray and genome-wide transcriptional profiling

m^6^A-mRNA epitranscriptomic microarray and genome-wide transcriptional profiling analysis were performed by Aksomics Company (Shanghai, China) as previously described [46,47]. Total RNA was extracted using TRIzol reagent (Invitrogen) according to the manufacturer’s instructions. The RNAs were quantified using the NanoDrop ND-1000. The RNA integrity was evaluated using Bioanalyzer 2100 (Agilent). The total RNAs were treated with anti-m^6^A antibodies for immunoprecipitation. The modified and unmodified RNAs were regarded as the “IP” and “Sup”. According to the Arraystar RNA Labeling protocol, the “IP” RNAs were labeled as Cy5 cRNAs, and the “Sup” were labeled as Cy3 cRNAs. The RNAs were then hybridized with Mouse mRNA&lncRNA Epitranscriptomic Microarray (8 × 60K, Arraystar). Finally, the arrays were scanned using an Agilent Scanner G2505C. The acquired array images were analyzed using Agilent Feature Extraction software (version 11.0.1.1). “Expression level” was calculated based on the IP and Sup normalized intensities. “m^6^A quantity” was calculated for the m^6^A methylation amount based on the IP normalized intensities. Differentially expressed genes and m^6^A-methylated RNAs between the two comparison groups were identified by filtering for *P* < 0.05 and log_2_(|Fold Changes (FC)|) > 0.5 and visualized by heatmap using MEV software (https://webmev.tm4.org). Gene ontology analysis was performed to identify certain gene ontological functions and gene ontology (GO) terms enriched among the differentially m^6^A-methylated mRNAs (accessed June 11, 2023). The GEO database (GSE289472, https://www.ncbi.nlm.nih.gov/geo/query/acc.cgi?acc&equals;GSE289472) provided the raw data.

### 2.7 Methylated RNA immunoprecipitation (MeRIP)-qPCR

MeRIP-qPCR was performed according to published studies [48]. The total testicular RNAs were extracted using TRIzol reagent (Invitrogen) according to the manufacturer’s instructions. The mRNA was isolated from total RNA using the Seq-StarTM poly(A) mRNA Isolation kit (Arraystar). The purified mRNA was then cut into 100-nucleotide fragments using the RNA Fragmentation Reagents (Invitrogen). About 10% of the purified mRNA fragments were used as the control. The remaining mRNA fragments were combined with the 2 μg rabbit-derived polyclonal antibody against m^6^A (Synaptic System) and Sheep anti-Rabbit IgG Dynabeads (ThermoFisher). The bead-antibody-RNA mixture was incubated in a 500 μL IP reaction system. The bound mRNA fragments were extracted using the RNeasy Mini Kit (Qiagen) after washing and elution. The mRNA was reverse-transcribed into cDNA using Transcriptor First Strand cDNA Synthesis Kit (04896866001, Roche). We predicted four highly credible m^6^A-modified sites located in a consensus “RRACH” motif (R = G or A, H = A, C, or U) of *Rapsn* mRNA using the SRAMP database (http://www.cuilab.cn/sramp). The primer sequences of each site are summarized in Table S4.

### 2.8 RNA immunoprecipitation (RIP)

RIP was performed using a BeyoRIP™ RIP Assay Kit (P1801S, Beyotime) according to the manufacturer’s protocol. Firstly, 30 mg of testicular tissue was lysed using lysis buffer supplemented with protease and RNase inhibitors. The lysate was incubated with Protein A/G Magnetic Beads for 1 h at 4 °C to reduce non-specific binding. Then, the lysate was incubated with the antibodies against YTHDC2 or IgG for 4 h at 4 °C. The immune complexes were captured by adding Protein A/G Magnetic Beads and incubating overnight at 4 °C. After washing 4 times, the beads were incubated in elution buffer for 30 min at 55 °C to elute RNA. The RNA was extracted using a RNeasy Mini Kit (74104, Qiagen). The RNA was then reverse-transcribed into cDNA using Transcriptor First Strand cDNA Synthesis Kit (04896866001, Roche). RT-qPCR was performed to determine the relative interaction between the YTHDC2 protein and *Rapsn* mRNA.

### 2.9 Protein-protein docking analysis

The amino acid sequences of mouse RAPSN (UniProtKB: Q2M2N6) and NR4A1 (UniProtKB: P12813) were obtained from the UniProt database (https://www.uniprot.org; accessed January 2024). The three-dimensional (3D) structure models of mouse RAPSN and NR4A1 were constructed using the SWISS-MODEL web server (https://swissmodel.expasy.org; accessed February 28, 2024) via homologous modeling. The 3D structure model of RAPSN was docked onto the 3D structure model of NR4A1 using the HDOCK web server (http://hdock.phys.hust.edu.cn; accessed September 9, 2023). RAPSN was recognized as the receptor, and NR4A1 as the ligand. HDOCK yielded the docking scores to evaluate the confidence of the protein-protein docking models. A negative docking score indicates a more favorable binding model. The highest-ranked protein complex with the lowest docking score was selected in this study. The interaction surface analysis for the selected protein-protein docking model was performed using the PDBePISA web server (https://www.ebi.ac.uk/pdbe/pisa; accessed February 2024). The protein-protein docking model of RAPSN and NR4A1 was visualized using PyMOL 4.6.0 software.

### 2.10 Co-immunoprecipitation (Co-IP)

Co-IP was performed as previously described [49]. Protein lysates from mouse testes were prepared using IP lysis buffer (P0013, Beyotime) containing protease inhibitors. The total testicular protein (1000 μg) was incubated with protein A/G magnetic beads (HY-K0202, MedChemExpress) for 1.5 h at 4 °C to remove nonspecific binding. The tubes were placed on a magnetic stand to collect the precleared protein lysates. The samples were precipitated overnight at 4 °C with an NR4A1 antibody (1 μg) and protein A/G magnetic beads. The protein A/G magnetic beads were then collected and washed with precooled IP lysis buffer. The beads were boiled in loading buffer at 100 °C for 10 min to elute bound proteins. Finally, the levels of Ub and RAPSN were measured using immunoblotting.

### 2.11 Statistical analysis

SPSS 23.0 software was used for statistical analyses. All data were presented as means ± SEM (standard error of mean). An independent sample *t*-test was used to compare the two groups. One-way ANOVA followed by Bonferroni’s or Tamhane’s T2 post hoc test was used to compare multiple groups. Repeated-measures ANOVA was applied to analyze the repeated-measurement data between two groups. The correlation between the two groups was analyzed by Spearman correlation. *P* < 0.05 was considered statistically significant.

## 3 Results

### 3.1 Prenatal Cd exposure impairs testosterone synthesis and male fertility

**Fig. 1:**
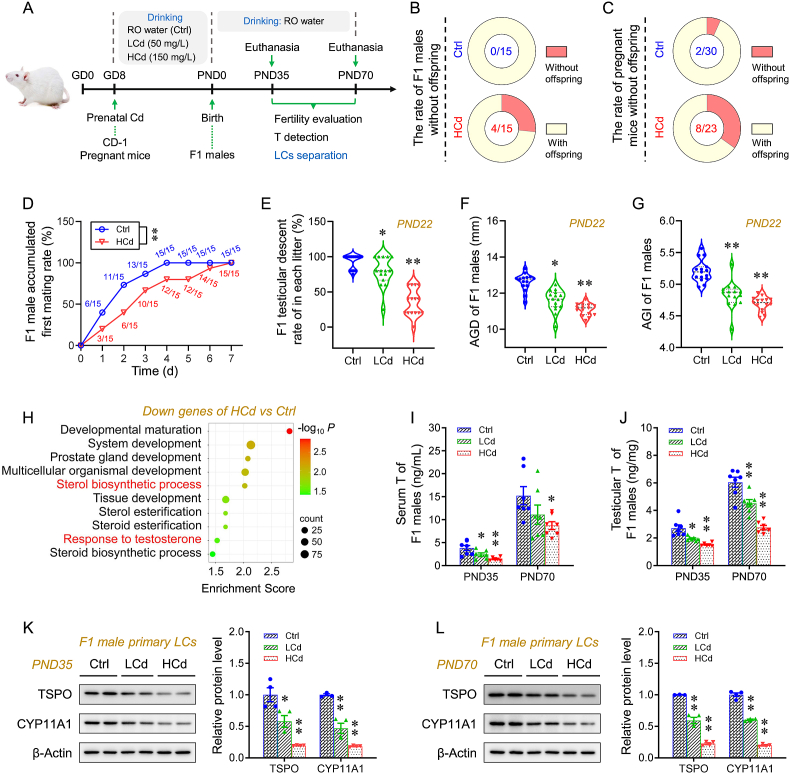
Prenatal Cd exposure impairs testosterone synthesis and male fertility. According to Experiment 1, pregnant mice were exposed to different-dose Cd, and F1 males were euthanized for sera and testes collection or mated with untreated WT females to access fertility. (A) Experimental schematic. (B) Rate of F1 males without offspring (n = 15 per group). (C) Rate of pregnant mice without offspring (Ctrl: n = 30; HCd: n = 23). (D) First-mating rate of F1 males within 7 days (n = 15 per group). (E) F1 testicular descent rate in each litter (n = 15 per group). (F, G) F1 male AGD and AGI (n = 15 per group). (H) Top enriched GO terms among downregulated genes in the global transcriptome of F1 testes on PND70 (n = 3 per group). (I, J) T concentrations in sera and testes (n = 7 per group). (K, L) TSPO and CYP11A1 expression in the primary Leydig cells (n = 4 per group) isolated from F1 testes. Data were analyzed using Repeated-measures ANOVA (D), one-way ANOVA (E−G and I−L), and presented as means ± SEM. Numeric data are provided in Table S6. ∗*P* < 0.05, ∗∗*P* < 0.01, compared to Ctrl.

Pregnant mice were exposed to Cd by drinking water from GD8 to GD18 (Fig. 1A). Fetal weight and fetal crown-rump length in the Cd group were lower than those in the Ctrl group (Fig. S2A–E). The weight of F1 males was also persistently lower (Fig. S2F). Fifteen F1 males from different litters in each group were mated with WT untreated females at a ratio of 1:2. In the Cd group, more F1 males did not generate F2 offspring (Fig. 1B). The number of F2 offspring from F1 males prenatally exposed to Cd was also lower than that in the Ctrl group (Fig. S3A). Prenatal Cd exposure significantly decreased both sperm motility and sperm count in F1 males (Fig. S3C–E). In addition, the expression of Sertoli cell marker SOX9 was comparable across all groups, but reduced expression of germ cell marker MVH was observed in the Cd group (Fig. S3F). We further detected mRNA levels of germ cell markers, including Plzf (spermatogonia), c-Kit (differentiated spermatogonia), Smc3 (spermatocytes), Acrv1 (round spermatids), and Lzumo3 (elongated spermatids). Except for c-Kit, all other markers were downregulated in F1 testes, suggesting that prenatal Cd impairs the entry of differentiated spermatogonia into meiosis in offspring testes (Fig. S3G). The F1 male first-mating rate and the female pregnancy rate were also lower (Figs. 1D and S3B). The higher rate of pregnant mice without offspring was observed in the Cd-exposed group (Fig. 1C). Prenatal Cd significantly delayed testicular descent in F1 males (Figs. 1E and S3H). AGD and AGI were also lower (Fig. 1F and G). Prostatic weight and coefficient in male offspring prenatally exposed to Cd were also lower (Fig. S3I and J). The profiling of the global transcriptome for F1 testes was then performed on PND70. GO analysis revealed that downregulated genes were enriched in the “sterol biosynthetic process pathway” and “response to T pathway” in offspring testes with prenatal Cd exposure (Fig. 1H). Prenatal Cd exposure resulted in lower levels of dihydrotestosterone (DHT) and ubiquitin-conjugating enzyme (E2), two metabolites of T, in F1 sera and testes (Fig. S4A–E). However, the expression of SRD5A1, a key enzyme metabolizing T to DHT, did not differ among the groups (Fig. S4C). Meanwhile, the expression of CYP19A1, a key enzyme metabolizing T to E2, also did not differ among the groups (Fig. S4F). The Cd group showed lower T levels in F1 sera and testes than the Ctrl group. (Fig. 1I and J). The levels of testicular steroidogenic enzymes TSPO, CYP11A1, StAR, and 17β-HSD were also lower (Fig. S4G–K). The Leydig cells were isolated from F1 testes and total protein was directly extracted. The major components of the extract were Leydig cells, as evidenced by the decreased expression of SOX9 (a marker of Sertoli cells) and MVH (a marker of germ cells), and increased expression of 3β-HSD (a marker of Leydig cells) (Fig. S4L). The expression of TSPO and CYP11A1 in F1 Leydig cells was also lower following prenatal Cd exposure (Fig. 1K and L).

### 3.2 NR4A1 ubiquitination drives prenatal Cd-inhibited testosterone synthesis

**Fig. 2:**
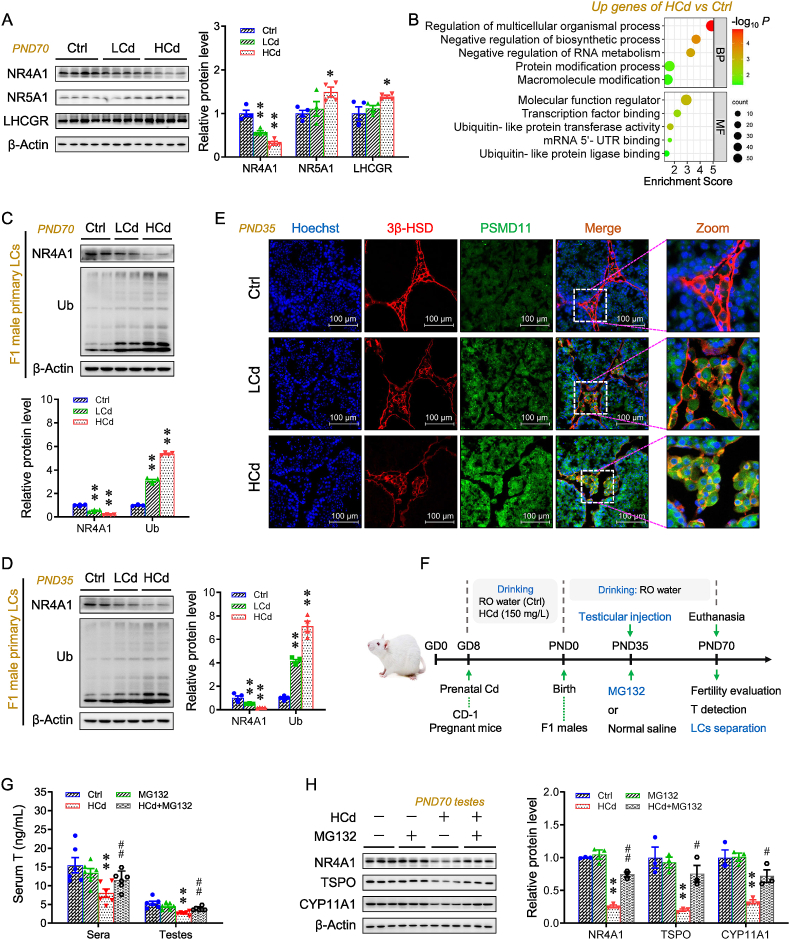
NR4A1 ubiquitination drives prenatal Cd-inhibited testosterone synthesis. (A–E) According to Experiment 1, pregnant mice were exposed to different-dose Cd, and F1 males were euthanized for sera and testes collection or mated with untreated WT females to access fertility. (A) NR4A1, NR5A1, and LHCGR protein expression in F1 testes on PND70 (n = 4 per group). (B) Top enriched GO terms for upregulated genes in the global transcriptome of F1 testes on PND 70 (n = 3 per group). (C, D) NR4A1 and Ub expression in the primary Leydig cells separated from the F1 testes (n = 4 per group). (E) Immunofluorescent analysis for 3β-HSD and PSMD11 in PND35 testes (n = 4 per group). (F–H) According to Experiment 2, pregnant mice were exposed to HCd, and F1 males were injected with MG132 in the testes and then euthanized for sera and testes collection. (F) Experimental schematic. (G) T levels in sera and testes (n = 6 per group). (H) NR4A1, TSPO, and CYP11A1 protein expression in testes (n = 4 per group). Data were analyzed using one-way ANOVA and presented as means ± SEM. Numeric data are provided in Table S7. ∗*P* < 0.05, ∗∗*P* < 0.01, compared to Ctrl; ^#^*P* < 0.05, ^##^*P* < 0.01, compared to HCd group.

Two nuclear receptors, NR4A1, NR5A1, along with LHCGR, promote the transcription of T synthetase genes, including *Tspo*, *Cyp11a1*, *Star,* and *17β-hsd* [50]. NR4A1 expression was lower in F1 testes with prenatal Cd exposure, both on PND35 and PND70, whereas the NR5A1 and LHCGR levels were higher on PND70 (Figs. 2A and S5A). The protein reduction results from transcription and translation inhibition [51], or protein degradation via autophagy and ubiquitin‒proteasome system (UPS) [52]. The *Nr4a1* mRNA and p-eIF2α protein levels did not differ among the groups, indicating that the transcription and translation of *Nr4a1* were not altered (Fig. S5B and D). The reduction in LC3B-II (a marker of autophagosomes) indicated that NR4A1 degradation was not due to autophagy enhancement (Fig. S5D). GO analysis indicated that the upregulated genes were enriched in “protein modification process pathway” and “ubiquitin-like protein transferase activity” in prenatal Cd-exposed offspring testes (Fig. 2B). Global transcriptome profiling also showed that the mRNA levels of proteasome markers, including *Psmd6*, *Psmd7*, *Psmd11,* and *Psmd1*, were higher in these testes (Fig. S6A). Further data confirmed the above changes (Fig. S6B and D). The prenatal Cd-exposed group exhibited lower NR4A1 expression and higher Ub expression in F1 male primary Leydig cells relative to the Ctrl group (Figs. 2C–E and S6E,F). F1 male testes were injected with proteasome inhibitor MG132 to explore the role of higher Ub in T synthesis inhibition upon prenatal Cd exposure (Fig. 2F). Postnatal MG132 treatment reversed the T reduction in F1 male sera and testes induced by prenatal Cd exposure (Fig. 2G). Testicular NR4A1, TSPO, CYP11A1, and StAR levels were higher in the MG132 + HCd group than those in the HCd group (Figs. 2H and S6A). The immunoblotting for Ub indicated that MG132 was effective (Fig. S7A). Moreover, prostate size was also elevated in the presence of MG132 (Fig. S7B and C).

### 3.3 Identification of a candidate gene mediating prenatal Cd-induced NR4A1 ubiquitination in offspring testes

**Fig. 3:**
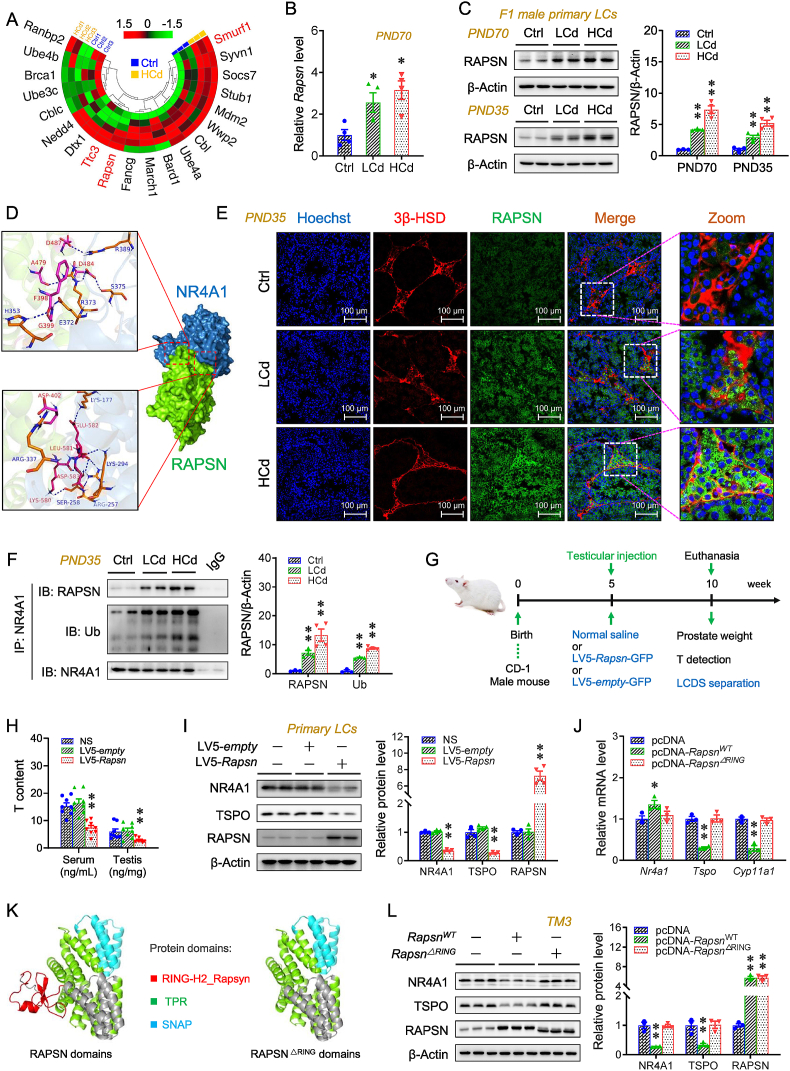
Identification of a candidate gene mediating prenatal Cd-induced NR4A1 ubiquitination in offspring testes. (A–F) According to Experiment 1, pregnant mice were exposed to different-dose Cd, and F1 males were euthanized for sera and testes collection or mated with untreated WT females to access fertility. (A) Heatmap of potential top 20 E3s for NR4A1 in PND70 testes (n = 4 per group). (B) Relative *Rapsn* mRNA level in F1 testes (n = 4 per group). (C) RAPSN expression in the primary Leydig cells on PND35 and PND70 (n = 4 per group). (D) RAPSN-NR4A1 docked surface view. (E) Immunofluorescent analysis of 3β-HSD and RAPSN in PND35 testes (n = 4 per group). (F) Co-IP analysis between NR4A1 and RAPSN or Ub in F1 testes (n = 4 per group). (G–I) According to Experiment 3, the testicular RAPSN-overexpressing mice were generated. (G) Experimental schematic. (H) T content in sera and testes (n = 8 per group). (I) NR4A1, TSPO, and RAPSN expression in the primary Leydig cells (n = 4 per group). (J–L) According to Supplementary Cell experiments, the RAPSN^WT^ or RAPSN^ΔRING^-overexpressing TM3 cells were generated. (J) Relative *Nr4a1*, *Tspo,* and *Cyp11a1* mRNA levels in the cells. (K) RAPSN and RAPSN^ΔRING^ protein domains cartoon view. (L) RAPSN, NR4A1, and TSPO expression in the cells (n = 3 per group). Data were analyzed using one-way ANOVA and presented as means ± SEM. Numeric data are provided in Table S8. ∗*P* < 0.05, ∗∗*P* < 0.01, compared to Ctrl/NS/pcDNA-empty group.

The top 20 potential E3 ubiquitin ligases (E3s) for NR4A1 were predicted using the UbiBrowser database (Fig. S8A). Global transcriptome analysis of these E3s indicated that prenatal Cd exposure led to higher expressions of *Rapsn* (1.93-fold), *Ttc3* (1.28-fold), and *Smurf1* (1.32-fold) in F1 testes (Fig. 3A). The prenatal Cd-exposed group also exhibited higher expression of RAPSN in F1 testes and primary Leydig cells relative to the Ctrl group (Figs. 3B,C,E and S8B,C). A model of NR4A1−RAPSN interaction was established through in silico docking analysis. The highest-scoring docking model included several residues responsible for the interaction at the modeled interface (Fig. 3D). Co-IP analysis demonstrated that testicular NR4A1 interacted with RAPSN and Ub under physiological conditions, and that this interaction was further strengthened in the testes of offspring prenatally exposed to Cd (Fig. 3F). Testicular RAPSN-overexpressing mice were generated via testicular injection of a lentivirus encoding *Rapsn* (Fig. 3G). The lower levels of NR4A1, TSPO, and CYP11A1 were observed in RAPSN-overexpressing testes (Figs. 3I and S9A-C,E). The higher Ub expression was observed in RAPSN-overexpressing testes and primary Leydig cells (Fig. S9D and E). The prostate size was also reduced in RAPSN-overexpressing mice (Fig. S9F and G). There are three types of E3s, namely, those containing HECT, RING, and U-box domains. A model of RAPSN protein domains was constructed using the UniProtKB database and visualized in PyMOL software. The model identified three major protein domains: RING-H2_Rapsyn, TPR, and SNAP (Fig. 3K). We synthesized plasmids expressing RAPSN^ΔRING^ (RAPSN deleted RING-H2_Rapsyn domain) or RAPSN^WT^ (wild-type RAPSN protein) to transfect TM3 cells (Figs. 3K and S10A). The expression of TSPO and CYP11A1, and the level of T in culture medium were lower in RAPSN^WT^-overexpressing cells (Figs. 3J,L and S10B-D). Moreover, the lower levels of NR4A1 and the higher expression in Ub occurred in RAPSN^WT^-overexpressing cells (Figs. 3L and S10C,D). Interestingly, the above changes in RAPSN^WT^-overexpressing cells were not observed in the RAPSN^ΔRING^-overexpressing cells (Figs. 3J-L and S10B-D).

### 3.4 Prenatal Cd stabilizes *Rapsn* mRNA by reducing YTHDC2 in offspring testes

**Fig. 4:**
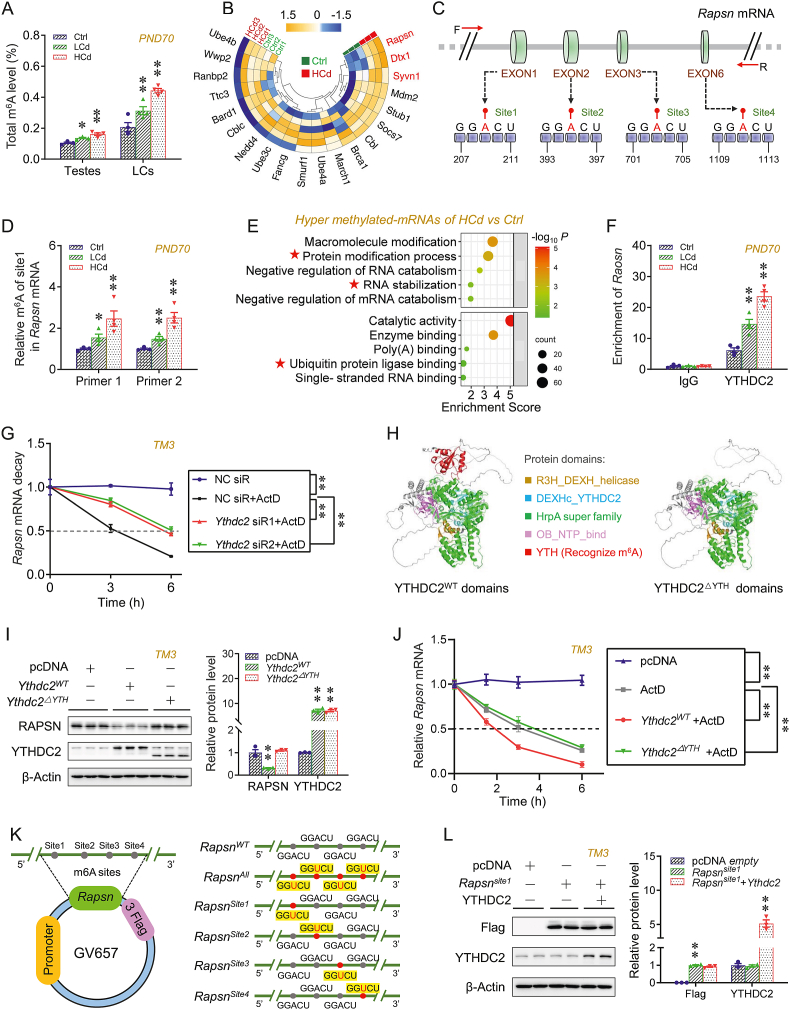
Prenatal Cd stabilizes Rapsn mRNA by reducing YTHDC2 in offspring testes. (A–E) According to Experiment 1, pregnant mice were exposed to different-dose Cd, and F1 males were euthanized for sera and testes collection or mated with untreated WT females to access fertility. (A) Total m^6^A level in F1 testes and primary Leydig cells (n = 4 per group). (B) Heatmap of m^6^A-mRNA epitranscriptomic analysis for potential top 20 E3s for NR4A1 in F1 testes on PND70 (n = 3 per group). (C) Schematic diagram of predicted m^6^A-methylated sites in *Rapsn* mRNA. (D) The m^6^A-methylated levels in *Rapsn* mRNA site 1 in F1 testes on PND70 (n = 4 per group). (E) Most enriched GO terms for m^6^A-methylated mRNAs in epitranscriptome (n = 3 per group). (F) The RIP analysis for YTHDC2 protein and *Rapsn* mRNA in F1 testes (n = 4 per group). (G) *Rapsn* mRNA decay in TM3 cells after being transfected with *Ythdc2* siRNAs (siRs) (n = 3 per group). (H–K) According to Supplementary Cell experiments, the YTHDC2^WT^ or YTHDC2^ΔYTH^-overexpressing TM3 cells were generated. (H) Schematic diagram for the protein domains in YTHDC2^WT^ or YTHDC2^ΔYTH^. (I) YTHDC2 and RAPSN expression in TM3 cells (n = 3 per group). (J) *Rapsn* mRNA decay (n = 3 per group). (K) Schematic diagram of plasmids expressing wild-type *Rapsn* mRNA (*Rapsn*^*WT*^) or *Rapsn* mRNA with m^6^A-methylated sites mutated (*Rapsn*^*MUT*^). (L) Flag and YTHDC2 expression in TM3 cells treated using plasmids expressing *Rapsn*^*site1*^ with or without plasmids expressing *Ythdc2*^WT^ (n = 3 per group). Data were analyzed using Repeated-measures ANOVA (G, J), one-way ANOVA (A, D, F, I, L), and presented as means ± SEM. Numeric data are provided in Table S9. ∗*P* < 0.05, ∗∗*P* < 0.01, compared to Ctrl/NC (negative control) siR/pcDNA-empty group.

m^6^A was an abundant RNA modification regulating the splicing, translation, and decay rate of mRNAs [22]. An enzyme-linked immunosorbent assay (ELISA)-like assay indicated that prenatal Cd exposure resulted in higher total m^6^A modification in F1 testes and primary Leydig cells (Fig. 4A). The higher expression of several m^6^A methyltransferase genes, including *Mettl6*, *Mettl3*, *Wtap,* and *Mettl4*, was also observed in these F1 testes (Fig. S11A and B). m^6^A-mRNA epitranscriptomic analysis revealed that the level of m^6^A modification in *Rapsn* mRNA was the greatest in prenatal Cd-exposed offspring testes among the top 20 E3s (Fig. 4B). Using the SRAMP database, we predicted four potential m^6^A modification sites located in a consensus “RRACH” motif (R = G or A, H = A, C, or U) of *Rapsn* mRNA (Fig. 4C). MeRIP-qPCR analysis revealed that prenatal Cd exposure promoted the m^6^A modification of site 1 in *Rapsn* mRNA in F1 testes (Figs. 4D and S11C-E). GO analysis demonstrated that m^6^A methylation was closely associated with the “protein modification process pathway” and “RNA stabilization pathway” in F1 testes with testosterone deficiency (TD) (Fig. 4E). The fate of m^6^A-modified mRNAs was determined by the recognition of m^6^A readers. The prenatally Cd-exposed group exhibited a lower expression of m^6^A reader YTHDC2 in F1 testes both on PND35 and PND70 (Fig. S12). The RIP analysis indicated that the interactions between the YTHDC2 protein and *Rapsn* mRNA were greater in prenatal Cd-exposed F1 testes (Fig. 4F). YTHDC2-deficient TM3 cells were generated to explore the role of YTHDC2 reduction in RAPSN expression. RAPSN expression was higher in YTHDC2-deficient cells (Fig. S13). The cells were then treated with the RNA transcription inhibitor ActD to determine the stability of *Rapsn* mRNA. The half-life of *Rapsn* mRNA was longer in TM3 cells after YTHDC2 knockdown (Fig. 4G).

The model of the YTHDC2 protein domains was established using the UniProtKB database and visualized using PyMOL software. Five major protein domains were present in the model: R3H_DEXH_helicase, DEXHc_YTHDC2, HrpA superfamily, OB_NTP_bind, and YTH (Fig. 4H). TM3 cells were transfected with plasmids expressing YTHDC2^ΔYTH^ (YTHDC2 deleted YTH domain, the domain for recognizing m^6^A modification in YTHDC2) or with YTHDC2^WT^ (wild-type YTHDC2 protein) (Figs. 4I and S14A). RAPSN expression was lower in YTHDC2^WT^-overexpressing cells but remained the same in YTHDC2^ΔYTH^-overexpressing cells (Fig. 4I). The half-life of *Rapsn* mRNA was shorter in YTHDC2^WT^-overexpressing TM3 cells, whereas YTHDC2^ΔYTH^ overexpression had no such effect (Figs. 4J and S14B). Additionally, the plasmids expressing *Rapsn*^*WT*^ (wild-type *Rapsn* mRNA), *Rapsn*^*All*^ (*Rapsn* mRNA mutated at all potential m^6^A-methylated sites), and *Rapsn*^*SiteN*^ (*Rapsn* mRNA mutated at only the potential m^6^A modification site *N*, *N* = 1, 2, 3, or 4) were synthesized to transfect TM3 cells (Fig. 4K). The reduction in *Rapsn* mRNA in YTHDC2-overexpressing cells was abolished when all m^6^A-methylated sites in *Rapsn* mRNA were mutated (Fig. S15A and B). The same phenotype was also observed when only the m^6^A-methylated site 1 in *Rapsn* mRNA was mutated (Figs. 4L and S15C-E).

### 3.5 Postnatal YTHDC2 overexpression mitigates prenatal Cd-repressed testosterone synthesis in offspring testes

**Fig. 5:**
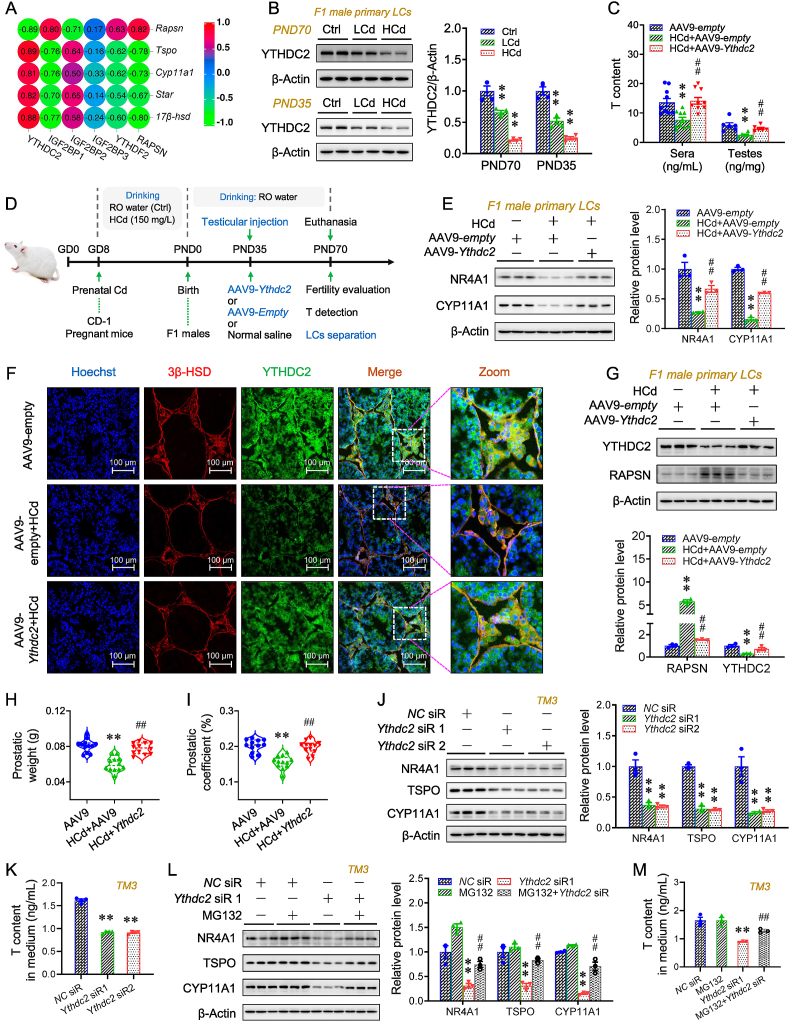
Postnatal YTHDC2 overexpression mitigates prenatal Cd-repressed testosterone synthesis in offspring testes. (A–B) According to Experiment 1, pregnant mice were exposed to different-dose Cd, and F1 males were euthanized for sera and testes collection or mated with untreated WT females to access fertility. (A) Correlation analysis for m^6^A readers with *Rapsn* mRNA and steroidogenic enzymes in F1 testes (n = 4 per group). (B) YTHDC2 expression in the primary Leydig cells from F1 testes on PND35 and PND70 (n = 4 per group). (C–I) According to Experiment 4, pregnant mice were exposed to HCd, and F1 males with testicular YTHDC2 overexpression were generated. (C) T content in sera (n = 10 per group) and testes (n = 6 per group). (D) Experimental schematic. (E) NR4A1 and CYP11A1 expression in the primary Leydig cells (n = 3 per group). (F) Immunofluorescence analysis for 3β-HSD and YTHDC2 in the testes (n = 3 per group). (G) YTHDC2 and RAPSN expression in the primary Leydig cells (n = 3 per group). (H,I) Prostatic weight and coefficient (n = 12 per group). (J,K) The TM3 cells were transfected with *Ythdc2* siRs. (J) NR4A1, TSPO, and CYP11A1 expression in the cells (n = 3 per group). (K) T in the culture medium (n = 3 per group). (L,M) The TM3 cells were transfected with *Ythdc2* siRs with or without MG132. (L) NR4A1, TSPO, and CYP11A1 expression in the cells (n = 3 per group). (M) T in the culture medium (n = 3 per group). Data were analyzed using One-way ANOVA (B–M) and presented as means ± SEM. Spearman correlation was applied (A). Numeric data are provided in Table S10. ∗*P* < 0.05, ∗∗*P* < 0.01, compared to pcDNA/AAV9-empty group; ^#^*P* < 0.05, ^##^*P* < 0.01, compared to HCd/*Ythdc2* siR1 group.

The correlation analysis indicated that offspring T synthesis repression was most strongly associated with the reduction in m^6^A reader YTHDC2 upon prenatal Cd exposure (Fig. 5A). Prenatal Cd exposure led to lower YTHDC2 expression in F1 primary Leydig cells (Figs. 5B and S16). Postnatal testicular YTHDC2-overexpressing mice were generated to explore the effect of YTHDC2 expression on T synthesis in offspring upon prenatal Cd exposure (Fig. 5D). In the YTHDC2 overexpression + HCd group, there was a higher level of T in F1 serum and testes than that in the HCd group (Fig. 5C). Postnatal YTHDC2 overexpression led to higher expression of NR4A1 and steroidogenic enzymes in F1 testes and primary Leydig cells following prenatal Cd exposure (Figs. 5E and S17A). In particular, YTHDC2 overexpression reversed prenatal Cd-induced higher expression of RAPSN and Ub in F1 testes and primary Leydig cells (Figs. 5F,G and S17B,C). Postnatal YTHDC2 overexpression also mitigated prenatal Cd-induced reduction in prostate size in F1 males (Fig. 5H and I). YTHDC2-deficient TM3 cells were then generated to confirm the above conclusion. The lower levels of NR4A1 and steroidogenic enzymes were also observed in YTHDC2-deficient cells (Fig. 5J). YTHDC2 deficiency also induced a lower level of T in the cellular medium (Fig. 5K). Moreover, the phenotype of T synthesis in YTHDC2-deficient TM3 cells was reversed by proteasome inhibitor MG132 (Fig. 5L and M).

### 3.6 Prenatal Cd activates oxidative stress to reduce fetal testicular YTHDC2

Fetal testes were collected to explore the effect of prenatal Cd exposure on YTHDC2 expression in the fetal stage. The prenatally Cd-exposed group exhibited higher expression of RAPSN and Ub, and lower expression of YTHDC2 in fetal testes and Leydig cells (Figs. S18A-C and S19). The lower levels of NR4A1, steroidogenic enzymes, and T content also occurred in the fetal males with prenatal Cd exposure (Fig. S18D and E). Moreover, prenatal Cd caused higher oxidative stress in fetal testes, as indicated by the elevations in NOX4, SOD2, and TRX1, three biomarkers of oxidative stress (Fig. S18F). TM3 cells were treated with H_2_O_2_ to explore the effect of oxidative stress on YTHDC2 expression in Leydig cells. There was a lower level of YTHDC2 in H_2_O_2_-treated TM3 cells (Fig. S20A). In contrast, higher levels of RAPSN and Ub occurred in these cells (Fig. S20A). Lower expression of NR4A1 and steroidogenic enzymes in H_2_O_2_-treated TM3 cells, along with reduced T levels in the culture medium, was observed (Fig. S20A–D). Most importantly, the lower expressions of NR4A1 and steroidogenic enzymes in H_2_O_2_-treated TM3 cells were reversed by the proteasome inhibitor MG132 (Fig. S20E and F). The pregnant mice were treated with the antioxidant NAC, and the expression of YTHDC2 in fetal testes was then determined upon prenatal Cd exposure. There were higher levels of maternal weight, fetal weight, and fetal crown-rump length in prenatally Cd-exposed mice with NAC supplementation (Fig. S21A–C). The placental size remained the same in the different groups (Fig. S21D and E). Prenatal NAC supplementation also persistently reversed Cd-induced reduction in male offspring weight (Fig. S21F). In the NAC + HCd group, there were higher expressions of YTHDC2, NR4A1, and steroidogenic enzymes in fetal testes relative to the HCd group (Figs. S18H and S22A). The higher levels of T were also observed in prenatal Cd-exposed fetal sera after NAC treatment (Fig. S18G). Prenatal NAC supplementation led to reduced expressions of RAPSN and Ub in Cd-exposed fetal testes (Fig. S18H and I). The levels of NOX4, SOD2, and TRX1 were lower in prenatal Cd-exposed fetal testes after NAC supplementation (Fig. S22B).

### 3.7 Prenatal YTHDC2 elevation alleviates Cd-impaired testosterone synthesis and fertility in F1 males

The effects of fetal YTHDC2 expression on prenatal Cd-induced T synthesis inhibition and subfertility in adolescent and adult offspring were then investigated (Fig. S23A). In detail, 15 F1 males from different litters in each group were mated with untreated WT females at a ratio of 1:2. Fetal testicular YTHDC2 elevation induced by NAC led to a lower rate of F1 males failing to produce offspring in the Cd group (Fig. S23B). The NAC + HCd group also exhibited a higher number of F2 offspring compared to the HCd group (Fig. S23C). Fetal testicular YTHDC2 elevation induced by NAC also mitigated prenatal Cd-induced reductions in the rate of F1 male first mating and the rate of female pregnancy (Figs. S23D,E and S24A,B). Moreover, fetal testicular YTHDC2 elevation promoted testicular descent and reversed AGD and AGI reduction in offspring with prenatal Cd exposure (Figs. S23F-H and S24C). Fetal testicular YTHDC2 elevation also alleviated Cd-induced reduction in prostate size in offspring prenatally exposed to Cd (Fig. S24D and E). The effects of prenatal testicular YTHDC2 elevation on T synthesis in offspring with prenatal Cd exposure were subsequently determined. Compared to the HCd group, the level of T in the NAC + HCd group was higher in F1 sera and testes (Fig. S23I and J). Correspondingly, the levels of steroidogenic enzymes, such as TSPO, CYP11A1, StAR, and 17β-HSD, were also higher in these F1 testes (Figs. S23K, M and S24F,G). NR4A1 expression was also persistently upregulated by prenatal testicular YTHDC2 elevation (Fig. S23L and N). The lower YTHDC2 expression and the higher RAPSN expression in F1 testes prenatally exposed to Cd were observed after fetal YTHDC2 elevation (Fig. S23L and N). Furthermore, prenatal YTHDC2 elevation induced lower ubiquitin expression in the testes of F1 offspring with prenatal Cd exposure (Fig. S24H).

### 3.8 Prenatal YTHDC2 elevation alleviates Cd-impaired testosterone synthesis and fertility in F2 and F3 males

**Fig. 6:**
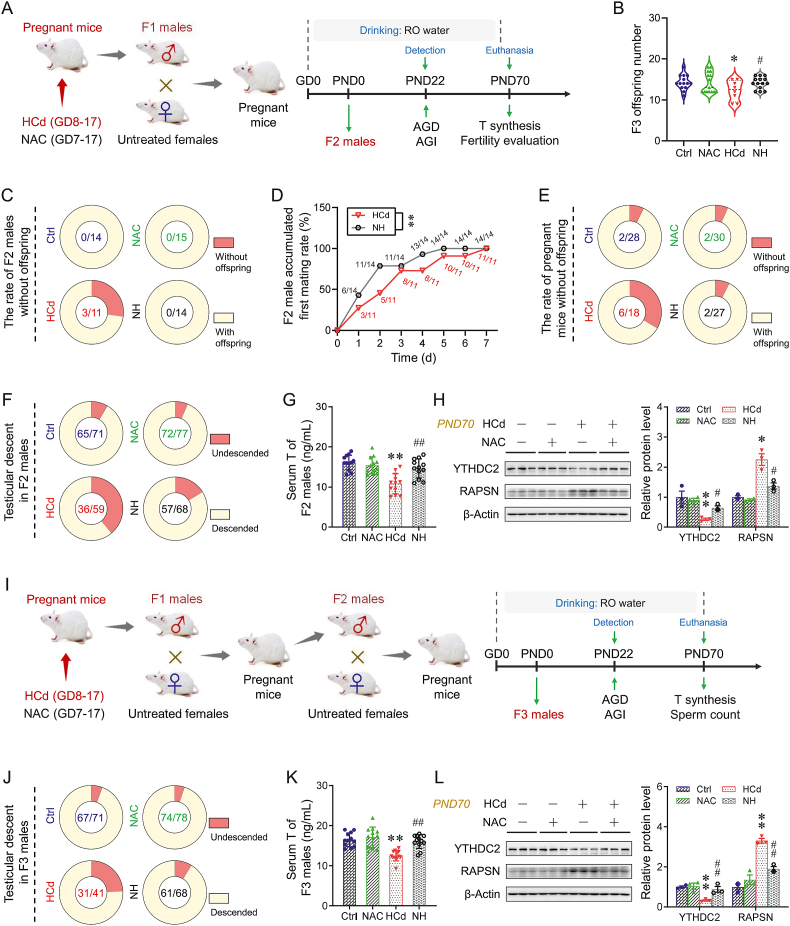
Prenatal YTHDC2 elevation alleviates Cd-impaired testosterone synthesis and fertility in F2 and F3 males. (A–H) According to Experiment 5, F1 males were mated with untreated WT females to generate F2 offspring, and F2 males were euthanized for sera and testes collection. (A) Envrionmental schematic. (B) The number of F3 offspring. (C) The rate of F2 males without offspring. (D) Cumulative first-mating rate of F2 males within seven days (n = 11 in HCd, n = 14 in NH). (E) The rate of pregnant mice without offspring. (F) Testicular descent rate in F2 males. (G) T levels in male F2 sera (n = 12 per group). (H) YTHDC2 and RAPSN expression in F2 testes on PND70 (n = 3 per group). (I–L) According to Experiment 5, F2 males were mated with untreated WT females to generate F3 offspring, and F3 males were euthanized for sera and testes collection. (I) Envrionmental schematic. (J) Testicular descent rate in F3 male. (K) T levels in male F3 sera (n = 12 per group). (L) YTHDC2 and RAPSN expression in F3 testes on PND70 (n = 3 per group). Data were analyzed using repeated-measures ANOVA (D), one-way ANOVA (B, G, K, and L), and presented as means ± SEM. Numeric data are provided in Table S13. ∗*P* < 0.05, ∗∗*P* < 0.01, compared to Ctrl; ^#^*P* < 0.05, ^##^*P* < 0.01, compared to HCd group.

F1 males from different litters in each group were mated with untreated WT females to produce F2 offspring (Fig. 6A). F2 males from different litters in each group were mated with untreated WT females at a ratio of 1:2. Notably, F1 fetal testicular YTHDC2 elevation induced by NAC decreased the rate of prenatal Cd-evoked infertility in F2 males (Fig. 6C). The prenatal Cd-induced lower numbers in F3 offspring were also alleviated by F1 fetal testicular YTHDC2 elevation (Fig. 6B). There was a higher level of sperm count in the HCd group after F1 fetal testicular YTHDC2 elevation (Fig. S25A). Prenatal YTHDC2 elevation mitigated Cd-induced reduction in the cumulative first-mating rate in F2 males and the cumulative pregnancy rate in females (Figs. 6D,E, and S25B-D). F1 fetal testicular YTHDC2 elevation also alleviated prenatal Cd-induced delay in testicular descent in F2 males (Figs. 6F and S25E). The prenatal Cd-induced lower levels of AGD and AGI in F2 males were also mitigated by F1 fetal testicular YTHDC2 elevation (Fig. S25F and G). Moreover, the levels of steroidogenic enzymes in these F2 testes and the T content in the sera were also reversed (Figs. 6G and S25H). F2 testicular NR4A1 expression in the NAC + HCd group was also higher than that in the HCd group (Fig. S25H). Prenatal YTHDC2 elevation persistently reversed Cd-induced lower expression in YTHDC2, downregulating the levels of RAPSN and Ub in F2 testes (Figs. 6H and S25H).

F2 males from different litters in each group were mated with untreated WT females at a ratio of 1:2 to produce F3 offspring (Fig. 6I). Sperm counts were higher in the HCd group after F1 fetal testicular YTHDC2 elevation (Fig. S26A). F1 fetal testicular YTHDC2 elevation reduced prenatal Cd-induced delay in testicular descent in F3 offspring (Figs. 6J and S26B). The prenatal Cd-induced lower levels of AGD and AGI in F3 males were also mitigated (Fig. S26C and D). Similarly, the levels of steroidogenic enzymes in these F3 testes and the T content in the sera were also higher (Figs. 6K and S26E). F3 testicular NR4A1 expression in the NAC + HCd group was also higher than that in the HCd group (Fig. S26E). Prenatal YTHDC2 elevation persistently reversed Cd-induced lower expression in YTHDC2, downregulating the levels of RAPSN and Ub in F3 testes (Fig. 6L). Prenatal Cd also increased DNA methylation levels in both the testes and sperm in multi-generational offspring (Fig. S27A and B). Furthermore, using the MethPrimer database, we found a CpG island near the YTHDC2 promoter’s transcription start site (Fig. S27C).

## 4 Discussion

Infertility affects approximately 17% of the global population [1]. TD, the main cause of male infertility, affects approximately 35% of infertile couples worldwide [53]. Several studies have demonstrated that adult male TD could originate from early-life exposure to adverse factors and develop throughout life [[54], [55], [56]]. However, the multi-generational inheritance characteristics and the mechanism of TD-dependent male subfertility are poorly understood. Cd is a classical environmental toxicant with developmental toxicity [49,57]. An animal study indicated that Cd exposure decreased multi-generational fertility in the model organism *Drosophila* [15]. Herein, prenatal Cd exposure also induced multi-generational male subfertility, demonstrated by the lower offspring number and the higher infertility rate in male F1-F3 mice. A population-based study revealed a close relationship between later-life T reduction and other prenatal environmental toxicant exposure, including parabens [58]. Global transcriptome revealed that downregulated testicular genes were enriched mainly in the “sterol biosynthetic process pathway” and “response to T pathway” in the offspring with prenatal Cd exposure. We confirmed that prenatal Cd repressed T synthesis, and then decreased the T content in the sera and testes over several generations. Two animal studies have also shown that other prenatal stressors, such as dexamethasone [59] and polybrominated diphenyl ethers [56], induce TD by blocking steroidogenesis in offspring testes. We concluded that prenatal Cd elevated multi-generational susceptibility to male subfertility by suppressing T synthesis in offspring testes.

NR4A1 [60] and NR5A1 [45], two orphan nuclear receptors, were known cotranscriptional factors regulating steroidogenic enzymes in Leydig cells. Herein, the mRNA levels of testicular steroidogenic enzymes, including *Tspo*, *Star*, *Cyp11a1,* and *17β-hsd,* were lower in offspring with prenatal Cd exposure, suggesting that transcription was repressed. Furthermore, NR4A1 expression was persistently downregulated in these testes, whereas NR5A1 expression was upregulated. Prenatal Cd exposure also reduced the expression of NR4A1 in offspring primary Leydig cells. A cellular experiment confirmed that NR4A1 overexpression promoted the expression of steroidogenic enzymes, including StAR, CYP11A1, CYP17A1, and 3β-HSD, whereas NR4A1 knockdown significantly reduced the expression of these steroidogenic enzymes [61]. Therefore, the NR4A1-specific reduction mediated prenatal Cd-induced TD by suppressing T synthesis in offspring testes.

Protein homeostasis is balanced via gene transcription [51], mRNA translation [51], and protein degradation [52]. The *Nr4a1* mRNA and p-eIF2α protein levels were unchanged in F1 testes from different groups, indicating that *Nr4a1* transcription and translation were the same. The protein degradation pathways included autophagy and UPS [62]. However, the level of testicular LC3B-II, an autophagosome marker, was decreased in offspring with prenatal Cd exposure, suggesting that NR4A1-specific degradation was not due to autophagy. Global transcriptome revealed that testicular upregulated genes were enriched mainly in the “protein modification process pathway” and “ubiquitin-like protein transferase activity” in offspring with prenatal Cd exposure. NR4A1-specific ubiquitylation was confirmed to be enhanced in offspring testes and primary Leydig cells upon prenatal Cd exposure. Furthermore, postnatal testicular MG132 (a proteasome inhibitor) injection reversed prenatal Cd-induced Ub-dependent degradation in NR4A and subsequent T-synthesis suppression in offspring testes. A cellular experiment indicated that NR4A1 homeostasis was regulated by posttranslational ubiquitination and degradation by the UPS in HEK293T cells [63]. In summary, prenatal Cd induced NR4A1-specific degradation by enhancing Ub modification in offspring testes.

Ubiquitination was the process of covalent binding of Ub to target proteins catalyzed by various enzymes, including ubiquitin-activating enzymes (E1s), E2s, and E3s [64]. The specificity of E3s for protein substrates regulated the targeting of ubiquitination modification [65]. Herein, RAPSN was identified as the most suitable E3 ligase for NR4A1 in the offspring testes after prenatal Cd exposure. RAPSN was previously identified as an E3 ubiquitin ligase in muscle cells [66]. However, the function of RAPSN in the testes was unclear. RAPSN expression was persistently upregulated in offspring testes and primary Leydig cells following prenatal Cd. According to the docked RAPSN-NR4A1 model, the protein atomic structures of RAPSN and NR4A1 were closely related. The interaction between RAPSN and NR4A1 was confirmed to be higher in offspring testes after prenatal Cd. Testicular RAPSN overexpression induced the higher ubiquitin expression and the lower NR4A1 expression in testes and primary Leydig cells. There are three families of enzymes with E3 activity: E3s with HECT domains [67], RING finger domains [67], and U-box domains [68]. We discovered that RAPSN has a RING finger domain named RING-H2_Rapsyn. A recent cellular study also revealed that the RING domain of RAPSN in HEK293T cells exhibited E3 ligase activity [69]. Importantly, RAPSN overexpression-induced NR4A1 degradation was repressed when the RING-H2_Rapsyn domain was mutated in TM3 cells. In summary, RAPSN-mediated ubiquitin-dependent degradation of NR4A1 was a potential mechanism of TD in offspring testes upon prenatal Cd exposure.

m^6^A, one of the most prevalent RNA-methylated modifications in eukaryotic cells, was involved in the occurrence and development of numerous diseases by regulating RNA splicing, translation, and degradation [70]. m^6^A modification levels and the expression of m^6^A writers, including METTL16, WTAP, and METTL14, were higher in offspring testes with prenatal Cd exposure. A population-based study revealed that m^6^A modification is significantly enhanced in primary Leydig cells isolated from subfertile patients with TD [27]. Among the top 20 potential E3s for NR4A1, the *Rapsn* mRNA m^6^A modification was the highest in offspring testes with prenatal Cd exposure according to MeRIP-seq analysis, SRAMP database prediction, and MeRIP-qPCR analysis. Furthermore, m^6^A methylation was closely related to the “RNA stabilization pathway”, and the *Rapsn* mRNA level was increased in these testes. Several studies have confirmed that m^6^A modification regulates the stability of m^6^A-methylated mRNAs [[71], [72], [73]]. In summary, enhanced m^6^A modification increased *Rapsn* mRNA stability in the testes of offspring with prenatal Cd exposure.

The fate of m^6^A-modified mRNAs, including splicing, translation, and decay, was determined by the ability of the readers to recognize and bind m^6^A modifications [74,75]. m^6^A readers, including YTHDC2 and YTHDF2, promoted the degradation of m^6^A-modified mRNAs. In contrast, IGF2BPs enhanced the stability of m^6^A-modified mRNAs. Notably, correlation analysis revealed that higher *Rapsn* mRNA levels were closely associated with lower YTHDC2 expression in the testes of subfertile offspring. YTHDC2 was highly expressed in the testes and regulates meiosis and spermatogenesis [29]. However, the role of YTHDC2 in regulating T synthesis of Leydig cells remained unclear. In vivo testicular YTHDC2 overexpression and in vitro YTHDC2 knockdown confirmed that prenatal Cd exposure reduced YTHDC2 to drive TD-dependent subfertility in male offspring. Furthermore, YTHDC2 knockdown increased *Rapsn* mRNA stability and Ub-dependent degradation of NR4A1 in subfertile offspring testes. A previous GO analysis revealed that YTHDC2-bound mRNAs were closely associated with the ubiquitination pathway [76]. Using the UniProt database, YTHDC2 was observed to have numerous protein domains, including YTH, R3H, and OB domains. The YTH domain in YTHDC2 was able to bind to m^6^A modifications in mouse embryonic fibroblasts [77]. Herein, YTHDC2-induced *Rapsn* mRNA degradation was repressed when the m^6^A methylation site was mutated in the mRNA. *Rapsn* mRNA stability was also higher after deletion of the m6A-recognizing YTH domain from the YTHDC2 protein. In conclusion, the reduction in YTHDC2 mediated prenatal Cd-induced upregulation of RAPSN in offspring testes.

Developmental changes in early life stages affect the adult reproductive phenotype [78,79]. These changes, including YTHDC2 reduction-induced increases in *Rapsn* mRNA stability and subsequent TD, were observed during the fetal stage. However, the mechanism of the reduction in testicular YTHDC2 expression in the fetal stage was not yet clear. Oxidative stress, characterized by excess reactive oxygen species (ROS) due to an imbalance between production and elimination [80], was the primary cause of parental male subfertility [81]. Several studies reported that ROS overexposure in the fetal stage induced cardiovascular dysfunction in adulthood [[82], [83], [84]]. Oxidative stress was higher in fetal testes following prenatal Cd exposure. ROS overexposure disrupted the homeostasis of m^6^A modifications in eye tissues [85]. ROS overexposure promoted YTHDC2 reduction and increased *Rapsn* mRNA stability to induce TD in Leydig cells. NAC, a well-known antioxidant, has been used as an effective and safe therapeutic option for parental male subfertility [86]. In this study, Cd-induced reduction in YTHDC2 in fetal testes was effectively repressed by prenatal NAC supplementation. In particular, prenatal YTHDC2 elevation effectively reversed Cd-induced male offspring subfertility with TD by promoting the degradation of m^6^A-methylated *Rapsn* mRNA. Therefore, prenatal Cd-induced male subfertility originated from a reduction in testicular YTHDC2 during the fetal stage.

The DOHaD theory states that early-life developmental alterations not only affect the disease phenotype in later life but also increase transgenerational susceptibility to disease [78,87]. In this study, prenatal Cd exposure induced TD-dependent male subfertility in F1−F3 offspring. Previous studies have indicated that classic epigenetic modifications, including DNA methylation, small noncoding RNA, and histone modification, are essential for the transgenerational inheritance of various diseases [[88], [89], [90]]. However, m^6^A modification has not been well studied. A recent study demonstrated that m^6^A variation was closely related to genetic and phenotypic variation in human disease inheritance [20]. Herein, the reduction in the m^6^A reader YTHDC2 in F1 testes with TD was transmitted to F2 and F3 offspring testes with TD. Moreover, RAPSN-mediated ubiquitination and subsequent NR4A1 degradation were also higher in F2 and F3 testes. Prenatal testicular YTHDC2 elevation not only mitigated Cd-induced TD-dependent male subfertility in F1 offspring but also reduced transgenerational susceptibility to this disease in F2 and F3 offspring by promoting m^6^A-modified *Rapsn* mRNA decay. In conclusion, the reduction in YTHDC2 mediated prenatal Cd-induced multi-generational and transgenerational susceptibility to TD-dependent male subfertility.

In this study, we established a multi-generational mouse model of male subfertility by simulating maternal serum Cd concentration in humans. Based on this model, multi-generational males with subfertility exhibited testicular T synthesis repression and TD. Global transcriptome and m^6^A epitranscriptome suggested that dysregulated m^6^A modification of *Rapsn* mRNA enhanced ubiquitination, increasing multi-generational and transgenerational susceptibility to TD upon prenatal Cd exposure. Testicular MG132 injection, together with overexpression of RAPSN and YTHDC2, demonstrated that reduced m^6^A reader YTHDC2 improved *Rapsn* mRNA stability, repressing testicular T synthesis in multi-generational offspring. These multi-generational and transgenerational changes originated from dysregulated m^6^A modification in F1 fetal testes. Detailed information is shown in the schematic diagram of the conclusion (Fig. S28).

In summary, the multi-generational mouse model of male subfertility provides direct evidence supporting epidemiological studies linking maternal environmental stress exposure to reproductive dysfunction in male offspring. Reduced YTHDC2 is closely associated with human male infertility, and previous studies have mainly focused on its effects on spermatogenesis. In this study, YTHDC2 downregulation inhibited T synthesis to impair the fertility of male offspring prenatally exposed to Cd, suggesting reduced YTHDC2 could serve as a potential biomarker for TD. Furthermore, targeting the YTHDC2–RAPSN–NR4A1 axis or modulating m^6^A modifications may provide potential therapeutic avenues to mitigate TD-dependent male subfertility. Together, our findings suggest that dysregulated m^6^A modification can mediate prenatal Cd-elevated multi-generational susceptibility to male subfertility, providing a new insight into environmental toxicant-induced multi-generational toxicity. The limitations of this study are as follows. Firstly, species differences and the complexity of human exposures limit the direct extrapolation of this mouse model to human reproductive toxicology. Secondly, this study simulated the internal exposure dose of Cd in pregnant women, but the external exposure doses in mice during their relatively short gestation period may not fully reflect the real-world conditions in humans. Future studies need to explore the effects and mechanisms of low-dose or environmentally relevant Cd exposure on offspring fertility. Lastly, we didn’t perform rescue experiments using RAPSN knockdown in F1 males to directly demonstrate causality.

## CRediT authorship contribution statement

**Hualong Zhu:** Writing – original draft, Visualization, Project administration, Conceptualization. **Yongwei Xiong:** Data curation. **Zhi Yuan:** Formal analysis. **Yexin Luo:** Investigation. **Kongwen Ouyang:** Methodology. **Tiantian Wang:** Methodology. **Hua Wang:** Methodology. **Yufeng Zhang:** Methodology. **Wei Chang:** Validation. **Jin Zhang:** Visualization. **Hao Li:** Validation. **Lan Gao:** Supervision. **Dexiang Xu:** Supervision. **Hua Wang:** Writing – review & editing, Supervision, Conceptualization.

## Declaration of competing interest

Authors declare there is no competing financial interests in relation to the work described.
