# Supplementary material for: Prenatal cadmium exposure and male infertility in mice: A multi-generational mechanistic study
Source: Eco Environ Health. 2026 Jan 30;5(1):100217. doi: 10.1016/j.eehl.2026.100217 (PMC12914185; doi:10.1016/j.eehl.2026.100217)
Supplement: Multimedia component 1 [file mmc1.docx]

**Supplementary Materials**

**Prenatal cadmium exposure and male infertility in mice: a** **multi-generational mechanistic study**

Hualong Zhu^a,b,c,1^, Yongwei Xiong^a,b,c,1^, Zhi Yuan^a,b,c,1^, Yexin Luo^a,b^, Kongwen Ouyang^a,b^, Tiantian Wang^a,b^, Hua Wang^a,d^, Yufeng Zhang^a,b^, Wei Chang^a,b^, Jin Zhang^a,b^, Hao Li^a,b^, Lan Gao^a,b,c^, Dexiang Xu^a,b,c^, Hua Wang^a,b,c,*^

***Corresponding author.**

Email: [wanghuadev@ahmu.edu.cn](mailto:wanghuadev@ahmu.edu.cn) (H. Wang)

Figures. S1-S28

Figure legends for Figures. S1-S28

Supplementary Materials and Methods

**Supplementary Figures and Figure legends**


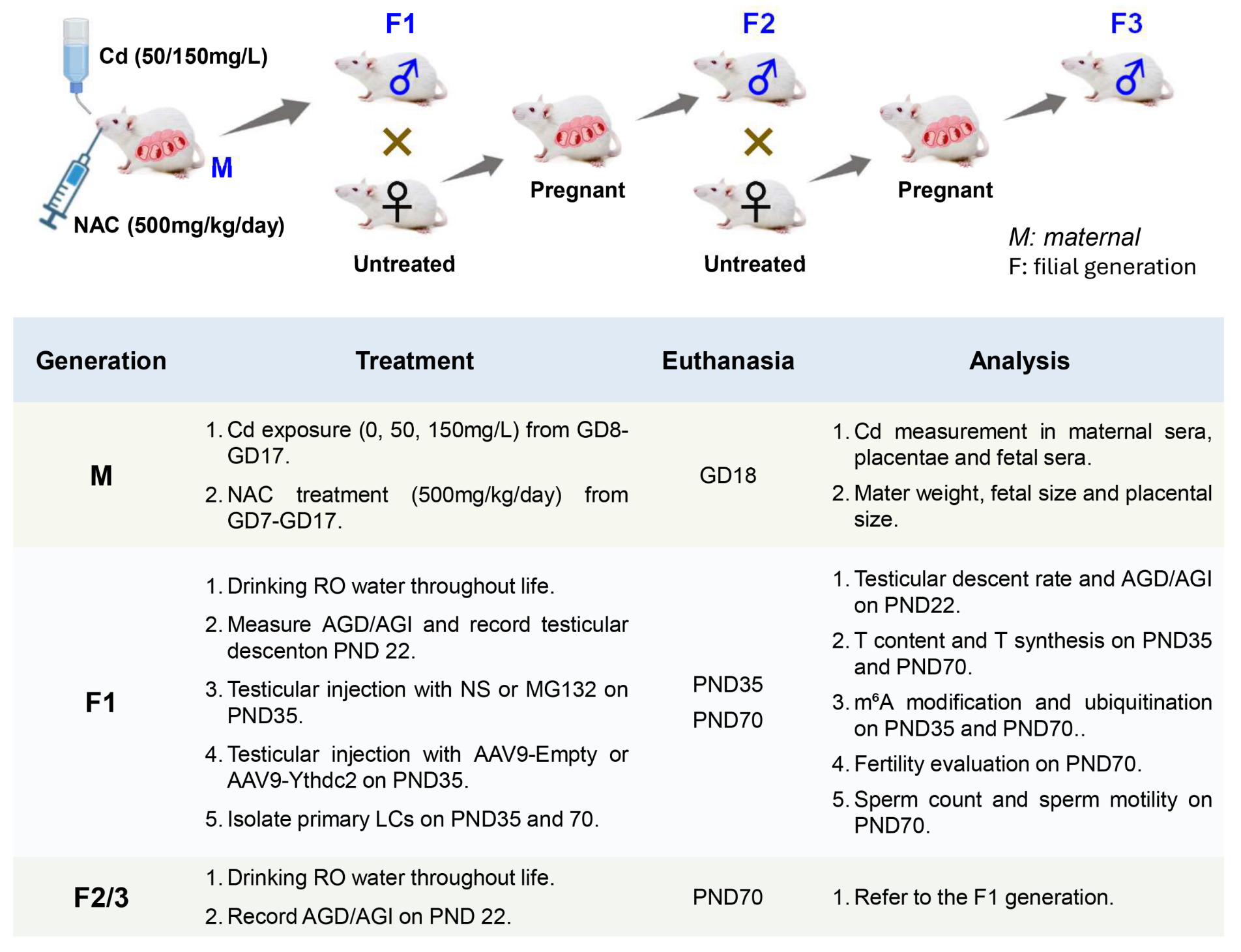


**Fig. S1. The procedures of multi-generational experiments.** The figure provides an overview of the multi-generational experiment, including the breeding strategy and the timeline of assessments.


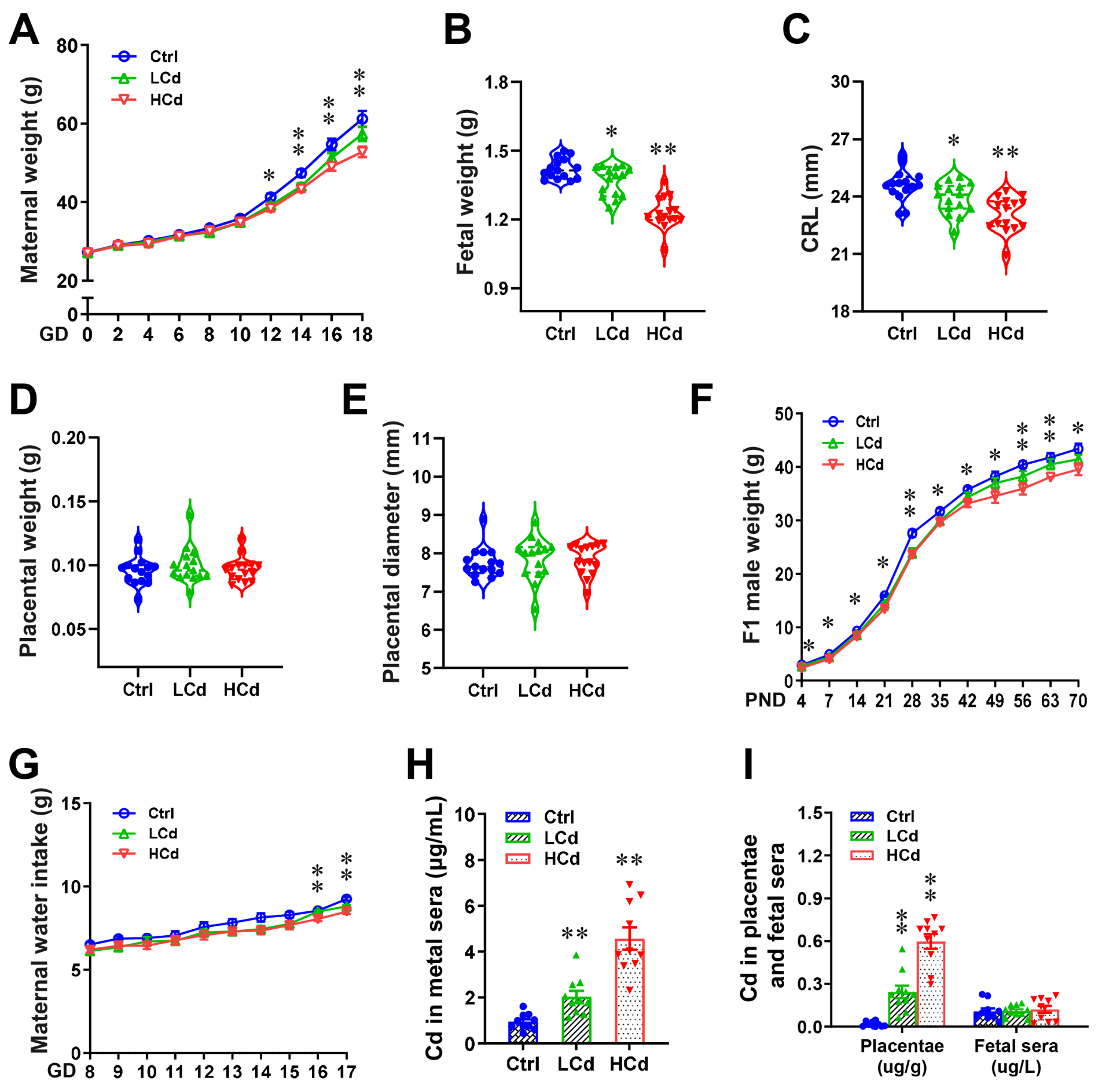


**Fig. S2. The effect of prenatal Cd exposure on fetal growth.** Pregnant mice (n = 15 per group) were treated with LCd (50 mg/L) or HCd (150 mg/L) from GD8 to GD17 in drinking water. All pregnant mice were euthanized on GD18. Maternal sera, placentae and fetal sera were collected. (A) Maternal weight (n = 15 per group). (B-C) Fetal weight and crown-rump length (n = 15 per group). (D-E) Placental weight and diameter (n = 15 per group). (F) Male offspring weight (n = 15 per group). (G) Maternal water intake (n = 6 per group). (H) The level of Cd in maternal sera (n = 10 per group). (I) The level of Cd in placentae (n = 10 per group) and fetal sera (n = 10 per group). All data were analyzed using One-way *ANOVA* and presented as *means* ± *SEM*. Numeric data are provided in Excel Table S14. **P*＜0.05, ***P*＜0.01, compared to Ctrl.


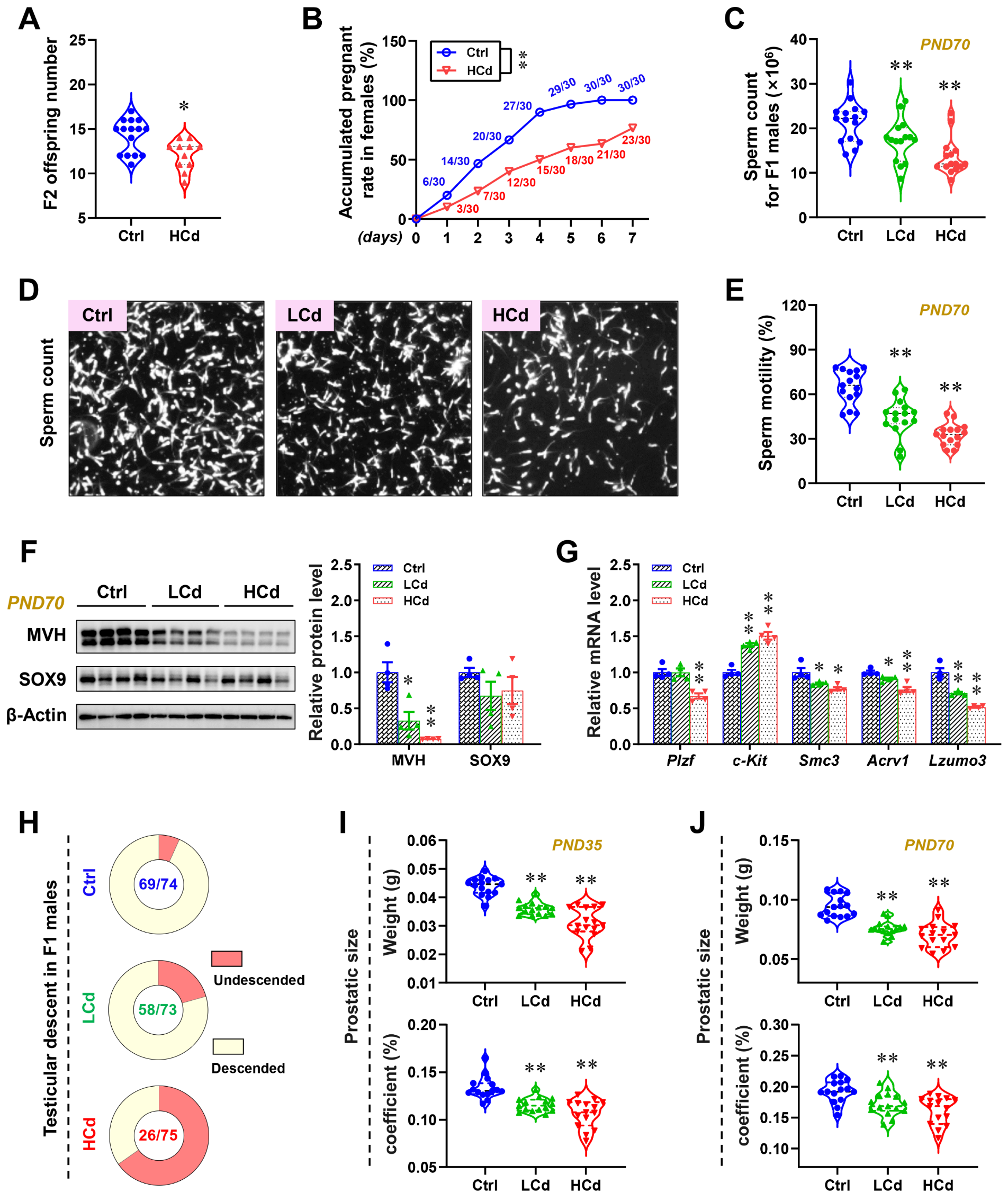


**Fig. S3. The effect of prenatal Cd exposure on male subfertility in F1 offspring.** Pregnant mice (n = 15 per group) were treated with LCd (50 mg/L) or HCd (150 mg/L) from GD8 to GD17 in drinking water and gave birth to F1 offspring on GD18. Some of F1 males were euthanized on PND35 and PND70, respectively. Some of F1 males were mated with WT and untreated females at a ratio of 1:2. (A) F2 offspring number (n = 15 per group). (B) Accumulated pregnancy rate in females mating with F1 males (n = 30 per group). (C) Sperm count for F1 males on PND70 (n = 15 per group). (D) Representative images of sperm count (n = 15 per group). (E) Sperm motility for F1 males on PND70 (n = 15 per group). (F) MVH and SOX9 expression in F1 testes on PND70 (n = 4 per group). (G) Relative *Plzf, c-Kit, Smc3, Acrv1* and *Lzumo3* mRNA levels in PND70 testes (n = 4 per group). (H) Testicular descend in F1 males on PND22 (n = 15 per group). (I-J) Prostatic weight and coefficient in F1 males (n = 15 per group). All data were presented as *means* ± *SEM*. Independent sample *t*-test was applied for A. One-way *ANOVA* was applied for B-C and E-F. Numeric data are provided in Excel Table S15. **P*＜0.05, ***P*＜0.01, compared to Ctrl.


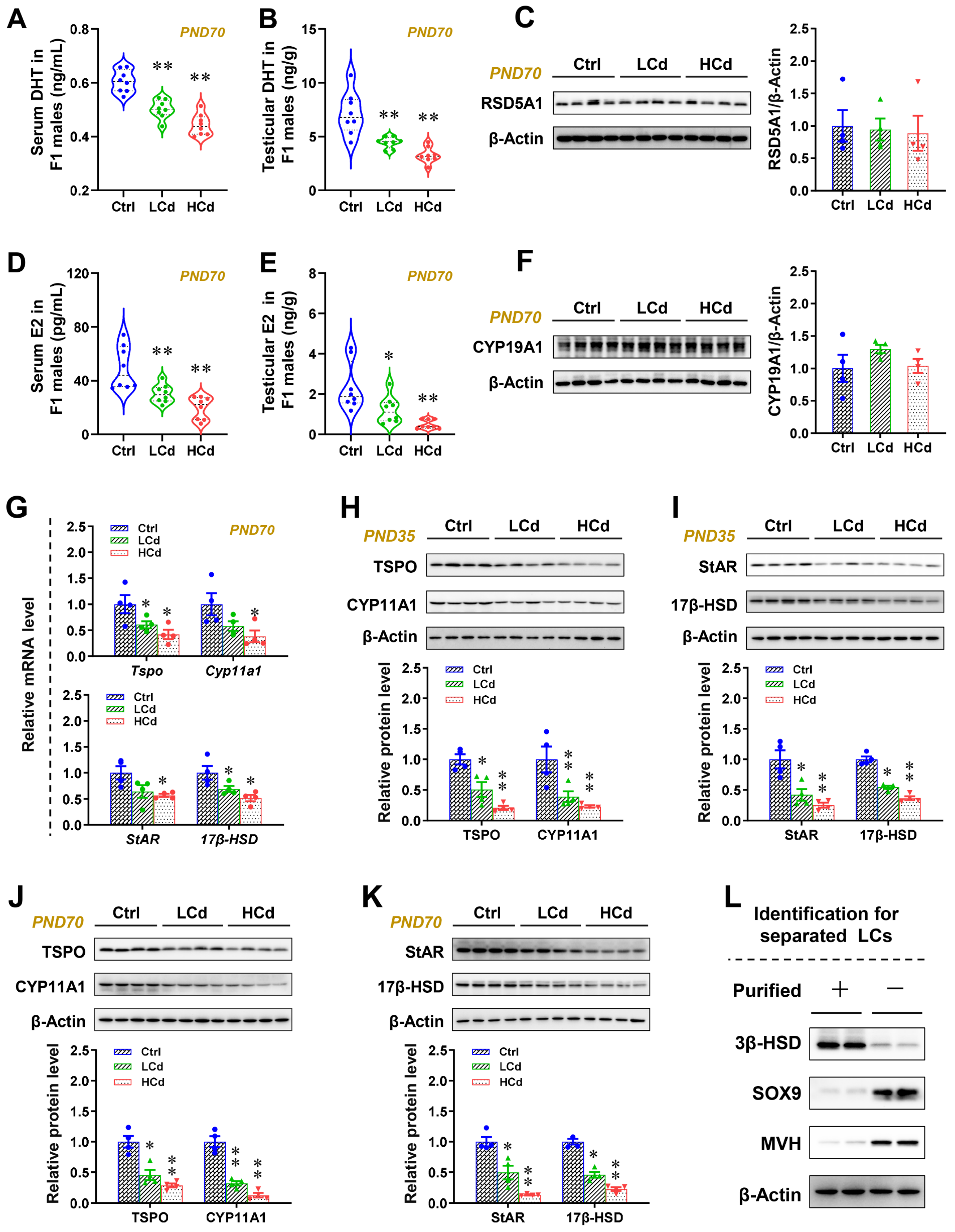


**Fig. S4. The effect of prenatal Cd exposure on testicular testosterone synthesis in F1 males.** Pregnant mice (n = 15 per group) were treated with LCd (50 mg/L) or HCd (150 mg/L) from GD8 to GD17 in drinking water and gave birth to F1 offspring on GD18. Some of F1 males were euthanized on PND35 and PND70, respectively. The F1 testes were collected. (A) The level of DHT in F1 sera PND70 (n = 8 per group). (B) The level of DHT in F1 testes on PND70 (n = 8 per group). (C) RSD5A1 expression in F1 testes on PND70 (n = 4 per group). (D) The level of E2 in F1 sera PND70 (n = 8 per group). (E) The level of E2 in F1 testes on PND70 (n = 8 per group). (F) CYP19A1 expression in F1 testes on PND70 (n = 4 per group). (G) Relative *Tspo*, *Cyp11a1, StAR* and *17β-HSD* mRNA levels (n = 4 per group). (H-K) TSPO, CYP11A1, StAR and 17β-HSD expression in F1 testes on PND35 and PND70 (n = 4 per group). (L) The primary Leydig cells were separated from F1 testes in each group. After the separation, the cells were used to directly to extract proteins, and then preformed immunoblotting analysis. 3β-HSD, SOX9 and MVH expression in the primary Leydig cells (n = 4 per group). All data were analyzed using One-way *ANOVA* and presented as *mean* ± *SEM*. Numeric data are provided in Excel Table S16. **P*＜0.05, ***P*＜0.01, compared to Ctrl.


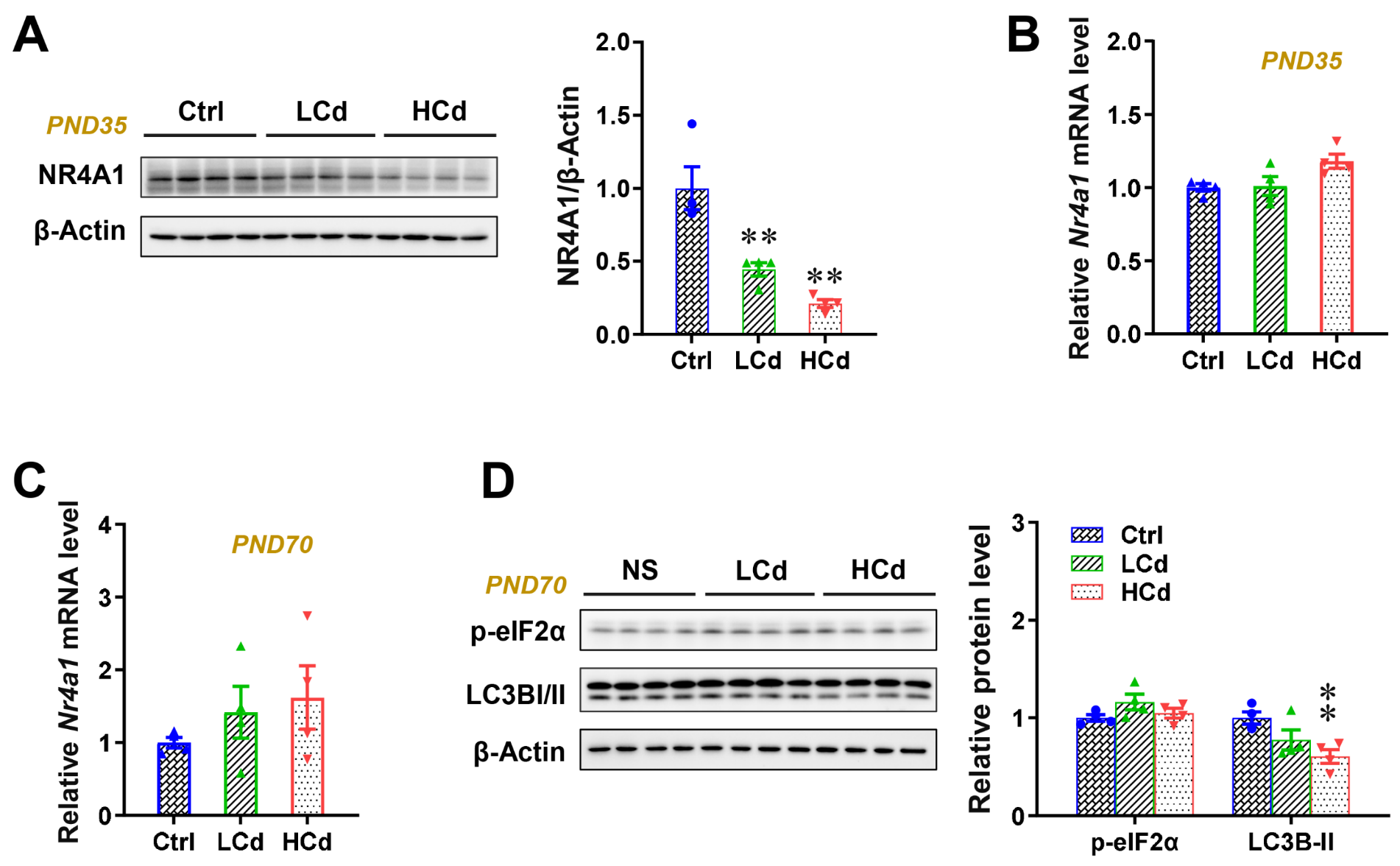


**Fig. S5. The effect of prenatal Cd exposure on NR4A1 expression in F1 testes.** Pregnant mice (n = 15 per group) were treated with LCd (50 mg/L) or HCd (150 mg/L) from GD8 to GD17 in drinking water and gave birth to F1 offspring on GD18. Some of F1 males were euthanized on PND35 and PND70, respectively. The F1 testes were collected. (A) NR4A1 expression in PND35 testes (n = 4 per group). (B) Relative *Nr4a1* mRNA level in PND35 testes (n = 4 per group). (C) Relative *Nr4a1* mRNA level in PND70 testes (n = 4 per group). (D) p-eIF2α and LC3B Ⅰ/Ⅱ expression in PND70 testes (n = 4 per group). All data were analyzed using One-way *ANOVA* and presented as *mean* ± *SEM*. Numeric data are provided in Excel Table S17. **P*＜0.05, ***P*＜0.01, compared to Ctrl.


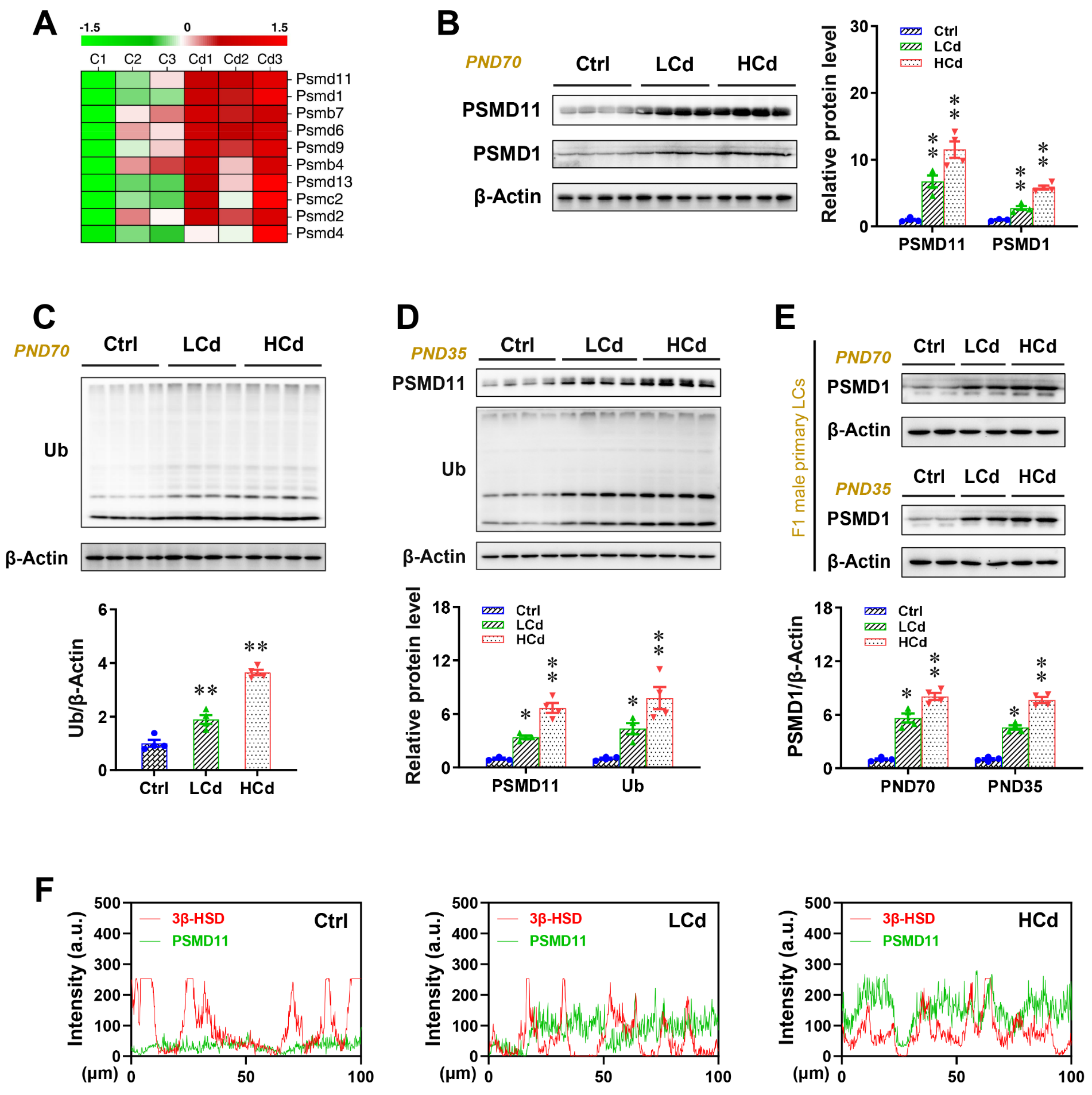


**Fig. S6. The effect of prenatal Cd exposure on ubiquitin expression in F1 testes.** Pregnant mice (n = 15 per group) were treated with LCd (50 mg/L) or HCd (150 mg/L) from GD8 to GD17 in drinking water and gave birth to F1 offspring on GD18. Some of F1 males were euthanized on PND35 and PND70, respectively. The F1 testes were collected. (A) Global transcriptome analysis heatmap for proteasome marker in F1 testes on PND70 (n = 3 per group). (B-C) PSMD11, PSMD1 and Ub expressions in PND70 testes (n = 4 per group). (D) PSMD11 and Ub expressions in PND35 testes (n = 4 per group). (E) The primary Leydig cells were separated from F1 testes in each group. After the separation, the cells were used to directly to extract proteins, and then preformed immunoblotting analysis. PSMD1 expression in the primary Leydig cells (n = 4 per group). (F) Immunofluorescent quantification of 3β-HSD and PSMD11 in F1 testes on PND35 (n = 4 per group). All data were analyzed using One-way *ANOVA* and presented as *mean* ± *SEM*. Numeric data are provided in Excel Table S18. **P*＜0.05, ***P*＜0.01, compared to Ctrl.


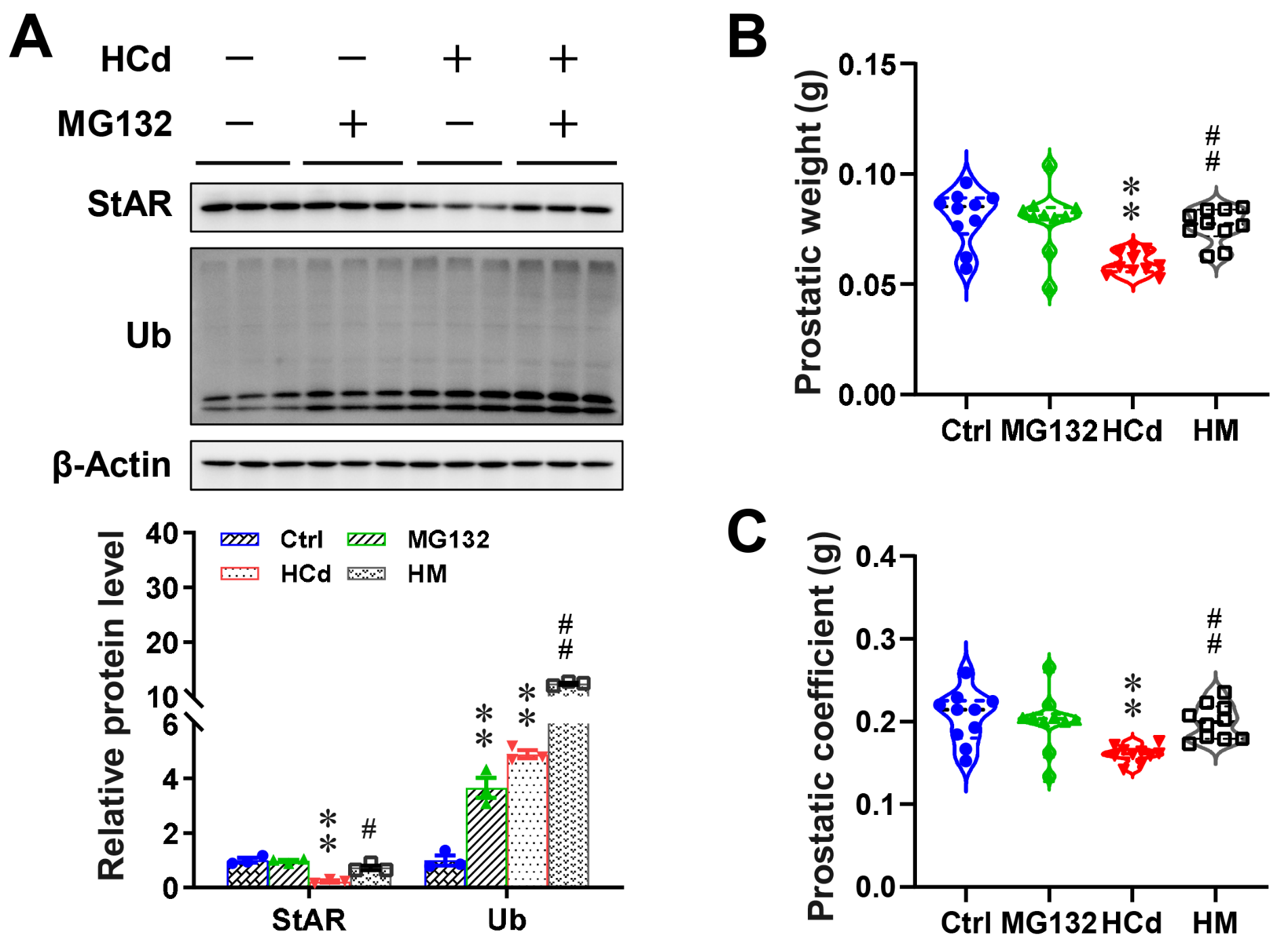


**Fig. S7. Testicular ubiquitin expression in male mice prenatally exposed to Cd and testosterone synthesis.** Pregnant mice (n = 12 per group) were treated with HCd (150 mg/L) from GD8 to GD17 in drinking water and give birth to F1 offspring on GD18. Some of F1 males (n = 10 per group) were treated with MG132 (1μg/per testes/5 days) via testicular injection from PND35-PND70. The F1 males were euthanized on PND70. The F1 testes were collected. (A) StAR and Ub expression in F1 testes (n = 4 per group). (B-C) Prostatic weight and coefficient in F1 offspring on PND70 (n = 10 per group). All data were analyzed using One-way *ANOVA* and presented as *mean* ± *SEM*. Numeric data are provided in Excel Table S19. **P*＜0.05, ***P*＜0.01, compared to Ctrl. ^#^*P* < 0.05, ^##^*P* < 0.01, compared to HCd.


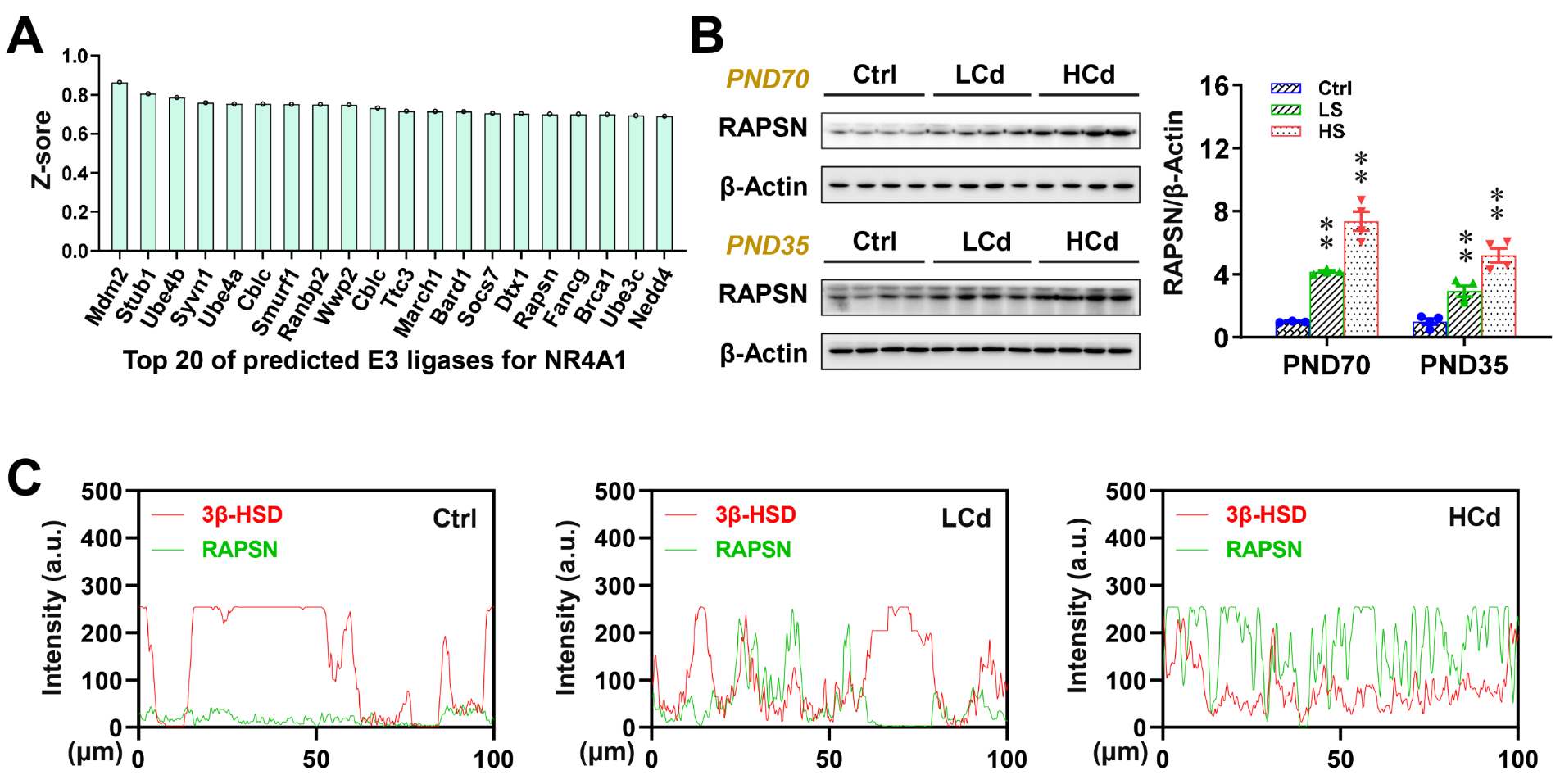


**Fig. S8. The effect of prenatal Cd exposure on RAPSN expression in F1 testes.** (A) Potential TOP 20 E3s for NR4A1. (B-C) Pregnant mice (n = 15 per group) were treated with LCd (50 mg/L) or HCd (150 mg/L) from GD8 to GD17 in drinking water and gave birth to F1 offspring on GD18. Some of F1 males were euthanized on PND35 and PND70, respectively. The F1 testes were collected. (B) RAPSN expression in F1 testes on PND35 and PND70 (n = 4 per group). (C) Immunofluorescent analysis for 3β-HSD and RAPSN in F1 testes on PND35 (n = 4 per group). All data were analyzed using One-way *ANOVA* and presented as *mean* ± *SEM*. Numeric data are provided in Excel Table S20. **P*＜0.05, ***P*＜0.01, compared to Ctrl group.


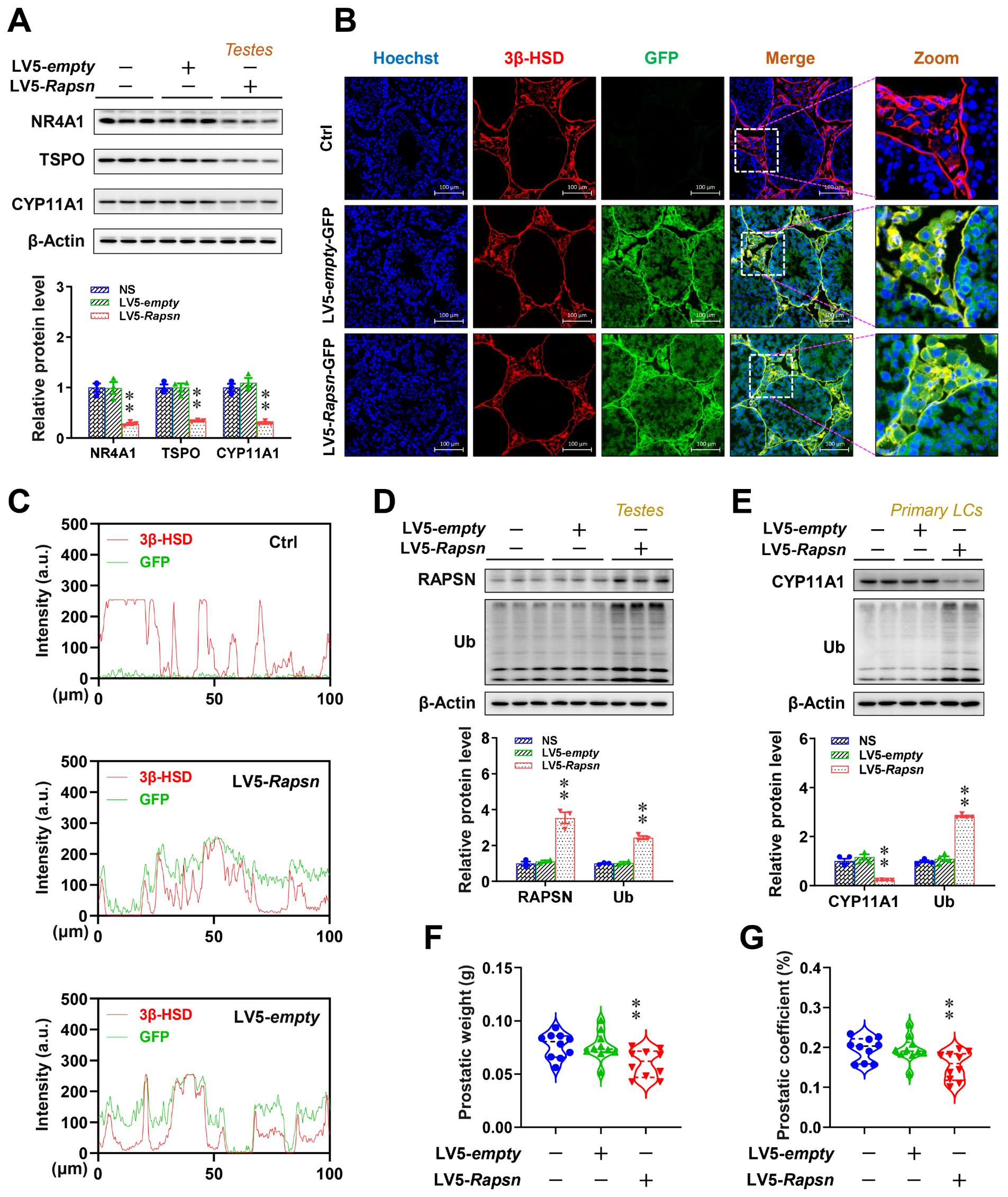


**Fig. S9. The effect of testicular RAPSN overexpression on testosterone synthesis.** The WT and untreated males (n = 10 per group) were injected with LV encoding *Rapsn* (1×10^9^ TU/ml) in testes to produce testicular RAPSN-overexpressed mice. The sera and testis were collected after euthanasia. (A) NR4A1, TSPO and CYP11A1 expression in the testes (n = 3 per group). (B-C) Immunofluorescent analysis and quantification for 3β-HSD and GFP in the testes (n = 3 per group). (D) RAPSN and Ub expression in the testes (n = 3 per group). (E) CYP11A1 and Ub expression in the primary Leydig cells (n = 4 per group). (F-G) Prostatic weight and coefficient in the males (n = 10 per group). All data were analyzed using One-way *ANOVA* and presented as *mean* ± *SEM*. Numeric data are provided in Excel Table S21. **P*＜0.05, ***P*＜0.01, compared to Ctrl group.


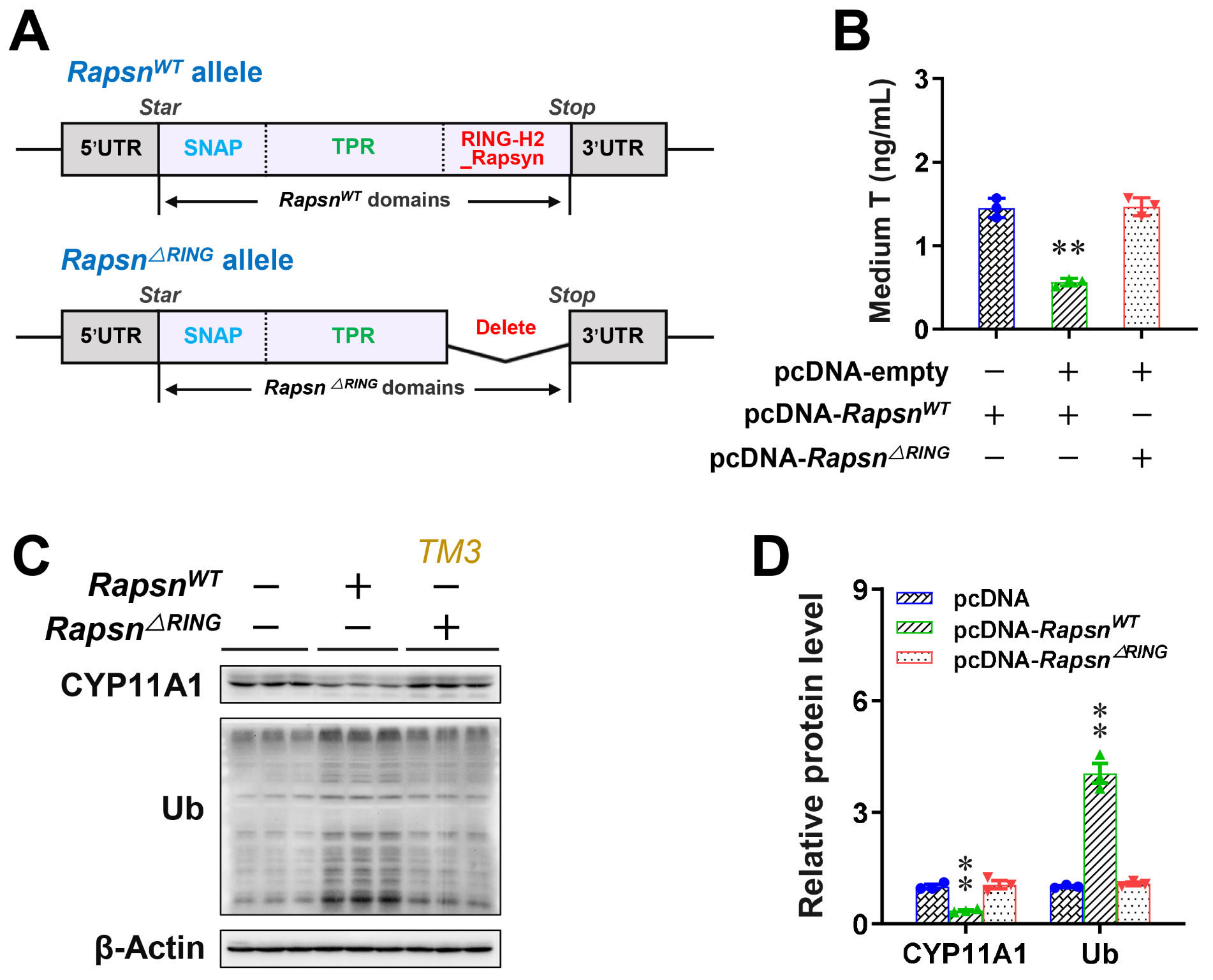


**Fig. S10. The role of RING-H2_Rapsyn domain of RAPSN in NR4A1 ubiquitin expression in the Leydig cells.** The TM3 cells were transfected with the plasmids expressing RAPSN^WT^ or RAPSN^△RING^. (A) The schematic diagram of plasmids expressing RAPSN^WT^ or RAPSN^△RING^. (B) Testosterone content in cell medium (n = 3 per group). (C-D) CYP11A1 and Ub expression in the cells (n = 3 per group). All data were analyzed using One-way *ANOVA* and presented as *mean* ± *SEM*. Numeric data are provided in Excel Table S22. ***P*＜0.01, compared to pcDNA-empty group.


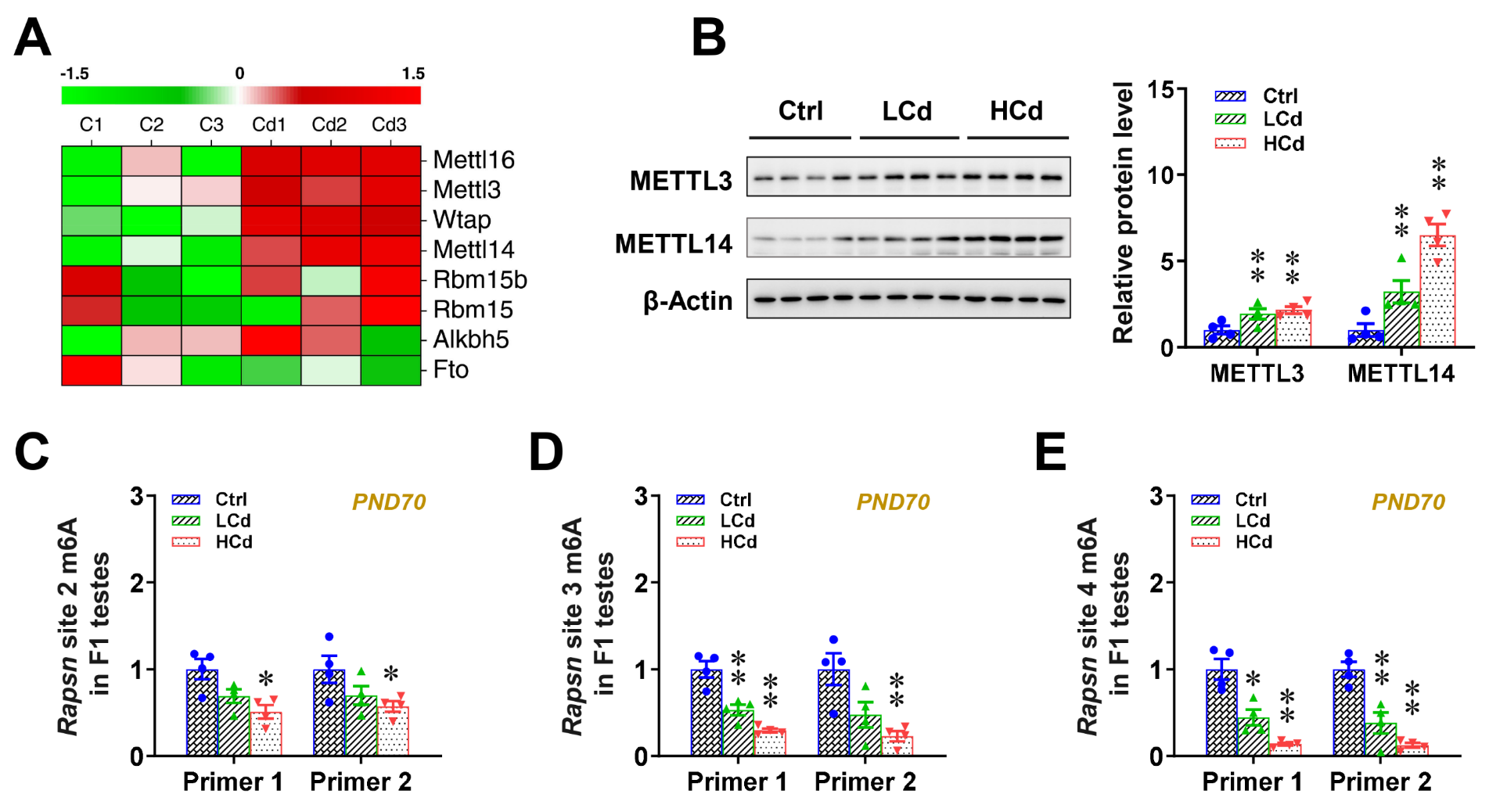


**Fig. S11. The effect of prenatal Cd exposure on METTL3/14 expression in F1 testes.** Pregnant mice (n = 15 per group) were treated with LCd (50 mg/L) or HCd (150 mg/L) from GD8 to GD17 in drinking water and gave birth to F1 offspring on GD18. Some of F1 males were euthanized on PND70. The F1 testes were collected. (A) Global transcriptome analysis heatmap for m^6^A methyltransferases and demethylases (n = 3 per group). (B) METTL3 and METTL14 expression in PND70 testes (n = 4 per group). (C-D) The m^6^A- methylated levels in *Rapsn* mRNA site 2-4 in F1 testes on PND70 (n = 4 per group). All data were analyzed using One-way *ANOVA* and presented as *mean* ± *SEM*. Numeric data are provided in Excel Table S23. ***P*＜0.01, compared to Ctrl group.


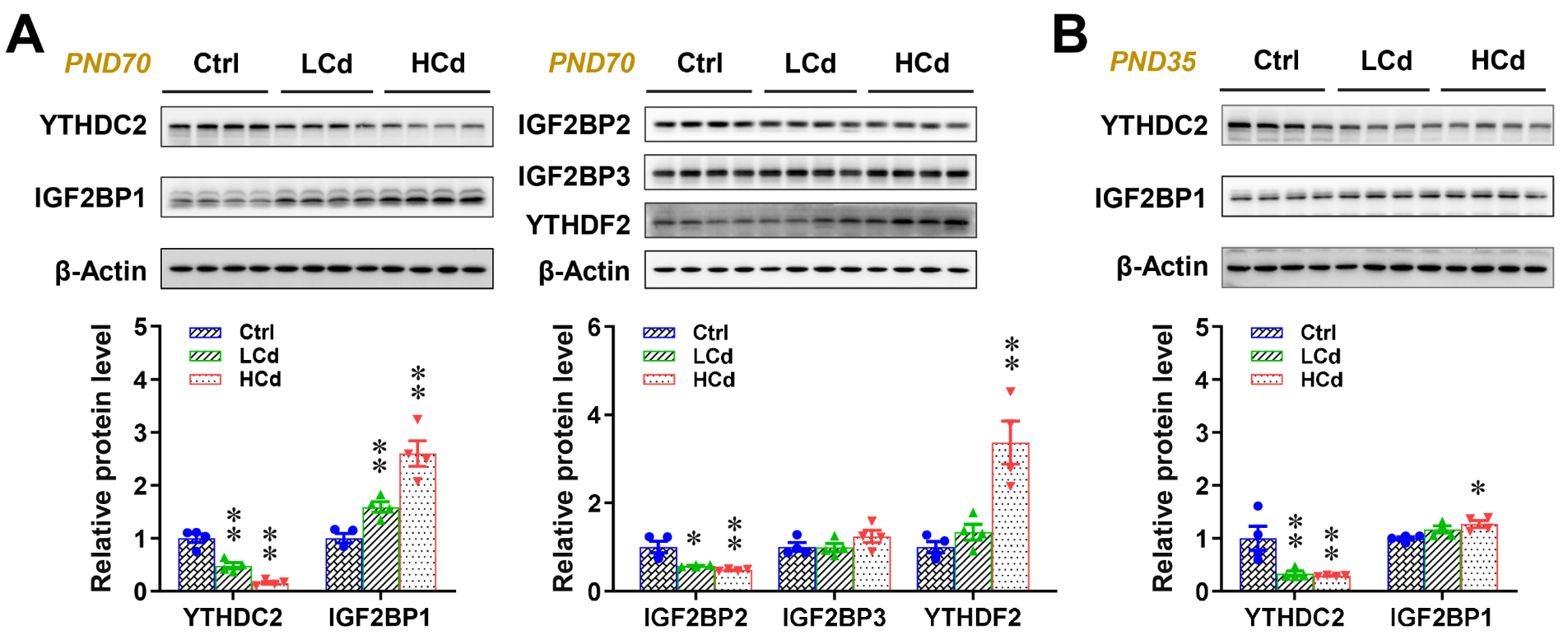


**Fig. S12. The effect of prenatal Cd exposure on m^6^A readers expression in F1 testes.** Pregnant mice (n = 15 per group) were treated with LCd (50 mg/L) or HCd (150 mg/L) from GD8 to GD17 in drinking water and gave birth to F1 offspring on GD18. Some of F1 males were euthanized on PND35 and PND70, respectively. The F1 testes were collected. (A) YTHDC2, IGF2BP1, IGF2BP2, IGF2BP3 and YTHDF2 expression in PND70 testes (n = 4 per group). (B) YTHDC2 and IGF2BP1 expression in PND35 testes (n = 4 per group). All data were analyzed using One-way *ANOVA* and presented as *mean* ± *SEM*. Numeric data are provided in Excel Table S24. ***P*＜0.01, compared to Ctrl group.


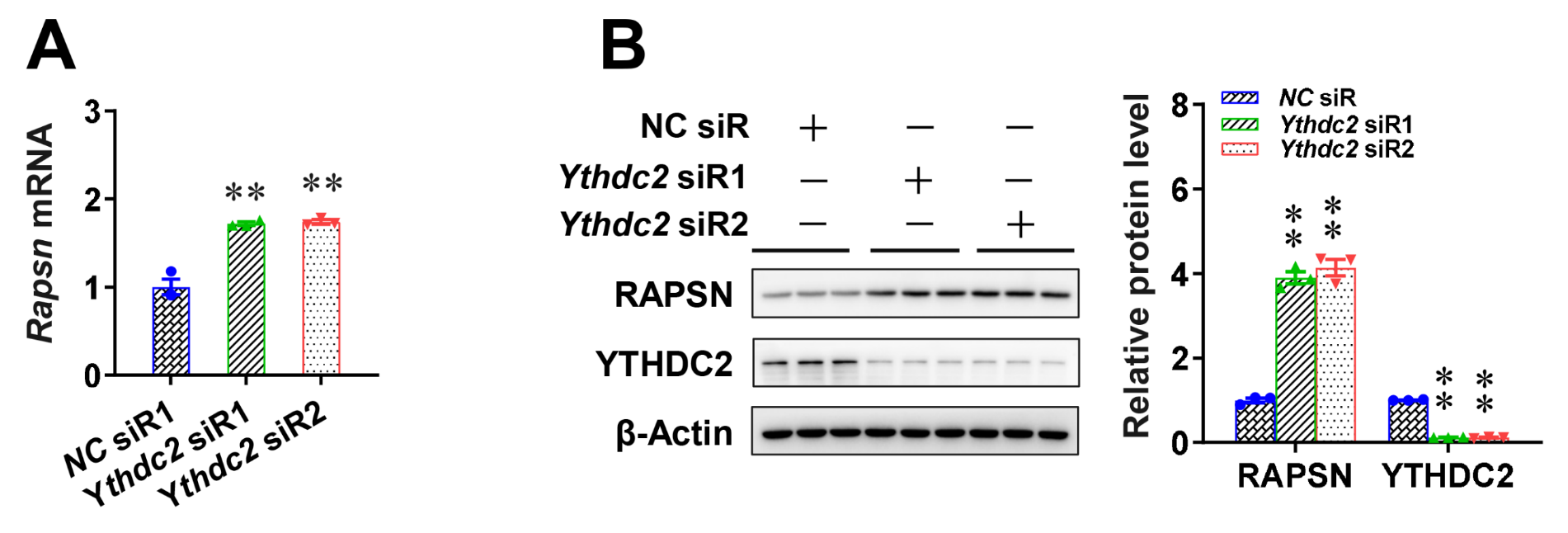


**Fig. S13. The effect of YTHDC2 knockdown on RAPSN expression in the Leydig cells.** The TM3 cells were transfected with *Ythdc2* siRs. (A) The level of Rapsn mRNA in the cells (n = 3 per group). (B) RAPSN and YTHDC2 expression in the cells (n = 3 per group). All data were analyzed using One-way *ANOVA* and presented as *mean* ± *SEM*. The numeric data are provided in Excel Table S25. **P*＜0.05, ***P*＜0.01, compared to NC siR group.


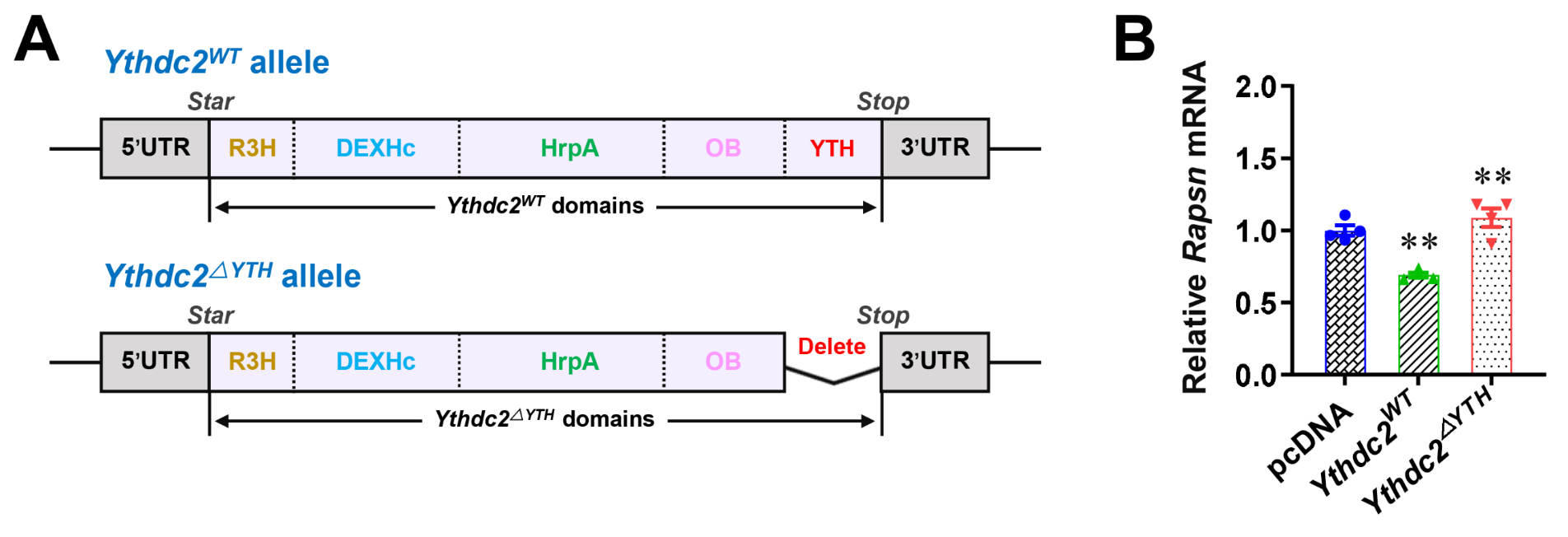


**Fig. S14.** **The role of YTH domain of YTHDC2 in RAPSN expression in TM3 cells.** The TM3 cells were treated using plasmids expressing YTHDC2^WT^ or YTHDC2^△YTH^. (A) The schematic diagram of plasmids expressing YTHDC2^WT^ or YTHDC2^△YTH^. (B) Relative *Rapsn* mRNA level (n = 3 per group). All data were analyzed using One-way *ANOVA* and presented as *mean* ± *SEM*. Numeric data are provided in Excel Table S26. ***P*＜0.01, compared to pcDNA group.


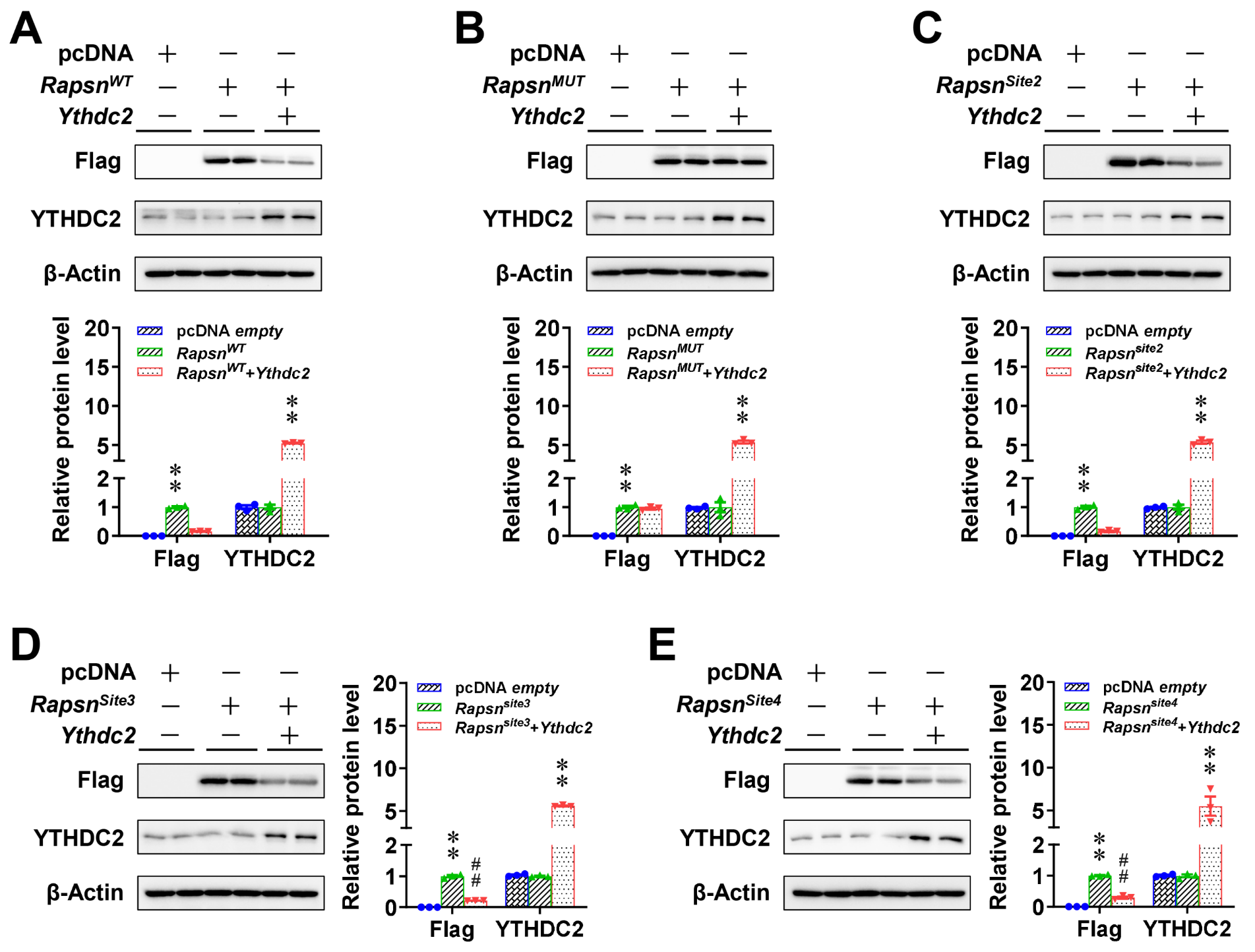


**Fig. S15. m^6^A modification in site2, 3 and 4 was not necessary for YTHDC2 degrading *Rapsn* mRNA.** (A) The TM3 cells were treated using plasmids expressing *Rapsn^WT^* with or without plasmids expressing YTHDC2^WT^. Flag and YTHDC2 expression in the cells (n = 3 per group). (B) The TM3 cells were treated using plasmids expressing *Rapsn^MUT^* with or without plasmids expressing YTHDC2^WT^. Flag and YTHDC2 expression in the cells (n = 3 per group). (C) The TM3 cells were treated using plasmids expressing *Rapsn^Site2^* with or without plasmids expressing YTHDC2^WT^. Flag and YTHDC2 expression in the cells (n = 3 per group). (D) The TM3 cells were treated using plasmids expressing *Rapsn^Site3^* with or without plasmids expressing YTHDC2^WT^. Flag and YTHDC2 expression in the cells (n = 3 per group). (E) The TM3 cells were treated using plasmids expressing *Rapsn^Site4^* with or without plasmids expressing YTHDC2^WT^. Flag and YTHDC2 expression in the cells (n = 3 per group). All data were analyzed using One-way *ANOVA* and presented as *mean* ± *SEM*. Numeric data are provided in Excel Table S27. ***P*＜0.01, compared to pcDNA-empty group.


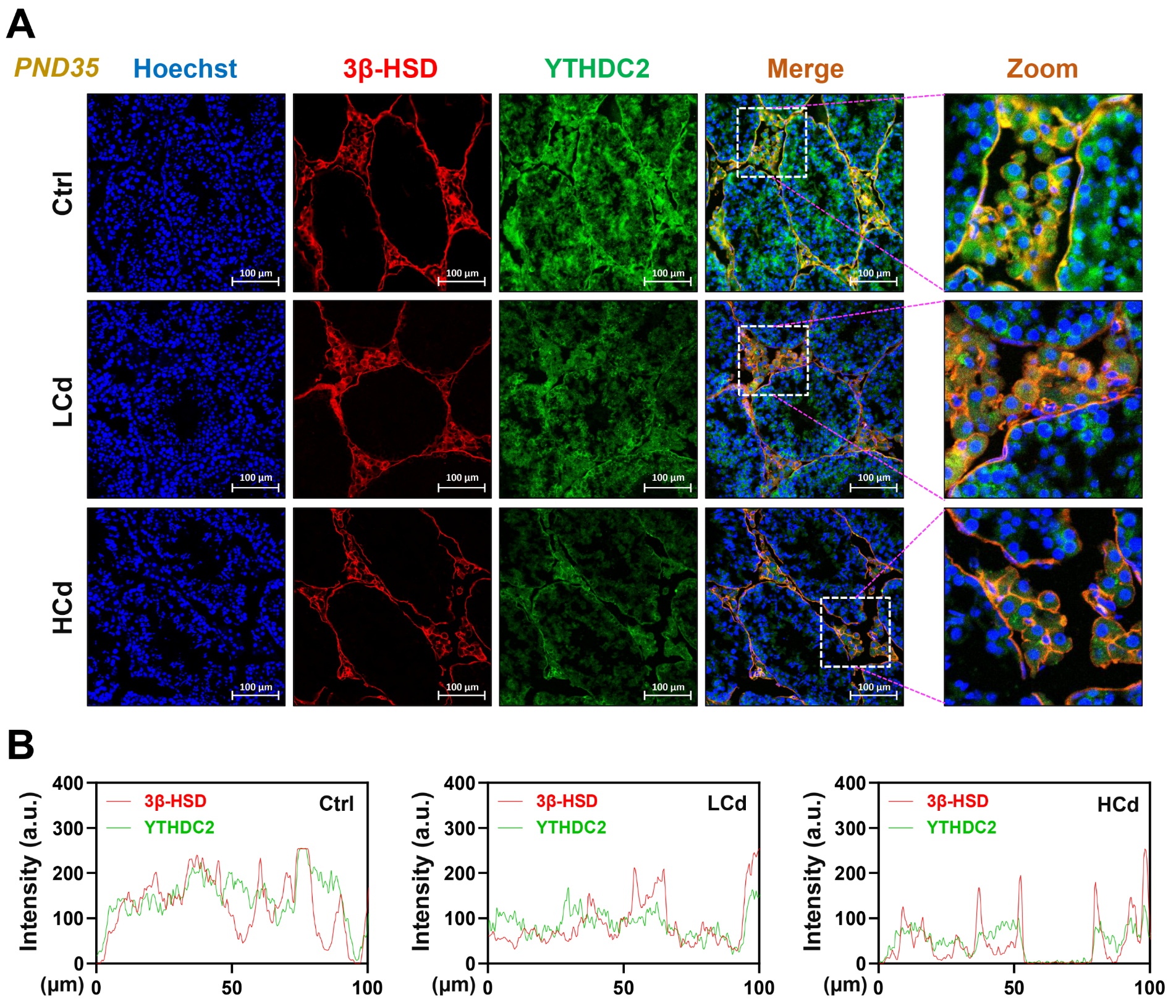


**Fig. S16. The effect of prenatal Cd exposure on YTHDC2 expression in F1 testes.** Pregnant mice (n = 15 per group) were treated with LCd (50 mg/L) or HCd (150 mg/L) from GD8 to GD17 in drinking water and gave birth to F1 offspring on GD18. Some of F1 males were euthanized on PND35. The F1 testes were collected. (A-B) Immunofluorescent quantification of 3β-HSD and YTHDC2 in PND35 testes (n = 4 per group). All data were analyzed using One-way *ANOVA* and presented as *mean* ± *SEM*. Numeric data are provided in Excel Table S28.


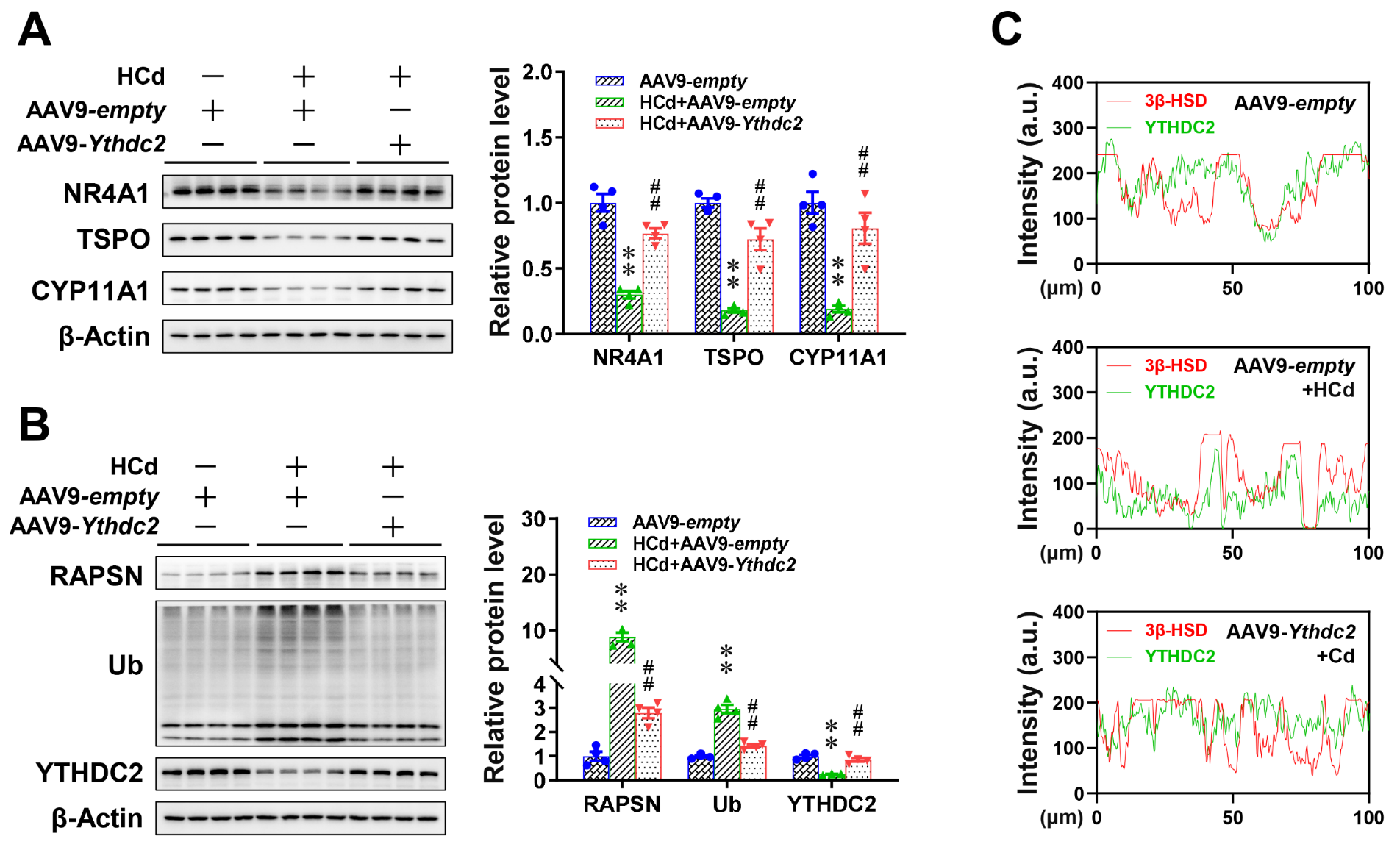


**Fig. S17. Testicular YTHDC2 expression in** **male mice prenatally exposed to Cd and testosterone synthesis.** Pregnant mice (n = 12 per group) were treated with HCd (150 mg/L) from GD8 to GD17 in drinking water. The F1 males were infected with 10 μl AAV9 encoding *Ythdc2* (2×10^11^ VG/mL) via testicular injection on PND35. The F1 testes were collected on PND70. (A-B) RAPSN, Ub, YTHDC2, NR4A1, TSPO and CYP11A1 expression in F1 testes (n = 4 per group). (C) Immunofluorescent quantification of 3β-HSD and YTHDC2 in F1 testes (n = 4 per group). All data were analyzed using One-way *ANOVA* and presented as *mean* ± *SEM*. Numeric data are provided in Excel Table S29. ***P*＜0.01, compared to AAV9-empty group. ^##^*P* < 0.01, compared to HCd.


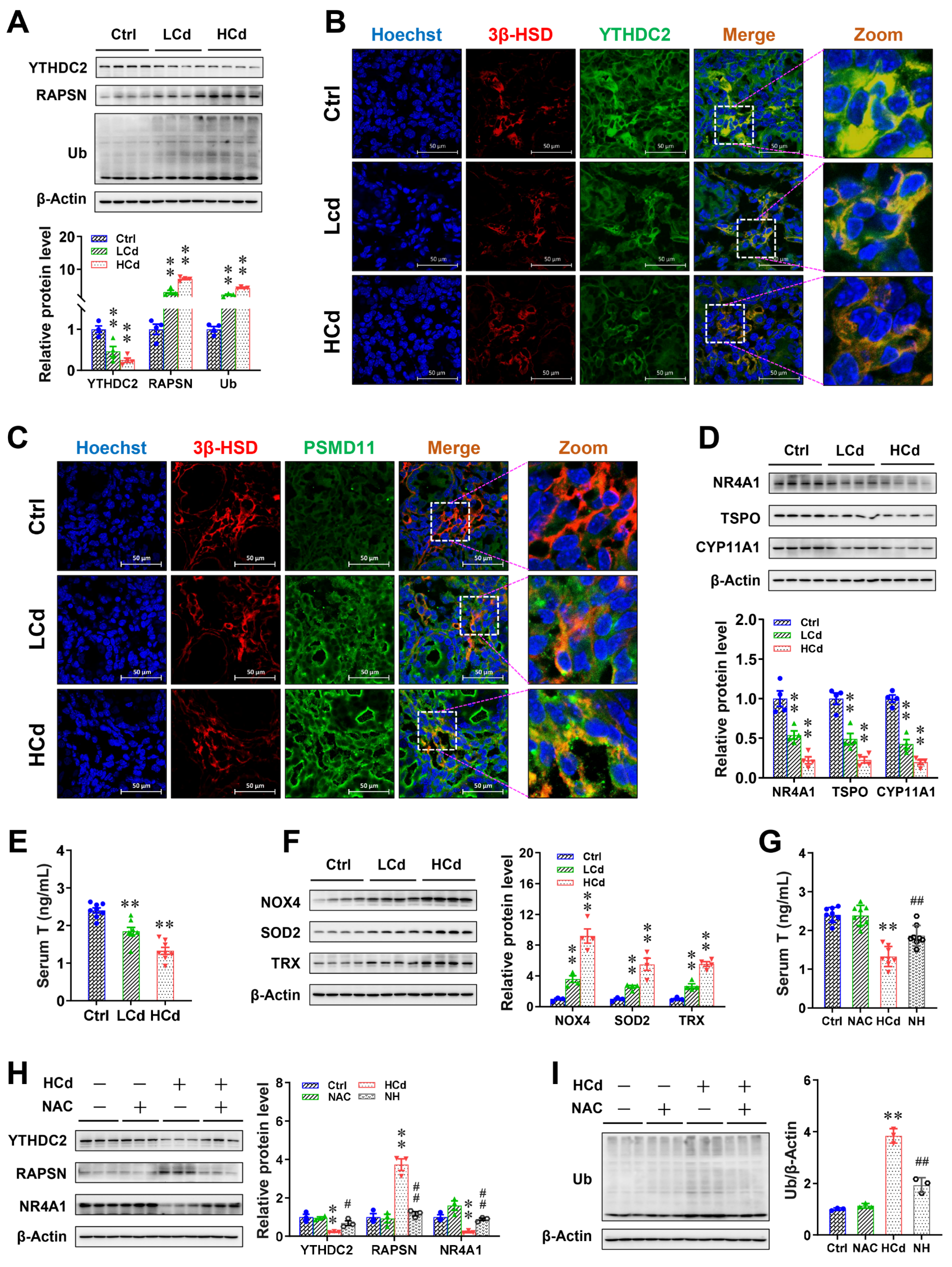


**Fig. S18.** **Fetal testicular oxidative stress in male mice prenatally exposed to Cd and YTHDC2 expression.** (A-F) Pregnant mice (n = 15 per group) were treated with LCd (50 mg/L) or HCd (150 mg/L) from GD8 to GD17 in drinking water. All pregnant mice were euthanized on GD18. The fetal sera and testes were collected. (A) YTHDC2, RAPSN and Ub expression in fetal testes (n = 4 per group). (B) Immunofluorescent analysis for 3β-HSD and YTHDC2 in fetal testes (n = 4 per group). (C) Immunofluorescent analysis for 3β-HSD and PSMD11 in fetal testes (n = 4 per group). (D) NR4A1, TSPO and CYP11A1 expression in fetal testes (n = 4 per group). (E) Testosterone levels in fetal sera (n = 8 per group). (F) NOX4, SOD2 and TRX expression in fetal testes (n = 4 per group). (G-I) Pregnant mice (n = 15 per group) were exposed to HCd (150 mg/L) in drinking water with or without NAC supplement (500mg/kg/day, i.g.) from GD7-GD17. All pregnant mice were euthanized on GD18. The fetal sera and testes were collected. (G) T levels in fetal sera (n = 8 per group). (H-I) YTHDC2, RAPSN, NR4A1 and Ub expression in fetal testes (n = 3 per group). All data were analyzed using one-way *ANOVA* and presented as *means* ± *SEM*. Numeric data are provided in Excel Table S11. **P*＜0.05, ***P*＜0.01, compared to Ctrl group. ^#^*P*＜0.05, ^##^*P*＜0.01, compared to HCd group.


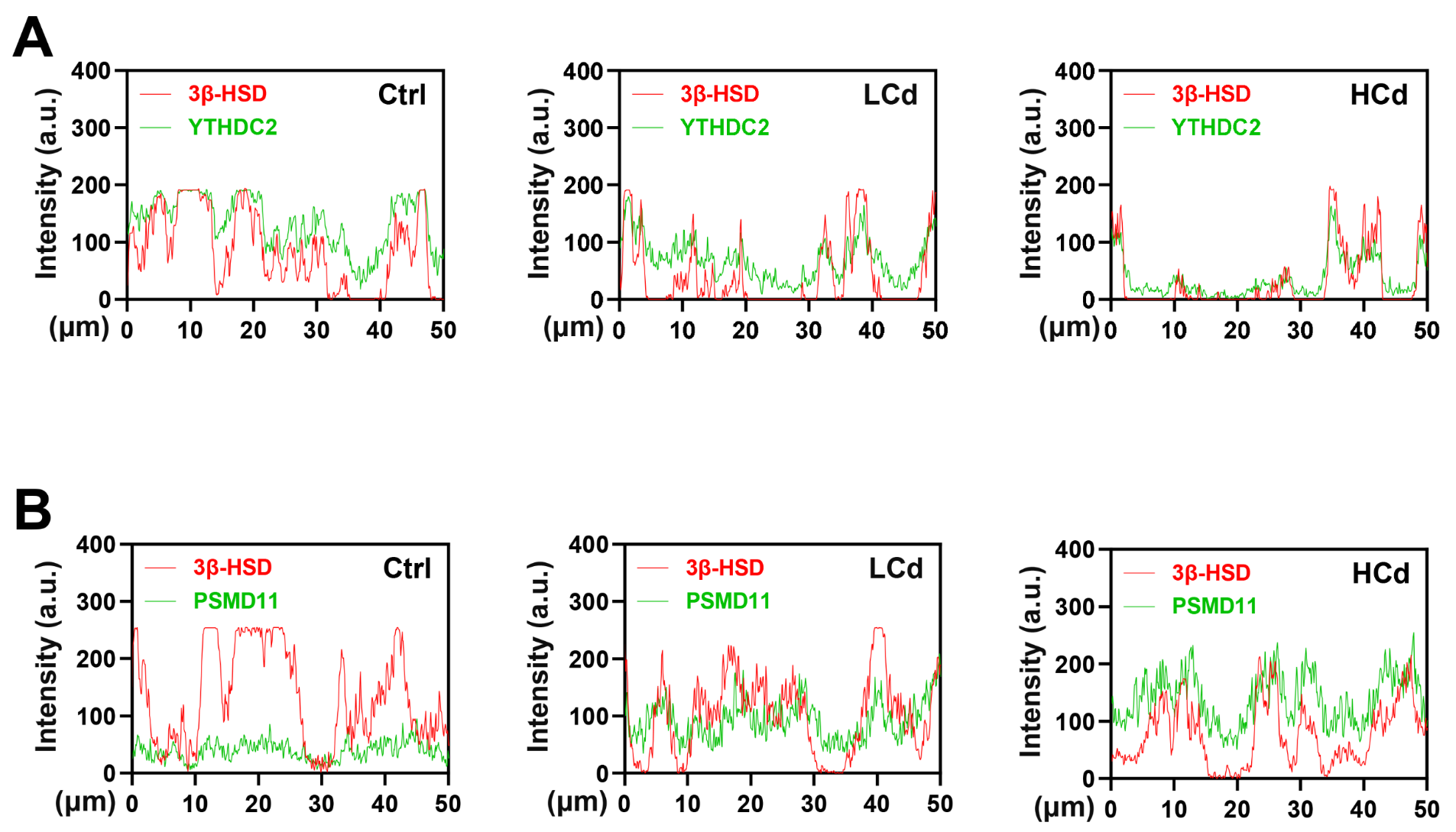


**Fig. S19.** **Th effect of prenatal** **Cd exposure on YTHDC2 and ubiquitin expressions in fetal testes.** Pregnant mice (n = 15 per group) were treated with LCd (50 mg/L) or HCd (150 mg/L) from GD8 to GD17 in drinking water. All pregnant mice were euthanized on GD18 under anesthesia. The fetal testes were collected. (A) Immunofluorescent quantification for 3β-HSD and YTHDC2 in fetal testes (n = 4 per group). (B) Immunofluorescent quantification for 3β-HSD and PSMD11 in fetal testes (n = 4 per group). All data were analyzed using One-way *ANOVA* and presented as *mean* ± *SEM*. Numeric data are provided in Excel Table S30.


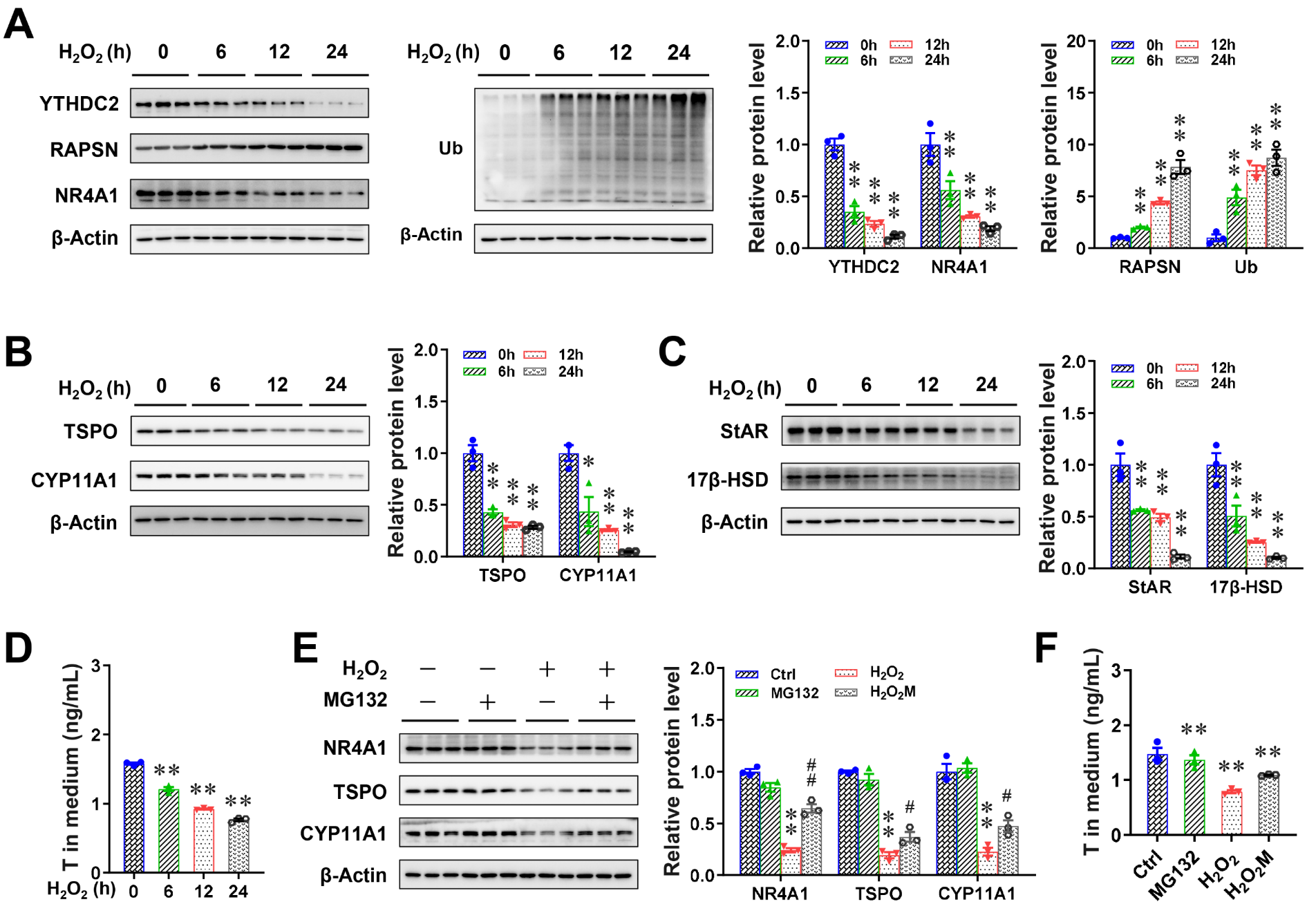


**Fig. S20. The role of oxidative stress in Cd-induced YTHDC2 reduction and ubiquitination modification in the Leydig cells.** (A-D) TM3 cells were treated with H_2_O_2_ (100 μM) for 0, 6, 12 and 24 h. (A) YTHDC2, RAPSN, NR4A1 and Ub expression in the cells (n = 3 per group). (B-C) TSPO, CYP11A1, StAR and 17β-HSD expression in the cells (n = 3 per group). (D) Testosterone content in the cell medium (n = 3 per group). (E-F) TM3 cells were treated using H_2_O_2_ (100 μM) for 12 h with or without MG132 (5 μM) pretreatment for 1 h (n = 3 per group). (E) NR4A1, TSPO and CYP11A1 expression in the cells (n = 3 per group). (F) Testosterone content in the cell medium (n = 3 per group). All data were analyzed using One-way *ANOVA* and presented as *mean* ± *SEM*. Numeric data are provided in Excel Table S31. ***P*＜0.01, compared to 0 h group. ^#^*P*＜0.05, ^##^*P*＜0.01, compared to H_2_O_2_ group.


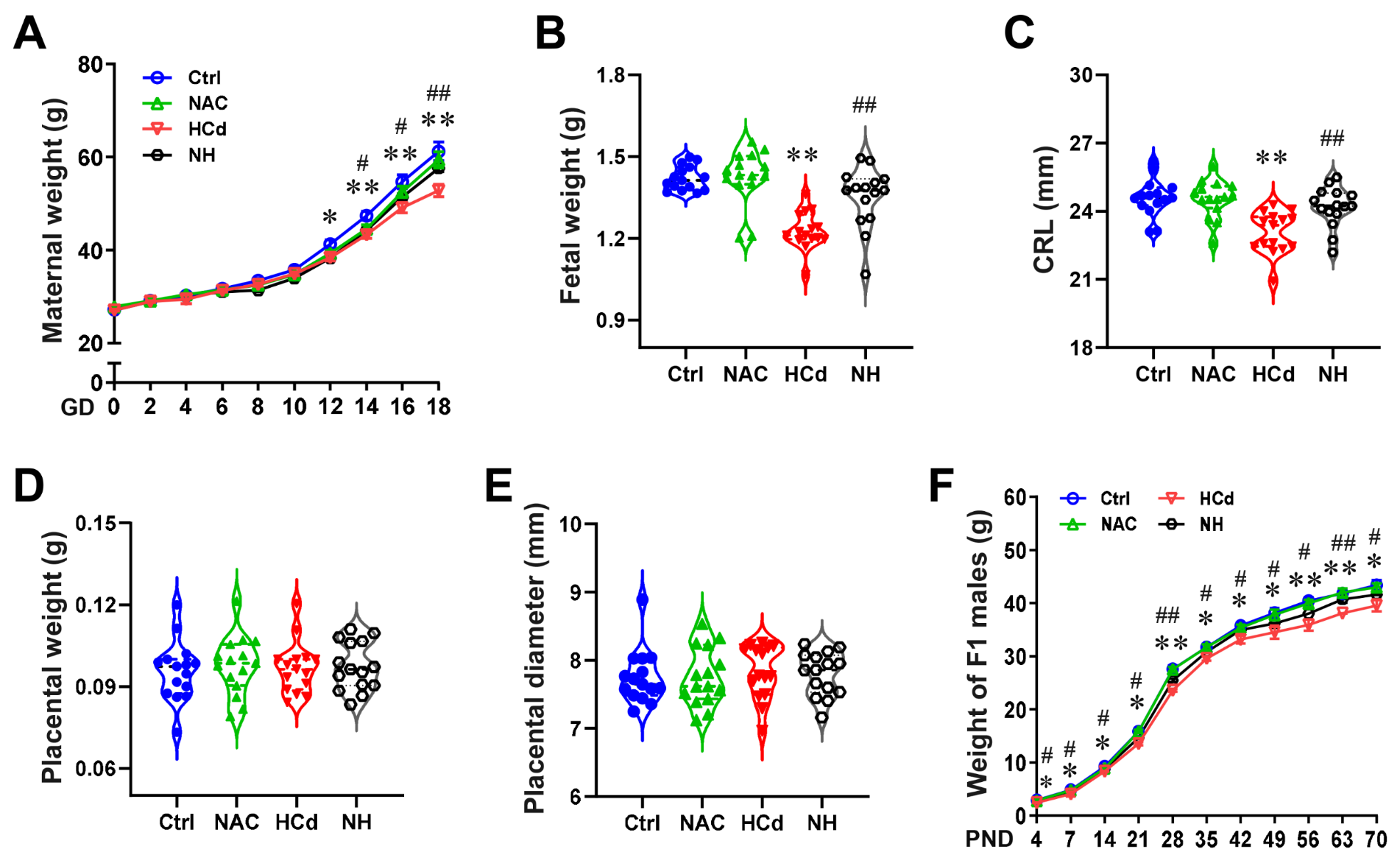


**Fig. S21. The effect of NAC supplement on fetal growth upon prenatal Cd exposure.** Pregnant mice (n = 15 per group) were exposed to HCd (150 mg/L) in drinking water with or without NAC supplement (500 mg/kg/day, i.g.) from GD7 to GD17. All pregnant mice were euthanized on GD18 under anesthesia. Fetal and placental sizes were recorded. (A) Maternal weight (n = 15 per group). (B) Fetal weight (n = 15 per group). (C) Fetal crown-rump length (n = 15 per group). (D) Placental weight (n = 15 per group). (E) Placental diameter (n = 15 per group). (F) Male offspring weight (n = 15 per group). All data were analyzed using One-way *ANOVA* and presented as *mean* ± *SEM*. Numeric data are provided in Excel Table S32. **P*＜0.05, ***P*＜0.01, compared to Ctrl group. ^#^*P*＜0.05, ^##^*P*＜0.01, compared to HCd group.


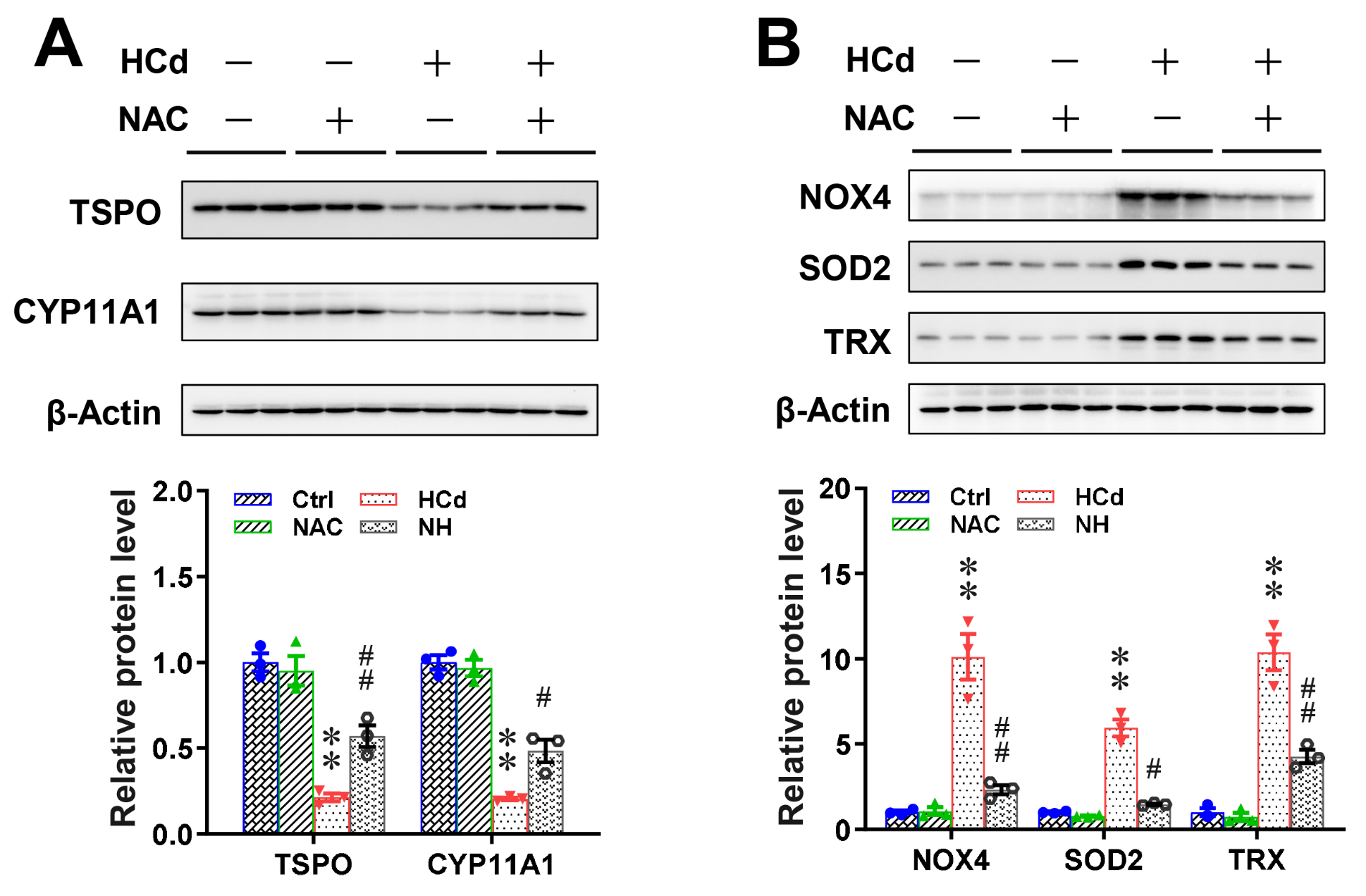


**Fig. S22. Oxidative stress in fetal testes prenatally exposed to Cd and testicular testosterone synthesis.** Pregnant mice (n = 15 per group) were exposed to HCd (150 mg/L) in drinking water with or without NAC supplement (500 mg/kg/day, i.g.) from GD7 to GD17. All pregnant mice were euthanized on GD18 under anesthesia. The fetal testes were collected. (A) TSPO and CYP11A1 expression in fetal testes (n = 3 per group). (B) NOX4, SOD2 and TRX expression in fetal testes (n = 3 per group). All data were analyzed using One-way *ANOVA* and presented as *mean* ± *SEM*. Numeric data are provided in Excel Table S32. ***P*＜0.01, compared to Ctrl group. ^#^*P*＜0.05, ^##^*P*＜0.01, compared to HCd group.


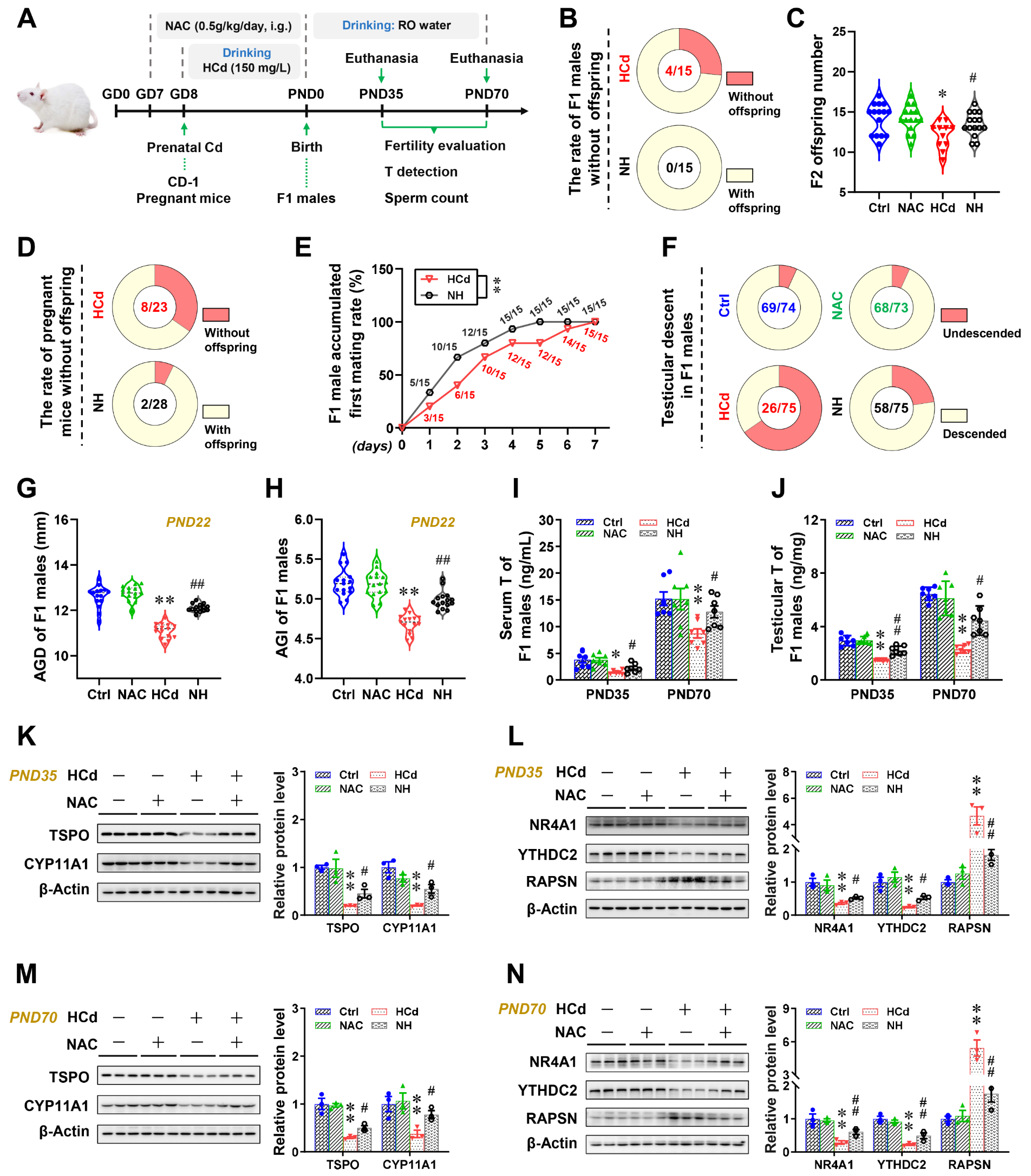


**Fig.23. The effect of fetal YTHDC2 expression on testosterone synthesis and fertility in F1 males prenatally exposed to Cd.** Pregnant mice (n = 15 per group) were exposed to HCd (150 mg/L,) in drinking water with or without NAC supplement (500 mg/kg/day, i.g.) from GD7-GD17 and delivered naturally on GD18. Some of F1 males were euthanized on PND35 and PND70, respectively. The F1 sera and testes were collected. Some of F1 males were mated with WT untreated females at a ratio of 1:2. (A) The work model of the experiment. (B) The rate of F1 males without offspring (n = 15 per group). (C) The number of F2 offspring (n = 15 in Ctrl/NAC/NH, n = 11 in HCd). (D) The rate of pregnant mice without offspring (n = 23 in HCd, n =28 in NH). (E) F1 male accumulated first mating rate within seven days (n = 15 per group). (F) Testicular descent rate in F1 males (n = 74 in Ctrl, 73 in NAC, n = 75 in HCd/NH). (G-H) F1 male AGD and AGI (n = 15 per group). (J) T levels in F1 sera (n = 7 per group) and testes (n = 7 per group). (K-N) TSPO, CYP11A1, NR4A1, YTHDC2 and RAPSN expression in PND35 and PND70 testes (n = 3 per group). All data presented as *means* ± *SEM*. Repeated-measures *ANOVA* was applied for E. One-way *ANOVA* was applied for C and G-N. The numeric data are provided in Excel Table S12. **P*＜0.05, ***P*＜0.01, compared to Ctrl group. ^#^*P*＜0.05, ^##^*P*＜0.01, compared to HCd group.


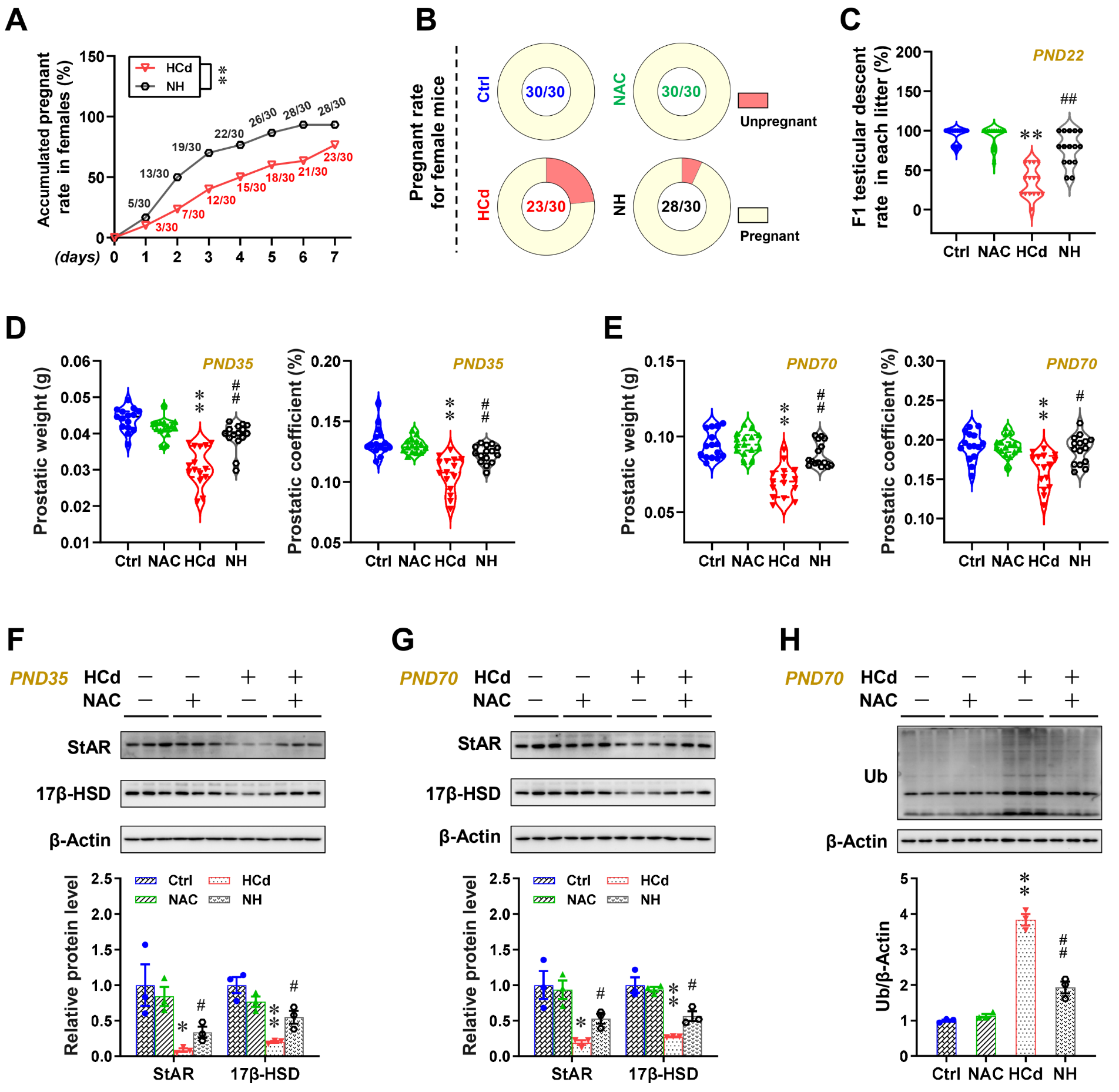


**Fig. S24. The effect of fetal YTHDC2 expression on testosterone synthesis and fertility in F1 males prenatally exposed to Cd.** Pregnant mice (n = 15 per group) were exposed to HCd (150 mg/L) in drinking water with or without NAC supplement (500 mg/kg/day, i.g.) from GD7 to GD17 and gave birth to F1 offspring naturally on GD18. Some of F1 males were euthanized on PND35 and PND70, respectively. The F1 testes were collected after euthanasia. Some of F1 males were mated with WT and untreated females at a ratio of 1:2. (A) Accumulated pregnancy rate in females with seven days (n = 30 per group). (B) Pregnant rate of females after mating for seven days (n = 30 per group). (C) Testicular descent rate (n = 15 per group). (D-E) Prostatic weight and coefficient in F1 males (n = 15 per group). (F-G) StAR and 17β-HSD expression in F1 testes on PND35 and PND70 (n = 4 per group). (H) Ub expression in PND70 testes (n = 4 per group). All data presented as *mean* ± *SEM*. Repeated-measures *ANOVA* was applied for A. One-way *ANOVA* was applied for C-H. Numeric data are provided in Excel Table S34. **P*＜0.05, ***P*＜0.01, compared to Ctrl, ^#^*P*＜0.05, ^##^*P*＜0.01, compared to HCd group.


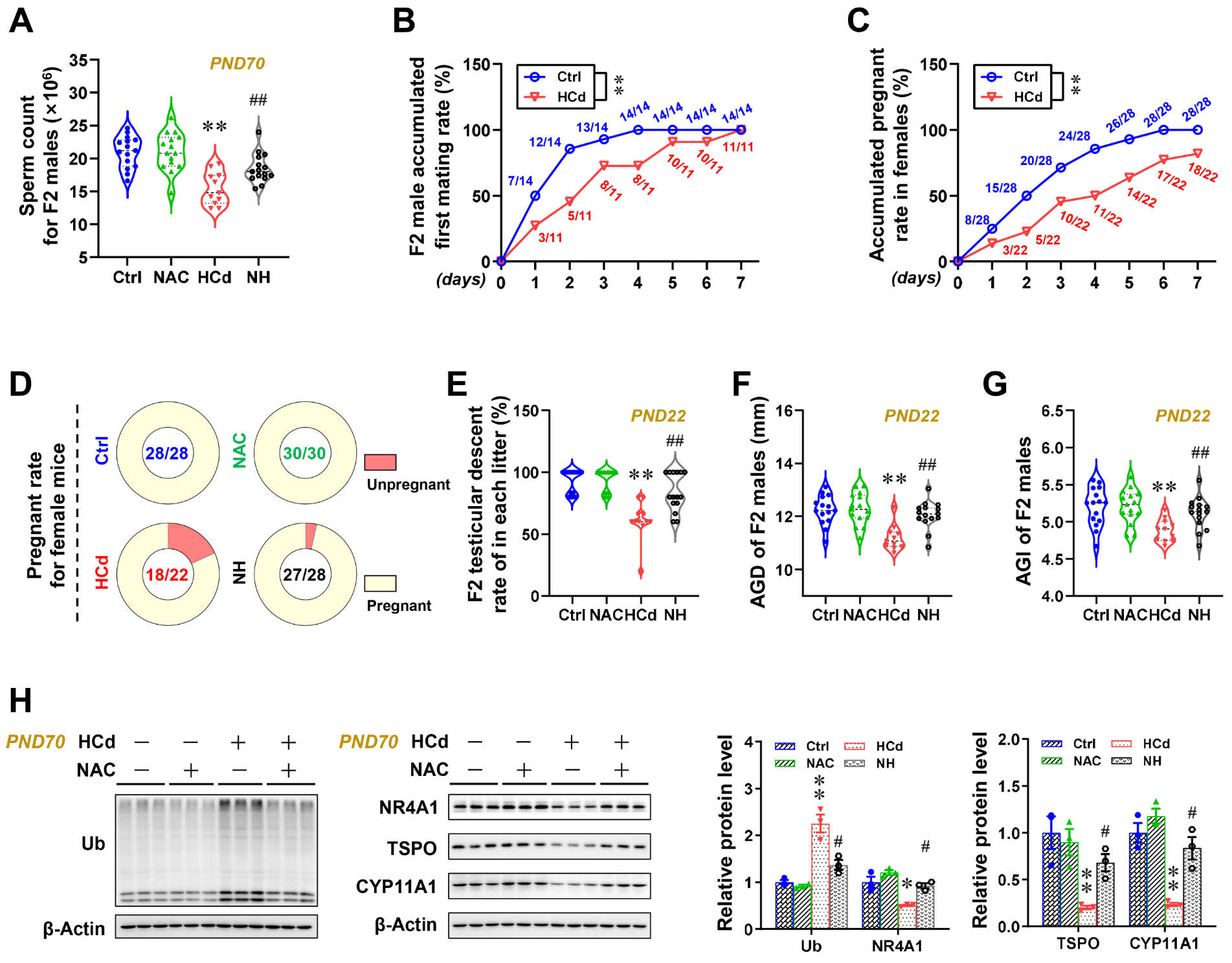


**Fig. S25. The effect of fetal YTHDC2 expression in F1 males prenatally exposed to Cd on testosterone synthesis and fertility in F2 males.** Pregnant mice (n = 15 per group) were exposed to HCd (150 mg/L) in drinking water with or without NAC supplement (500 mg/kg/day, i.g.) from GD7 to GD17 and gave birth to F1 offspring on GD18. Some of F1 males were mated with WT and untreated females to produce F2 offspring. Some of F2 males were euthanized on PND70. Some of F2 males were mated with WT and untreated females at a ratio of 1:2 to produce F3 offspring. (A) Sperm count for F2 males (n = 15 in Ctrl/NAC/NH, n = 11 in HCd). (B) F2 male accumulated first mating rate within seven days (n = 14 in Ctrl, n = 11 in HCd). (C) Female accumulated pregnant rate within seven days (n = 28 in Ctrl, n = 22 in HCd). (D) Pregnant rate for female mice after mating for seven days (n = 28 in Ctrl/NH, n=30 in NAC, n = 22 in HCd). (E) Testicular descent rate in F2 males (n = 15 in Ctrl/NAC/NH, n = 11 in HCd). (F-G) F2 male AGD and AGI (n = 15 in Ctrl/NAC/NH, n = 11 in HCd). (H) Ub, NR4A1, TSPO and CYP11A1 expression in F2 testes on PND70 (n = 3 per group). All data presented as *mean* ± *SEM*. Repeated-measures ANOVA was applied for B-C. One-way *ANOVA* was applied for A and E-H. The numeric data are provided in Excel Table S35. **P*＜0.05, ***P*＜0.01, compared to Ctrl, ^#^*P*＜0.05, ^##^*P*＜0.01, compared to HCd group.


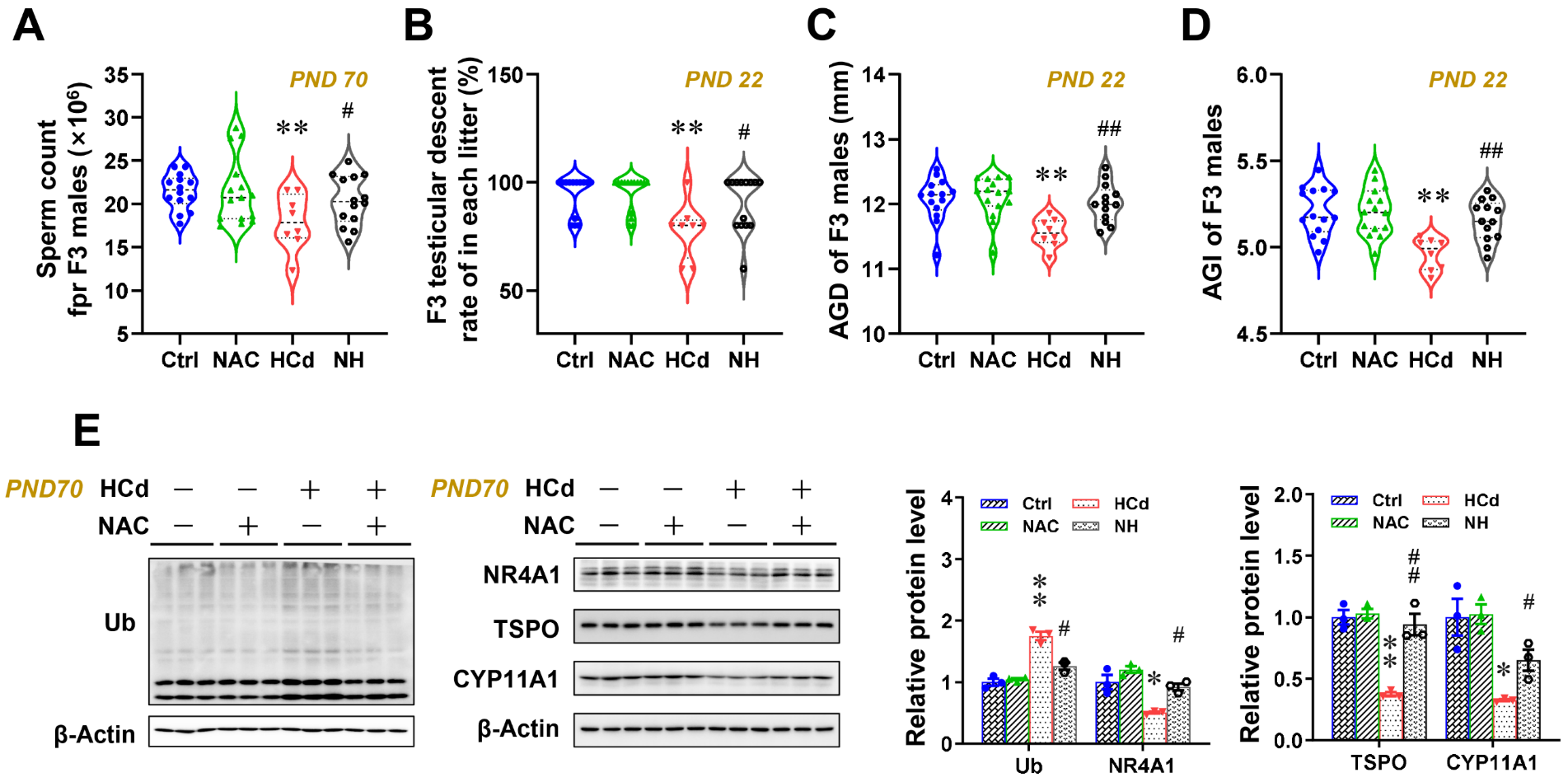


**Fig. S26. The effect of fetal YTHDC2 expression in F1 males prenatally exposed to Cd on testosterone synthesis and fertility in F3 males.** Pregnant mice (n = 15 per group) were exposed to HCd (150 mg/L) in drinking water with or without NAC supplement (500 mg/kg/day, i.g.) from GD7 to GD17 and gave birth to F1 offspring on GD18. Some of F1 males were mated with WT and untreated females to produce F2 offspring. Some of F2 males were mated with WT and untreated females at a ratio of 1:2 to produce F3 offspring. Some of F3 males were euthanized on PND70. (A) Sperm count for F3 males (n = 14 in Ctrl/NH, n = 15 in NAC, n = 8 in HCd). (B) Testicular descent rate in F3 males (n = 14 in Ctrl/NH, n = 15 in NAC, n = 8 in HCd). (C-D) F3 male AGD and AGI (n = 14 in Ctrl/NH, n = 15 in NAC, n = 8 in HCd). (E) Ub, NR4A1, TSPO and CYP11A1 expression in F3 testes on PND70 (n = 3 per group). All data were analyzed using One-way *ANOVA* and presented as *mean* ± *SEM*. Numeric data are provided in Excel Table S36. **P*＜0.05, ***P*＜0.01, compared to Ctrl, ^#^*P*＜0.05, ^##^*P*＜0.01, compared to HCd group.


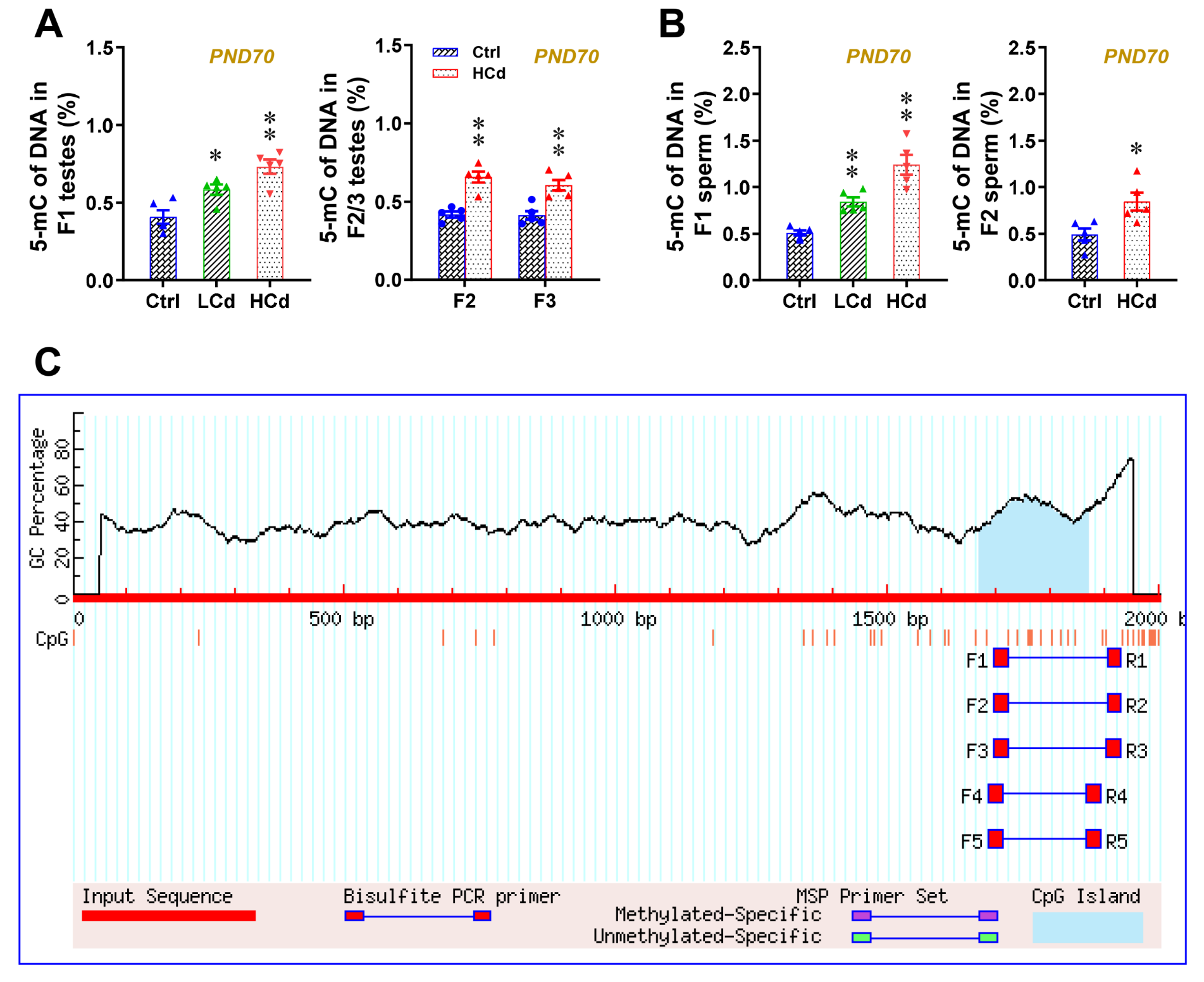


**Fig. S27. The effect of prenatal Cd exposure on 5-mC levels of testes and sperm in F1, F2 and F3 males.** (A-B) Pregnant mice (n = 15 per group) were exposed to HCd (150 mg/L) in drinking water with or without NAC supplement (500 mg/kg/day, i.g.) from GD7 to GD17 and gave birth to F1 offspring on GD18. Some of F1 males were mated with WT and untreated females to produce F2 offspring. Some of F2 males were mated with WT and untreated females at a ratio of 1:2 to produce F3 offspring. Some of F3 males were euthanized on PND70. (A) 5-mC levels in DNA of F1, F2 and F3 testes (n = 5 per group). (B) 5-mC levels in DNA of F1 and F2 sperm (n = 5 per group). (C) CpG island prediction of the YTHDC2 promoter using MethPrimer database. All data were analyzed using One-way *ANOVA* and presented as *mean* ± *SEM*. Numeric data are provided in Excel Table S37. **P*＜0.05, ***P*＜0.01, compared to Ctrl.


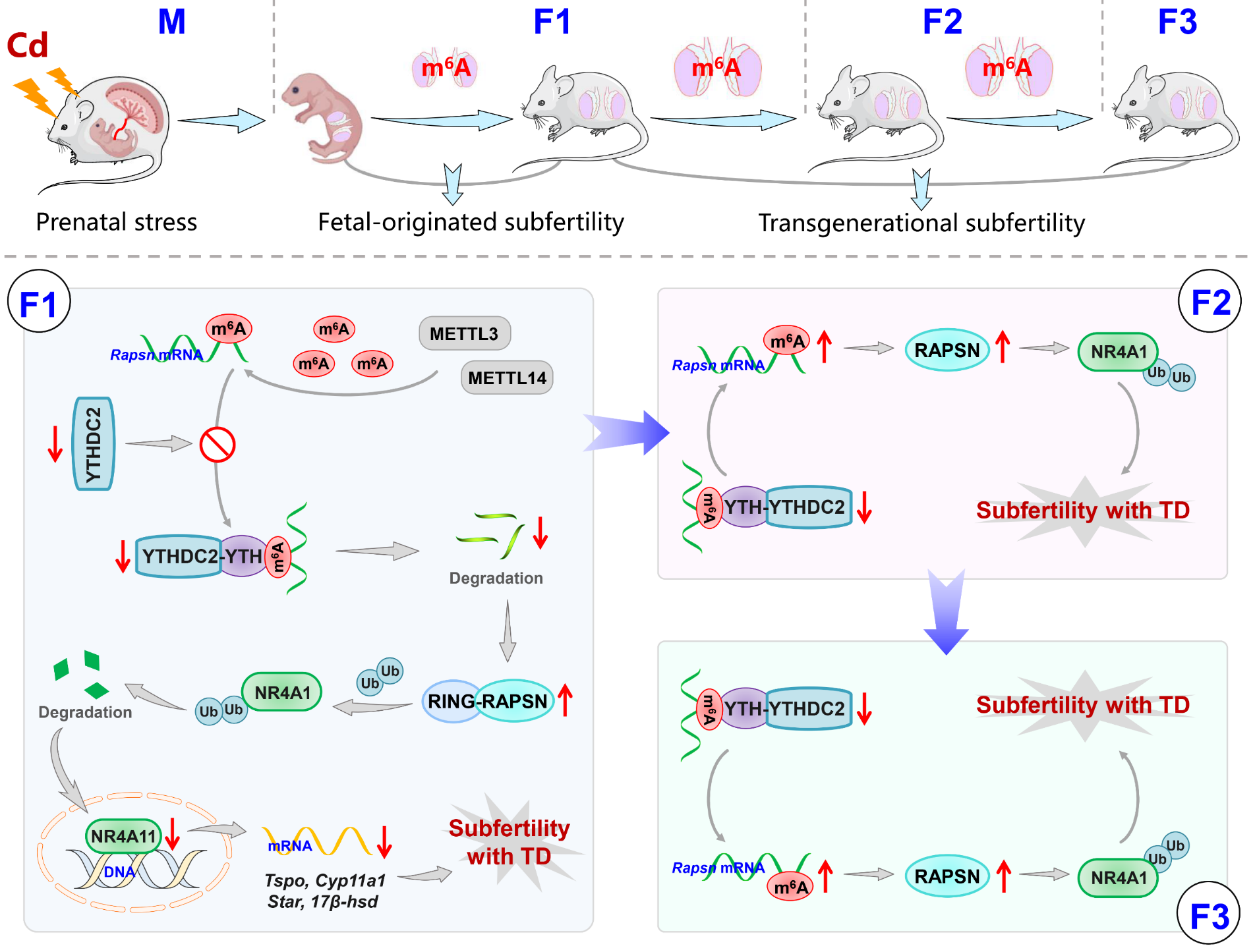


**Fig. S28 The schematic diagram that prenatal Cd exposure drives Rapsn m^6^A modification to enhance multigenerational susceptibility of male infertility.** M: maternal mice. F1, filial generation 1. F2, filial generation 2. F3, filial generation 3.

**Supplementary Materials and Methods**

**Reagents and plasmids**

CdCl_2_ (Cd, 202908) and *N*-acetylcysteine (NAC, A7250) were supplied by Sigma-Aldrich. Enzyme-linked Immunosorbent Assay Kit for T (76098C7FEF) and Dihydrotestosterone (DHT, CEA443Ge) were from Cloud-Clone Crop. MG132 (133407-82-6) was purchased from Medchem Express. Trypsin (27250-018, Gibco), Deoxyribonuclease I (DN25, Sigma-Aldrich) and Collagenase IV (C5138, Sigma-Aldrich) were used to separate the primary Leydig cells. Seq-Star^TM^ Poly (A) mRNA Isolation Kit for NGS RNA library preparation (AS-MB-006) was from Aksomics. RNA Fragmentation Reagents (AM8740) were provided by Invitrogen. Dynabeads^TM^ M-280 Sheep anti-Rabbit IgG (11204D) was from Thermo-Fisher. Opti-MEM (31985-062) from Gibco and Lipofectamine 3000 Reagent Taransfection (L3000-015) from Invitrogen were used in cell culture experiments. Actinomycin D (A408625) was from Aladdin. The detailed information about all antibodies was summarized in Excel Table S1 and S2.

**Plasmids and siRNAs**

Type 5 lentivirus encoding *Rapsn-*GFP (LV5-*Rapsn*-CopGFP), LV5-empty- CopGFP were obtained from GenePharma (Shanghai, China). The plasmids expressing *Rapsn^WT^* (wild type Rapsn mRNA) or *Rapsn^MUT^* (*Rapsn^All^*, *Rapsn^Site1^*, *Rapsn^Site2^*, *Rapsn^Site3^* and *Rapsn^Site4^*), adeno-associated type 9 virus encoding *Ythdc2* (AAV9-*Ythdc2*) and AAV9-empty were obtained from Genechem (Shanghai, China). The plasmids expressing YTHDC2^WT^ (wild-type YTHDC2 protein) or YTTHDC2^△YTH^ (YTHDC2 protein deleted YTH domain) and the plasmids expressing RAPSN ^WT^(wild type RAPSN protein) or RAPSN*^△RING^* ( RAPSN deleted RING-H2_Rapsyn domain) were synthesized in General Biol (Anhui, China). Mouse sapiens *Ythdc2* short interfering RNAs (siRs) were obtained from GenePharma, and the information of *Ythdc2* siRNAs was provided in Table S5.

**Cd measurement**

Cd concentrations in maternal sera, placentae and fetal sera were measured using graphite furnace atomic absorption spectrometry (GFAAS, TAS-990; Purkinje General Instrument Co., Ltd, Beijing, China) refer to a previous study ^1^. For placental samples, 100 mg of tissue was decomposed in prepared mixture containing 2 ml of concentrated HNO_3_ and 1 ml of 30 % H_2_O_2_ by incubating for 12 h at room temperature. Then, the digests were boiled to dryness and dissolved in 1 ml of 1.0% HNO_3_. The sera samples were diluted four times using 1.0% HNO_3_. Cd concentrations were detected using GFAAS. The loading volume of the solution was 10 μl.

**Evaluation of sperm count and motility**

The sperm count and motility was measured using an automatic sperm analyzer (Hamilton Thorne, USA) as our previous study described ^2^. The left epididymis was immediately collected after euthanasia. The epididymis was then cut into pieces in 3 ml of preheated medium. Next, the medium containing epididymal fragments was incubated at 37 °C for 5 min to make sperm completely released. Finally, 50 μL of perm suspension was dropped into a slide for the evaluation of sperm count and motility.

**Cell experiments**

TM3 cell, a mouse testicular Leydig cell line, was widely used in numerous studies about T synthesis ^3, 4^. The cells were obtained from Procell (CL-0234, China). The cells were cultured in DMEM/F12 medium containing 5% serum, 2.5% FBS, and 1% penicillin-streptomycin solution and incubated at 37^◦^C and 5% CO_2_. YTHDC2 was knocked down to evaluate the effect of YTHDC2 on the half-life of *Rapsn* mRNA and T synthesis. To confirm that reduced YTHDC2 inhibits T synthesis via the ubiquitin-proteasome system, the YTHDC2 knocked-down cells were treated with the proteasome inhibitor MG132 (5 μM) for 12 h. The cells were transfected with the plasmids overexpressed RAPSN^△RING^ (RAPSN deleted RING-H2_Rapsyn domain) or RAPSN^WT^ (wild type RAPSN protein) to investigate whether RAPSN promoted NR4A1 degradation through its RING-H2_Rapsyn domain, the ubiquitin ligases-related domain in RAPSN. The cells were transfected with plasmids expressing *Rapsn^WT^* (wild-type *Rapsn* mRNA), *Rapsn^All^* (*Rapsn* mRNA mutated all potential m^6^A modification sites), and *Rapsn^SiteN^* (*Rapsn* mRNA only mutated the potential m^6^A modification site *N*, where *N*=1, 2, 3 or 4) to identify the potential m^6^A modification site in *Rapsn* mRNA. TM3 cells were transfected with plasmids expressing YTHDC2^△YTH^ (YTHDC2 deleted YTH domain, the domain for recognizing m^6^A modification in YTHDC2) or YTHDC2^WT^ (wild type YTHDC2 protein) to investigate the role of YTHDC2 m^6^A reader in regulating Rapsn mRNA stability. The cells were treated with H_2_O_2_ (100 μM) for 0, 6, 12 or 24 h to evaluate whether oxidative stress mediates YTHDC2 reduction. The cells were treated with H_2_O_2_ (100 μM) for 12 h with or without MG132 (5 μM) pretreatment for 1 h to confirm that oxidative stress-mediated T synthesis repression via the ubiquitin-proteasome system.

**Overexpression and knockdown of genes in cells**

Lipofectamine^TM^ 3000 Transfection Reagent (Invitrogen) was used to transfect siRNAs or the gene-overexpressed plasmids to generate the TM3 cells with overexpression or knockdown of the indicated gene. For knockdown of genes, the Lipofectamine^TM^ 3000 Reagent in the reagent was used to transfect the *Ythdc2* siRNAs in TM3 cells according to the manufacturer’s guides. The sequences of *Ythdc2* siRNAs are summarized in the Excel Table S5. For overexpression of genes, the P3000^TM^ Reagent and the Lipofectamine^TM^ 3000 Reagent in the reagent were used to transfect the indicated gene-overexpressed plasmids in TM3 cells according to the manufacturer’s guides.

**Quantification of total m^6^A RNA levels**

The total m^6^A level in RNA was determined using an enzyme-linked immunosorbent assay (ELISA)-like method. Briefly, the m^6^A level in total RNA was colorimetrically detected using the EpiQuik m^6^A RNA Methylation Quantification Kit (Colorimetric, Epigentek) following the manufacturer’s guidelines. 200 ng of total RNA or positive control sample were added to the assay wells, respectively. An appropriate diluted concentration of the capture antibody, the detection antibody, the enhanced solution, and the developer solution was added to the wells. The absorbance at 450 nm wavelength was detected using a multimode reader (BioTek, USA). The level of m^6^A methylation was calculated using the standard curve.

**RNA stability**

The evaluation of mRNA stability was performed as a previous study ^5^. Briefly, Actinomycin (ActD, 5 μg/ml), an inhibitor for cellular transcription, was used to evaluate *Rapsn* mRNA stability. The total RNA was extracted from the cells with or without YTHDC2^WT^ or YTHDC2^ΔYTH^ overexpression, and the cells with or without YTHDC2 knockdown after ActD treatment for 0, 3 and 6h. The *Rapsn* mRNA was detected using qRT-PCR.

**Protein domain analysis**

The protein domain analysis was performed using the UniProt database (<https://www.uniprot.org>, release: 2024-01). The amino acid sequences of mouse RAPSN (UniProtKB: Q2M2N6) and YTHDC2 (UniProtKB: B2RR83) were obtained from the UniProt database. The composition and function of protein domains in RAPSN and YTHDC2 were found in the database. We downloaded the RAPSN and YTHDC2 protein structure PDB files from the AlphaFold 2.3 protein structure database (https://alphafold.ebi.ac.uk). The RAPSN and YTHDC2 cartoon models of the protein domains were also visualized using PyMOL 4.6.0 software.

**Enzyme-linked immunosorbent assay for testosterone and dihydrotestosterone**

The testicular T was extracted using ethyl ether according to our previous study ^6^. According to the manufacturer’s instructions, the concentrations of T, DHT, and E2 in mouse testes, sera, and cell media were determined using the Enzyme-linked Immunosorbent Assay (ELISA) Kit for T (76098C7FEF, Cloud-Clone Crop), ELISA Kit for DHT (CEA443Ge, Cloud-Clone Crop) and ELISA Kit for E2 (CEA461Ge, Cloud-Clone Crop), respectively. The T, DHT and E2 in sera were expressed as ng/ml. The T, DHT and E2 in testes were expressed as ng/mg protein.

**Immunoblotting**

The protein lysates were prepared using RIPA lysis buffer with a protease inhibitor. The protein concentration was measured using the BCA Protein Assay Kit (Thermo Fisher). After adding loading buffer, the samples were boiled. Immunoblotting was performed as previously described ^7^. In brief, the total proteins were separated using 12% SDS-PAGE and transferred into a PVDF membrane. The membrane was incubated in 5% skim milk for 1.5 h at room temperature to block the nonspecific binding site. Then, the membrane was incubated with primary antibody for 1.5-2.0 h at room temperature. The catalogue numbers, sources and dilutions of all primary antibodies were provided in Excel Table S1. After washing, the membrane was incubated with secondary antibody for 1.5-2.0 h at room temperature. The information about all secondary antibodies was provided in Table S2. After washing, the target protein signals were detected using digital imaging equipment (Bio-Rad). The images were quantitatively analyzed using the Image-Pro Plus 6.0 software.

**Immunofluorescent staining**

Immunofluorescent staining was previously described ^7^. In brief, the frozen testicular tissue was cut into 5 μm sections using a freezing slicer ((Leica). After washing for three times in PBS solution, the nonspecific binding sites were blocked via incubation in PBS containing 10% donkey serum at 37 ℃ for 1 h. The slices were then incubated with the primary antibodies overnight at 4 ℃and then washed for three times, followed by 1.5 h incubation with fluorescein-conjugated secondary antibodies at 37 ℃. All primary and fluorescein-conjugated secondary antibodies are listed in Excel Table S1 and S2. The immunofluorescent signals of target proteins were detected using a confocal microscope (Zeiss). The images were quantitatively analyzed using the ImageJ 2.0 software.

**RNA isolation and** **RT-qPCR**

The total RNA from testes and cells was extracted using TRIzol reagent (15596026CN, Invitrogen). The RNA quantification was performed using an ultra-microphotometer (DS-11, Denovix). 1000 ng of RNA was reverse transcribed into cDNA using Transcriptor First Strand cDNA Synthesis Kit (04896866001, Roche). Real-time RT-qPCR (RT-qPCR) was implemented to detect the expression of targeted mRNAs, such as *Tspo, Star, Cyp11a1, 17β-hsd, Nr4a1 and Rapsn*. *18S* was used as the internal control. The sequences of these primers were provided in Excel Table S3. Fast Start Essential DNA Green Master (060712001, Roche) was used for RT-qPCR in the real-time fluorescence quantitative PCR machine (LightCycler® 480Ⅱ, Roche). Comparative analysis of targeted mRNA levels was performed using the CT method.

**Assessment of DNA methylation in sperm and testes**

Genomic DNA was isolated using the Animal Tissue/Cell Genomic DNA Extraction Kit (D1700, Solarbio). DNA concentration and purity were assessed using the DS-11 Spectrophotometer (DeNovIX, USA). Global DNA methylation levels were quantified using the MethylFlash™ Global DNA Methylation (5-mC) ELISA Easy Kit (P-1030, EpigenTek). Each sample, containing 100 ng of DNA, was analyzed in a single well. A standard curve was generated concurrently using the provided controls with known methylation percentages (0.1% to 5%). Absorbance was measured at 450 nm using a microplate reader (BioTek, USA). The methylation percentage (5-mC%) for each sample was calculated based on the standard curve.

**Reference**

1. Ji, Y.L. *et al.* Effects of maternal cadmium exposure during late pregnant period on testicular steroidogenesis in male offspring. *Toxicol Lett* **205**, 69-78 (2011).

2. Xiong, Y.W. *et al.* Multigenerational paternal obesity enhances the susceptibility to male subfertility in offspring via Wt1 N6-methyladenosine modification. *Nature communications* **15**, 1353 (2024).

3. Wang, J.K. *et al.* Multiple transcriptomic profiling: p53 signaling pathway is involved in DEHP-induced prepubertal testicular injury via promoting cell apoptosis and inhibiting cell proliferation of Leydig cells. *J Hazard Mater* **406** (2021).

4. Zhao, Y. *et al.* SLC7A11 as a therapeutic target to attenuate phthalates-driven testosterone level decline in mice. *Journal of advanced research* (2024).

5. Wang, J.N. *et al.* Inhibition of METTL3 attenuates renal injury and inflammation by alleviating TAB3 m6A modifications via IGF2BP2-dependent mechanisms. *Science translational medicine* **14**, eabk2709 (2022).

6. Wang, T.T. *et al.* Environmental cadmium inhibits testicular testosterone synthesis via Parkin-dependent MFN1 degradation. *J Hazard Mater* **470** (2024).

7. Zhu, H.L. *et al.* Gestational exposure to environmental cadmium induces placental apoptosis and fetal growth restriction via Parkin-modulated MCL-1 degradation. *J Hazard Mater* **424** (2022).
